# A Multimodal, In Vivo Approach for Assessing Structurally and Phenotypically Related Neuroactive Molecules

**DOI:** 10.1021/acschemneuro.4c00426

**Published:** 2024-09-17

**Authors:** Matthew N. McCarroll, Elizabeth Sisko, Jung Ho Gong, Jinfeng Teng, Jack Taylor, Douglas Myers-Turnbull, Drew Young, Grant Burley, Lain X. Pierce, Ryan E. Hibbs, David Kokel, Jason K. Sello

**Affiliations:** † Department of Pharmaceutical Chemistry, University of California, San Francisco, San Francisco, California 94158, United States; ‡ Institute for Neurodegenerative Diseases, University of California, San Francisco, San Francisco, California 94158, United States; § Department of Neurobiology, University of California, San Diego, California 92093, United States; ∥ UCSF Weill Institute for Neurosciences Memory and Aging Center, University of California, San Francisco, California 94158, United States; ⊥ Department of Chemistry, Brown University, Providence, Rhode Island 02912, United States

**Keywords:** zebrafish, isoflavones, GABA, TSPO, phenotypic screening, structure activity relationship, paradoxical excitation, startle response, neuropharmacology, anesthetics, electrophysiology

## Abstract

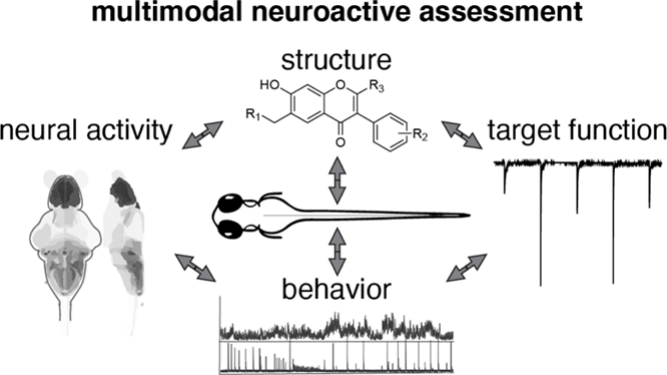

A recently reported behavioral screen in larval zebrafish for phenocopiers of known anesthetics and associated drugs yielded an isoflavone. Related isoflavones have also been reported as GABA_A_ potentiators. From this, we synthesized a small library of isoflavones and incorporated an in vivo phenotypic approach to perform structure-behavior relationship studies of the screening hit and related analogs via behavioral profiling, patch-clamp experiments, and whole brain imaging. This revealed that analogs effect a range of behavioral responses, including sedation with and without enhancing the acoustic startle response. Interestingly, a subset of compounds effect sedation and enhancement of motor responses to both acoustic and light stimuli. Patch clamp recordings of cells with a human GABA_A_ receptor confirmed that behavior-modulating isoflavones modify the GABA signaling. To better understand these molecules’ nuanced effects on behavior, we performed whole brain imaging to reveal that analogs differentially effect neuronal activity. These studies demonstrate a multimodal approach to assessing activities of neuroactives.

## Introduction

Neuroactive compounds (neuroactives) constitute a large group of clinically relevant yet poorly understood molecules that perturb nervous systems by engaging one or multiple targets. The polypharmacology of these molecules challenges the effectiveness of single-target in vitro studies, which are common in drug discovery and chemical biology. Even the assessment of multiple targets in vitro provides limited insights into their potential effects on neural circuits and behaviors. Thus, phenotypic approaches, like behavioral profiling, are valuable for exploring neuroactives with unknown targets or mechanisms.^1^ These methods have revealed that drugs with similar pharmacological properties often induce similar behavioral patterns.^2−4^ To complement these approaches, in vivo imaging of fluorescent markers using next-generation microscopy can assess neuronal activities within the central nervous system (CNS), underlying these behaviors.^5^ Advanced behavioral screening platforms now allow precise control, capture, and quantification of animal behaviors influenced by neuroactives.^4,6,7^

**Figure 1:**
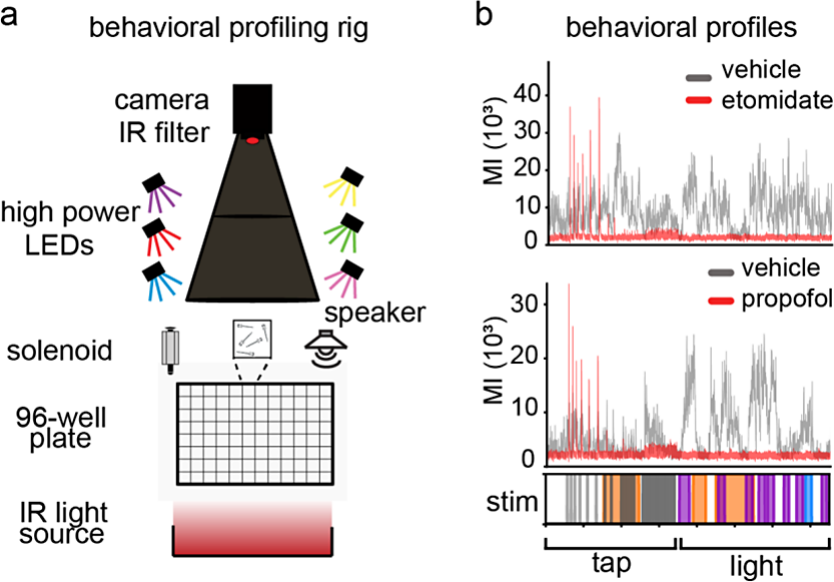
Behavioral profiling rig and examples. (a) Schematic of our custom behavioral profiling rig to record zebrafish larval behavior and deliver light and acoustic stimulus to multiwell plates. (b) Behavioral profile of animals responding to tap and light stimulus delivered at precise time points (*x*-axis) and activity is calculated as a motion index (MI) on the *Y*-axis. Animals are treated with either vehicle control (gray) or the anesthetic etomidate (red, top profile) or propofol (red, bottom profile) *n* = 4–12 wells per condition, 8 animals per well.

Zebrafish (*Danio rerio*) are an ideal model for connecting drug effects to behavior and neural activity changes. Larval zebrafish are particularly useful for in vivo studies of neuroactives due to the ease of imaging their CNS activity and behaviors. Our custom platform can generate and record responses from multiple animals per well in a 96-well plate, making it suitable for high-throughput experiments, including neuroactive compound testing^2,4^ (Figure 1a). At 7 days post fertilization (dpf), larval zebrafish exhibit stereotyped sensorimotor responses to stimuli (Figure 1b). For these studies, we performed behavioral profiling experiments involving the exposure of larval zebrafish to acoustic and light stimuli, recording their responses, and quantifying their movements as a motion index (Figure 1b, *y*-axis). Remarkably, unique behavioral profiles often correlate with distinct pharmacological treatments.^2,4,6^ This platform is a powerful tool for identifying novel compounds that mimic clinically used drugs. Previous studies have successfully screened over ten thousand compounds to identify anesthetic-related compounds^2^ and drugs that phenocopy first-generation typical antipsychotics like haloperidol.^8^ By connecting behavioral patterns from phenotypic screens with known neuroactives, new hypotheses can be generated.

The technologies for imaging cells, circuits, and behaviors in larval zebrafish can redefine how we discover and assess neuroactive small molecules. Known neuroactives targeting the γ-amino butyric acid-A (GABA_A_) receptor and GABAergic systems in humans are relevant in zebrafish due to their high sequence homology.^9−11^ The GABA_A_ receptor is a primary target for many neuroactives such as anxiolytics, hypnotics, sedatives, anesthetics and convulsants.^12,13^ This wide range of pharmacology reflects the complexity of the GABAergic system. The receptor’s pentameric structure, composed of 19 possible subunits, is expressed in various neuroanatomical locations within the CNS.^13,19^ It has been estimated that as many as 800 distinct GABA_A_ receptor subtypes might exist in the human brain.^14^ Furthermore, extrasynaptic GABA_A_ receptors alter the tone and dynamics of GABAergic signaling.^14,15^ This complexity makes traditional SAR studies of GABAergic neuroactives and GABA related phenotypes especially difficult. However, GABA_A_ receptor ligands used in research or as drugs have comparable effects on larval zebrafish.^2,16,17^ We suggest that zebrafish behavioral profiling provides an alternative framework for studying the complex pharmacology of neuroactives.

We previously described a phenotypic screen for molecules that mimic anesthetics like etomidate and propofol, known to engage the GABA_A_ receptor.^2^ Those anesthetics result in loss of motor activity (sedation) and an enhanced acoustic startle response (eASR, see Figure 1b). In a high-throughput phenotypic screen of over 10,000 compounds, we discovered an isoflavone (7013338) as a top hit, inducing sedation and robust eASRs in larval zebrafish.^2^ This compound also exhibited enhanced startle responses to light stimuli (Figure 3). A follow up fluorometric imaging plate reader (FLIPR) assay demonstrated this isoflavone as a GABA_A_ receptor ligand, aligning with the anesthetics it mimmics.^2^ To the best of our knowledge, ours was the first report of the neuroactivity of an isoflavone in a live animal. Our findings were corroborated by studies showing similar isoflavones as GABA_A_ receptor positive allosteric modulators (PAMs).^18^ Additionally, there are reports of natural product isoflavones having neuroactive, anti-inflammatory or neuroprotective effects, but many of these possess limited modifications to the core scaffold.^20−22^

In this proof-of-principle study, we pursued small molecules that are structurally similar to and behavioral phenocopies of a previously identified chemotype targeting the GABA_A_ receptor.^2^ Here we synthesize a library of 30 isoflavones based on these known ligands for screening in our behavioral profiling platform so that we could generate a structure–activity relationship (SAR) distinct from a traditional SAR, as we sought to identify distinct structural features that lead to changes in the behavioral profile (i.e., sedation, enhanced acoustic startle, enhanced light startle) rather than screening directly for ligand potency. We propose that this behavior-first approach is especially suited to the identification and study of neuroactive compounds, given their complexity. We found that among the 30 isoflavones tested, the enhanced responses to either light or acoustic stimuli could not be uncoupled from the sedating activity. Patch clamp recordings of HEK 293 cells expressing a human GABA_A_ receptor demonstrate that a subset of the molecules active in zebrafish were GABA_A_ PAMs. Neuroactives that did not potentiate the tested GABA_A_ isoform may interact with another isoform or another CNS receptor. To further tie this ligand engagement with behavioral outcomes whole brain imaging of larval zebrafish was performed. Neuroimaging of larval zebrafish confirmed the isoflavones’ distinct activities in different neuroanatomical regions, aligning with observed behaviors. Our multimodal approach in this SAR study of isoflavones offers a valuable strategy for discovering and early-stage characterization of neuroactive compounds.

## Results

### Synthesis and Evaluation of a Collection of Isoflavones via Behavioral Profiling

**Figure 2:**
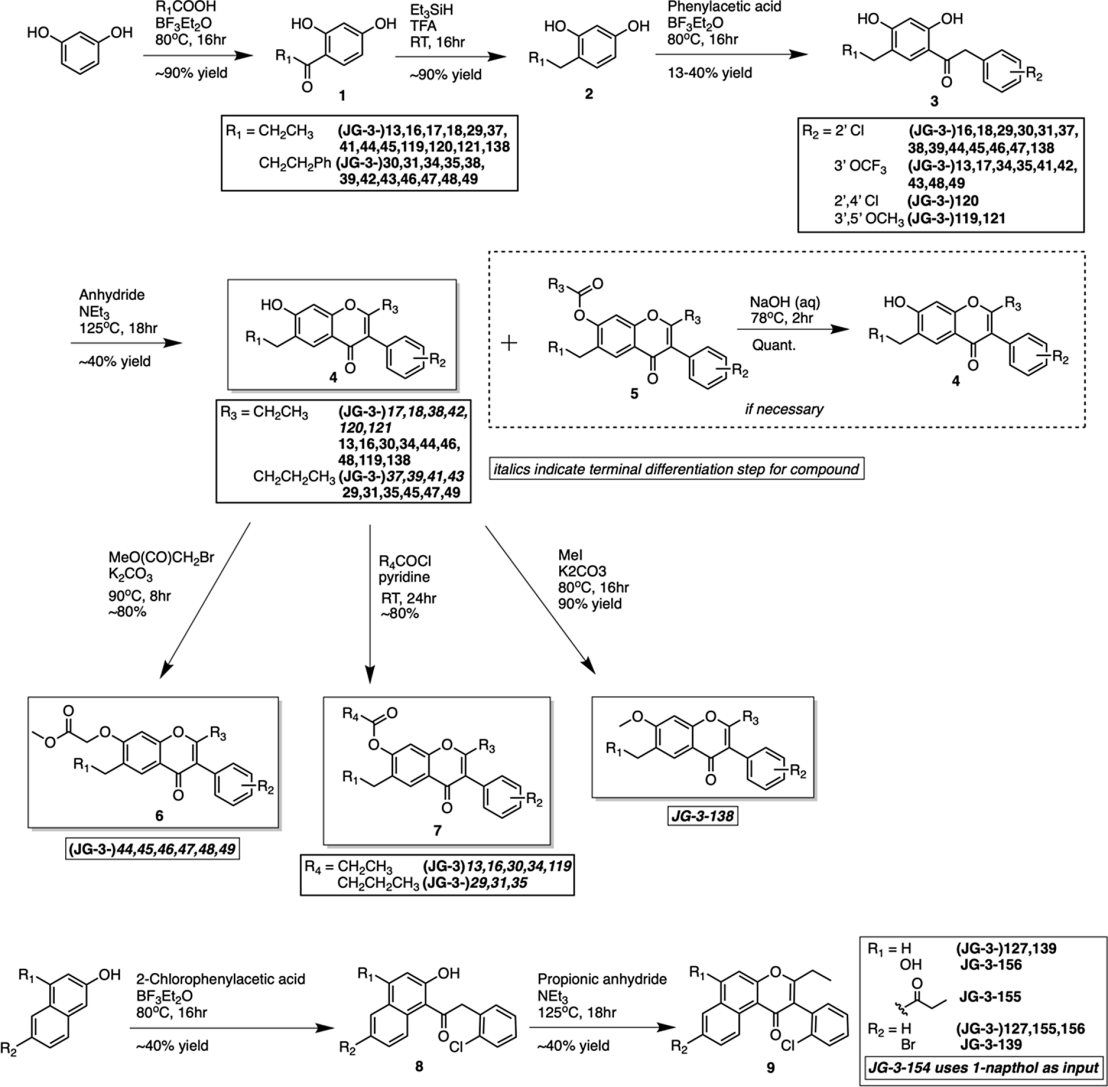
A multistep synthesis route for generating diverse isoflavones and related molecules.

**Figure 3:**
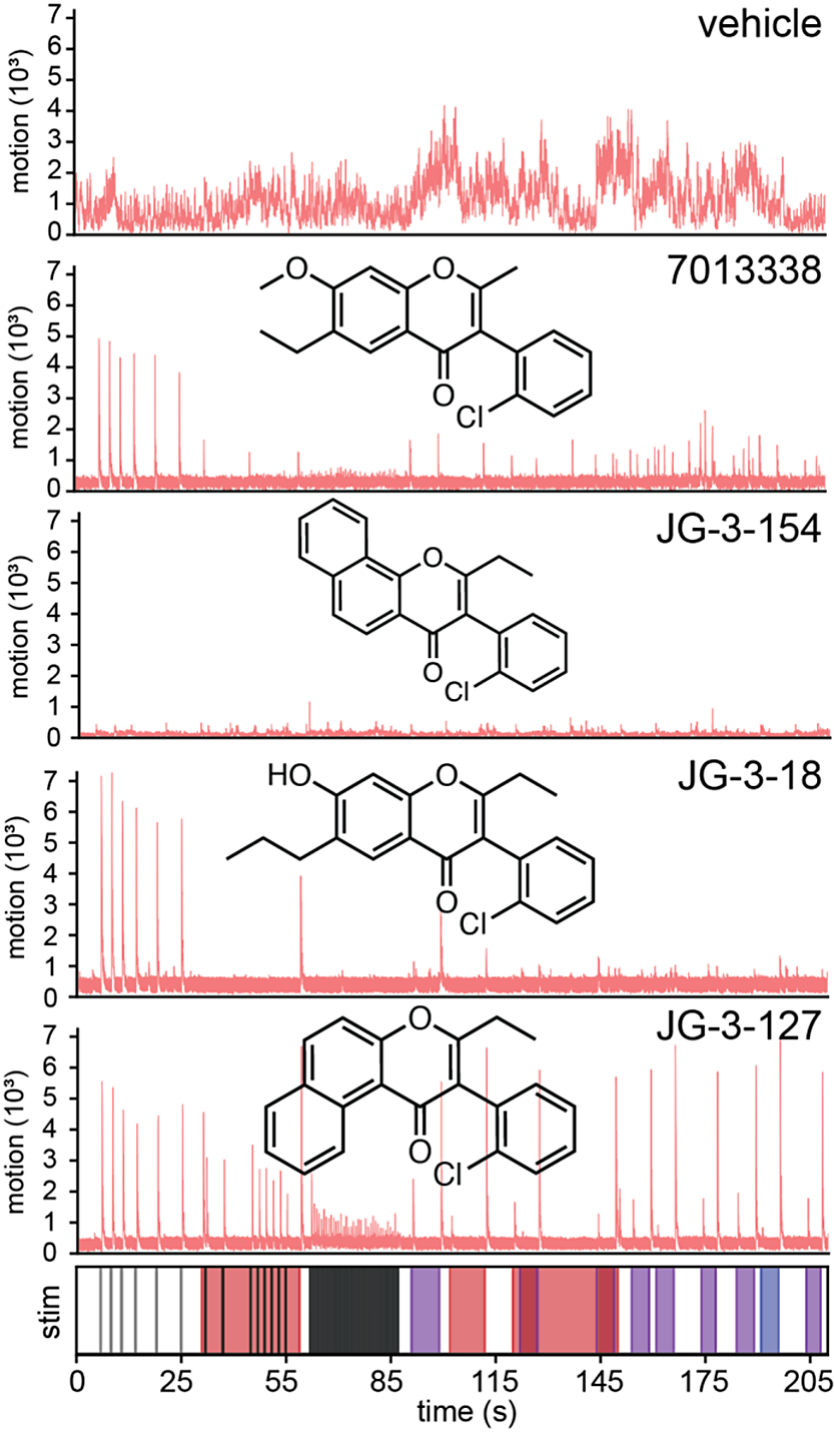
Different isoflavones modify zebrafish behavior in distinct ways. Behavioral profiles of larval zebrafish responding to stimulus over time in seconds (*x*-axis) activity expressed as a motion index (*y*-axis) for the following treatments: vehicle, the parent isoflavone (7013338) and 3 analogs that induce full sedation (JG-3-154), acoustic startle (JG-3-18) and acoustic startle and light startle (JG-3-127). Isoflavone structures are shown with their respective names and traces.

Our discovery of an isoflavone that effects sedation in larval zebrafish and enhances the animals’ responses to acoustic and light stimuli prompted many questions.^2^ We contemplated the possibility that sedation and the two different enhanced startle responses might be uncoupled from one another. Due to the complex nature in which neuroactive compounds modify behavior, we employed an in vivo behavioral profiling approach to gain insights into the structural determinants of neuroactivity. We synthesized 30 analogs of the screening hit (Table S1) and assessed their capacity to perturb the responsiveness of 7 dpf larval zebrafish to a variety of stimuli via behavioral profiling. Our collection included isoflavones having substituents differing in sterics, polarity, and hydrophobicity at the 2, 3, 7, and 6 positions of the isoflavone scaffold (Figure 2 and Table S1). We assessed the compounds’ ability to induce sedation and alter responsiveness to sound and light in animals (Figures 3 and S1). Specifically, we quantified the overall motor activity and the extent of the startle responses in our behavior-focused assessment of neuroactivity. Our methodology enabled us to correlate the compounds’ structures with their concurrent effects on multiple behavioral aspects, as opposed to conventional SAR studies that use in vitro assays to assess single metrics such as affinity, efficacy, or potency. Our findings indicated that individual compounds uniquely influenced sedation versus acoustic and light startle reflexes (Figures 3 and S1).

### Structure Activity Relationship Studies Identify Phenotypic Subclasses of Isoflavone Analogs

We developed indices to quantify the effects of three perturbations—sedation, eASRs, and light startle—summarized in Table S2. The sedation index was calculated from the average motion of animals exposed to various isoflavones normalized to a vehicle control group. Untreated animals had a baseline activity index of 100, with sedative compounds scoring lower. Subtracting this value from 100 yielded the sedation index, with values over 25.3 indicating significant sedation. This cutoff represents 1.5 standard deviations above the control group’s mean. Out of 30 synthesized isoflavones, 17 notably decreased motor activity in larval zebrafish, suggesting sedative properties (Table S2). Interestingly, these compounds predominantly featured a 2-chloro group at the 2’ position of the isoflavone core, despite diverse overall structures (Figures S2 and S3). To determine eASR and light startle indices, we quantified the maximum increase in animal responses resulting from treatment with each isoflavone regardless of concentration, as certain behavioral reactions to stimuli do not consistently follow a predictable dose dependence, likely due to the interplay of numerous physiological and neurological factors present in an organism (Figure S4). The eASR and light startle indices were normalized to the most effective molecule. Active molecules had scores above 56.2 for eASRs and 69.7 for light startle (1.5 SD from the mean of the vehicle control).

For eASRs, we found that 11 of the 30 isoflavone analogs were active (Table S2 and Figure S1). All compounds that affected eASRs were also sedatives. It is noteworthy that we did identify a small subset of compounds that were sedating without influencing eASRs, which suggests that the two activities can be uncoupled (these include JG-3-154 and JG-3-119, Table S2 and Figure S3). In the light startle response, only two compounds among the 30 analogs induced the phenotype (JG-3-127 and JG-3-138, Table S2 and Figure S1b). Both of those compounds induced a stronger light startle response than the parent compound 7013338 but also exhibited strong activities as sedatives and were eASR inducers. JG-3-127 exhibited the most potent influence over the light startle response (Figure 2). Interestingly, this response was not observed in any other benzannulated isoflavones. In contrast, the other light startle compound, JG-3-138, differed from the most active eASR molecule, JG-3-18, only in the substitution at position 7 (methoxy instead of hydroxyl). The compound JG-3-41 had no changes in animal behavior at any assessed concentration and was thought to be null (Figure S5). This compound had an increased alkyl chain length at the 2 position and no modification of the hydroxyl group at the 7 position (Figure S5). Uniquely, JG-3-154 caused robust and full sedation of the animals across a wide concentration range and did not affect paradoxical excitation to either acoustic or light stimuli at the evaluated concentrations (1.5–100 μM, Figure S4), though it is a constitutional isomer of JG-3-127.

While there are not many strong structural correlates that would predict neuroactivity with respect to substituent position and identity on the isoflavone scaffold, we were intrigued to find that the isoflavones could be categorized with respect to activity. We see three distinct activity categories of the compounds- 7 that effect sedation without affecting eASR, 10 compounds that effect both sedation and eASRs and three compounds (including the parent molecule) that effect sedation, eASRs, and the light startle response (Figures S2, S3, and 3). Curiously, the enhanced responses to either light or acoustic stimuli could not be uncoupled from the sedating activity. Moreover, enhancement of the light startle response could not be uncoupled from eASRs and sedation.

### Subset of Isoflavones Enhance GABA_A_ Channel Activity

**Figure 4:**
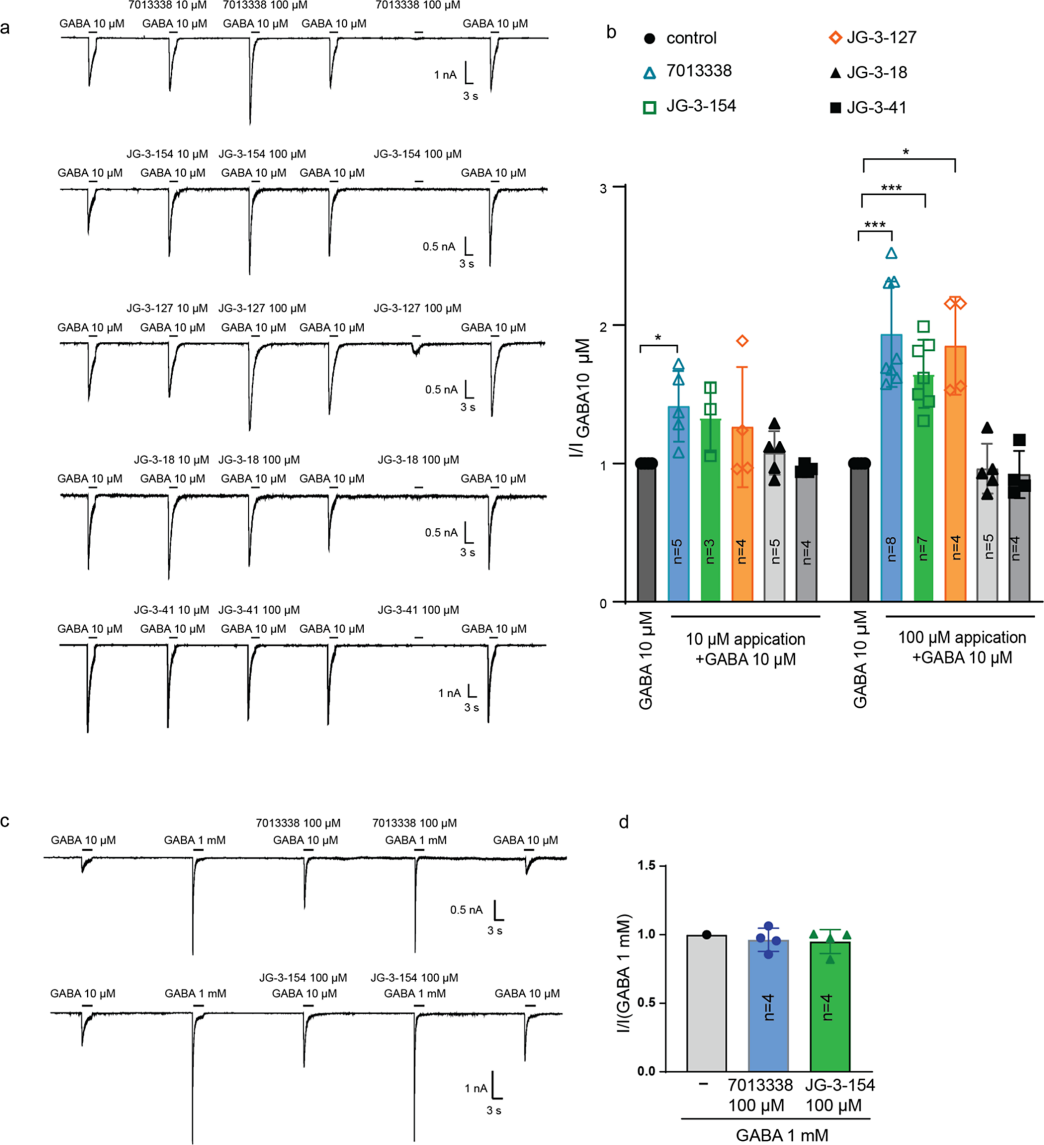
GABA_A_ electrophysiology in response to isoflavone treatment. (a) Traces of inward currents of HEK 293 cells expressing human α_1_β_2_γ_2_ GABA_A_ receptors in response to GABA alone (10 μM), GABA and isoflavone at both 10 and 100 μM and isoflavone alone (100 μM). Recordings were made for the parent 7013338, and 4 analogs (JG-3-154, JG-3-127, JG-3-18, JG-3-41) (b) Bar graph summary of potentiation for the isoflavone treatments at both 10 and 100 μM (I/I for GABA at 10 μM). (c) Recording as in (a) in response to GABA alone (10 μM), and to high concentrations of GABA (1 mM) coapplied with either the parent molecule 7013338 or analog JG-3-154 at 100 μM. (d) Bar graph summary for the isoflavone treatments at 100 μM (I/I for GABA at 1 mM).

The tested isoflavone derivatives show that the activities observed in the parent compound can be retained or lost, while some derivatives lead to an increase in potency for a given behavioral phenotype. In some cases, we observe the induction of entirely new phenotypes, as in the case of enhanced light startle. This activity could arise from binding to the GABA_A_ receptor and/or with other targets. We previously found the parent molecule 7013338 to be a relatively potent α_1_β_2_γ_2_ GABA_A_ PAM via FLIPR assay.^2^ Here, we performed patch clamp electrophysiology on a subset of isoflavones that induce different behavioral phenotypes. We compared the activity of test isoflavones to the parent molecule and the behavioral null utilizing a cell line expressing the α_1_β_2_γ_2_ GABA_A_ receptor. Indeed, the parent molecule 7013338, JG-3-127 (optimized eASR and light induced paradoxical excitation), and JG-3-154 (full sedation, Figure 3) all significantly potentiated the currents induced by 10 μM GABA (Figure 4a,b). Notably, these compounds exhibited a slow wash-off rate from the receptor, which affected subsequent GABA responses, possibly due to membrane partitioning. Interestingly, JG-3-127 exhibited weak agonist activity at 100 μM in the absence of GABA (Figure 4a), whereas 7013338 and JG-3-154 did not exhibit agonist activity at the concentrations tested. Additionally, 7013338 and JG-3-154 did not enhance the currents induced by saturating 1 mM GABA, indicating that these compounds act as PAMs that increase GABA potency without altering the maximum response (Figure 4c,d). In further agreement, JG-3-41, which exhibits no effect on behavior in larval zebrafish (Figure S4), fails to potentiate GABA in the electrophysiological experiments (Figure 4a,b). However, JG-3-18, an optimized eASR compound (Figures 3 and S1a), did not demonstrate any noticeable enhancement of GABA signaling with the α1β2γ2 receptor isoform (Figure 4a,b). This divergence underscores the distinct outcomes that can emerge when comparing in vitro studies of neuroactive compounds with their in vivo results. Under traditional SAR campaigns where optimization at the receptor alone evaluates a compound’s activity, this ligand would have been determined inactive; however, this molecule not only phenocopies the parent molecule 7013338 but has increased activity for eASRs and sedation by phenotypic assessments (Figures 3, S1a, and S4), highlighting the utility of our behavioral SAR approach in assessing neuroactive compounds.

### Whole Brain Imaging of Pharmacologically Treated Zebrafish Larvae

The multiplicity of GABA_A_ receptors in both humans and larval zebrafish and the technical challenges of in vivo patch clamping or mutagenesis experiments ruled out the utility of biophysical or genetic methods for attempting to explain the behaviors. We then sought to reconcile the varied behavioral effects of the isoflavones which induced a light startle response, via whole-brain imaging in larval zebrafish. In our previous report detailing the behavioral screen that identified 7013338, we described the occurrence of eASRs.^2^ Here we present the first account of an enhanced light startle response, mirroring the paradoxical excitation of eASR to a new stimulus. We predicted that the compounds would exert distinct changes in neuronal activity in discrete neuroanatomical locations that could be distinguished by generating a neural activity map. This inquiry is crucial, given the observed differences in drug effects on behavior and the complex nature of GABAergic pharmacology. Detection of pERK can be used to identify active neurons and neural networks of zebrafish, allowing us to generate whole-brain neural activity maps in pharmacologically perturbed samples. The resultant maps outline anatomical areas and specific neurons in DMSO, 7013338, JG-3-41 (null), JG-3-127 (optimized eASR and light startle response), and JG-3-154 (full sedation) treated animals. pERK in active neurons can be detected on the order of minutes.^5^

**Figure 5:**
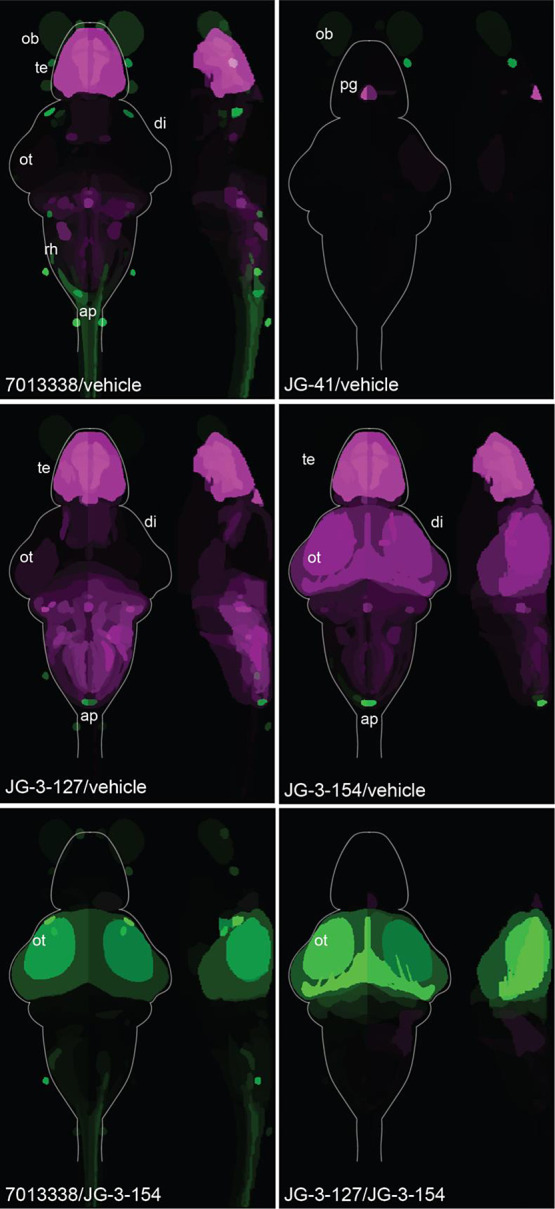
Whole brain neural activity maps of larval zebrafish in response to different isoflavone treatments. Top 4 panels are pairwise comparisons of animals treated with each of 4 phenotypically distinct isoflavones compared to vehicle treated controls. Illustrated are neuroanatomical regions that either increase (green) or decrease (magenta) in activity due to pharmacological treatment. Bottom two panels compare the parent molecule (7013338) or JG-3-127 (light startle molecule) to JG-3-154, the fully sedating isoflavone. *N* = 10 animals per condition. Abbreviations: ob, olfactory bulb; te, telencephalon; di, diencephalon; ot, optic tectum; rh, rhombencephalon; ap, area postrema; pg, pineal gland.

Initially, we compared each isoflavone treatment to a vehicle control (Figure 5). The parent molecule showed a significant reduction in neural activity in the telencephalon and hindbrain/rhombencephalic regions, while the optic tectum was not significantly reduced compared with awake animals, indicating sustained activity in the visual processing center of the larval brain. Additionally, there was increased neural activity in the mesencephalon retinal arborization field 7 and the spinal cord in animals treated with the parent molecule (Figure 5, Tables S3, and S4). In contrast, JG-3-41 treatment showed neural activity patterns like those of vehicle-treated animals with minimal changes observed, except for a potential reduction in pineal gland activity (Figure 5). Animals treated with JG-3-127, which exhibited behavior and patch clamp results similar to those of the parent molecule, displayed a neural activity profile closely resembling that of the parent molecule compared to the vehicle (Figure 5 and Table S5). Notably, there was stronger neural inhibition observed in the brains of JG-3-127-treated animals compared with those treated with the parent molecule, specifically in broad regions of the hindbrain and the reticulospinal track (Figure 5). Finally, examination of the pERK signal for JG-3-154, the isoflavone associated with full sedation, revealed a more widespread attenuation of neural activity, including in the optic tectum (Figure 5 and Table S6). These findings align consistently with the observed behavioral profiles (Figure 3).

Then, we compared the parent molecule and the optimized light startle molecule JG-3-127 to the full sedation isoflavone (JG-3-154). The neural activity maps clearly demonstrate how these compounds suppress much of the brain’s activity in larval zebrafish, except for the optic tectum, which shows significantly increased activity likely due to the light-induced paradoxical excitation caused by these molecules (Figure 5, Tables S7, and S8). Interestingly, JG-3-127 exhibits greater activation of the optic tectum compared with the parent molecule in these comparisons (Figure 5 and Table S8). The finding is consistent with the stronger light startle behavior observed in JG-3-127 compared to 7013338 (Figures 3, S1, and Table S2). In summary, our use of behavioral profiling and whole-brain imaging reveals nuanced changes in neuronal activity among closely related compounds that would be challenging to fully elucidate using a biophysical approach alone for SAR studies. Employing these methods together will significantly enhance our understanding of neural activity during the study of complex neuroactive molecules that modulate intricate neural signaling pathways such as the GABA system. Studies aimed at improving GABAergic pharmacology should consider the pleiotropic effects that could arise from these ligands at different GABA_A_ stoichiometries and neuroanatomical sites in the brain. Utilizing in vivo methodologies capable of monitoring both behavior and neural activity enables further exploration of the potential of new neuroactive ligands beyond their effects on single receptors.

### Visual System’s Role in the Isoflavone-Induced Light Startle Behavior

**Figure 6:**
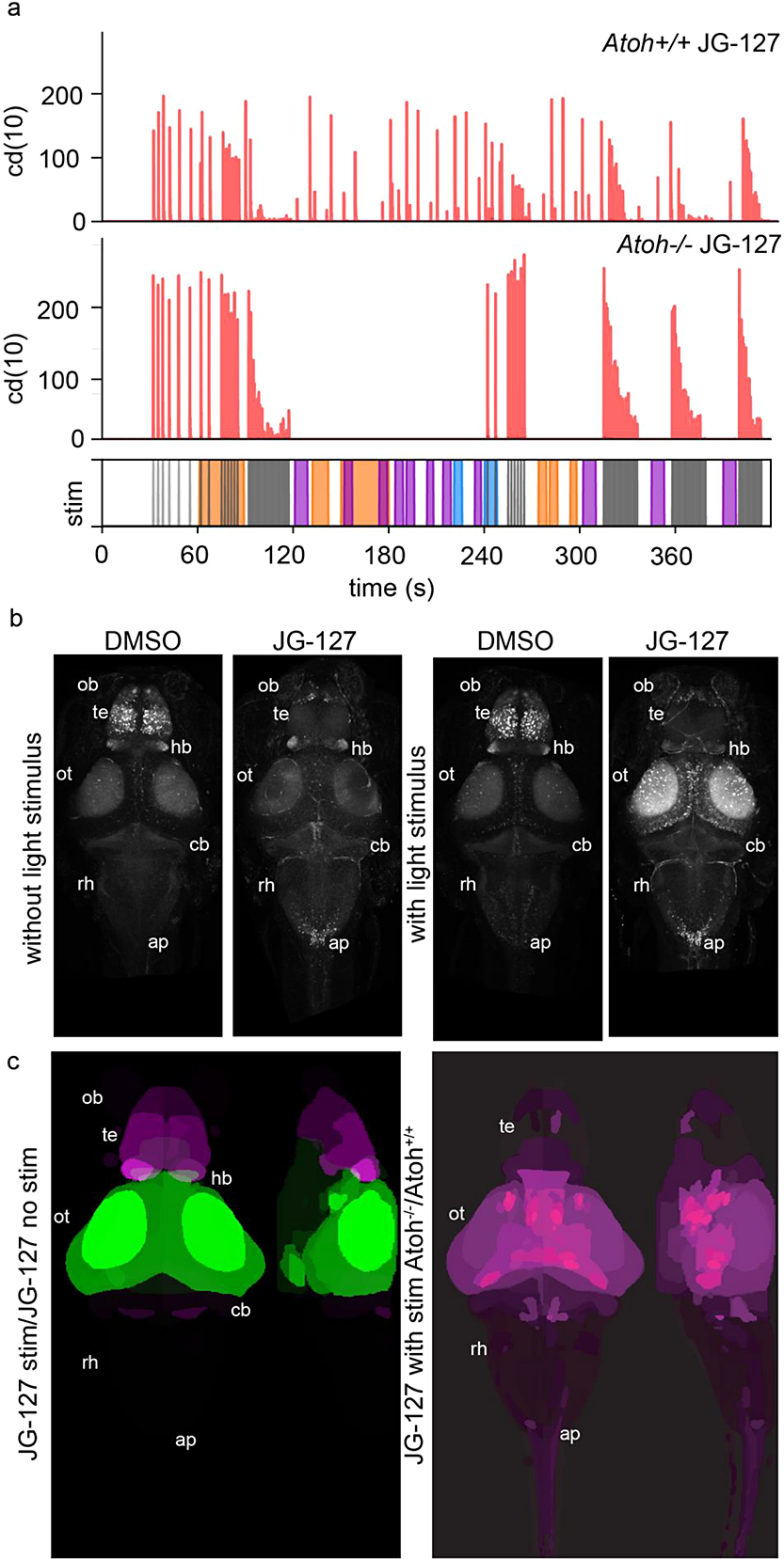
Assessment of light startle behavior and neural activity in genetically blind (*Atoh–/–*) animals compared to wild type siblings. (a) Behavioral profiles of animals treated with the acoustic and light startle isoflavone (JG-3-127) in wild type siblings (top traces) compared to blind mutant siblings (atoh–/– bottom trace). Battery consists of tap stimulus (30s-120s and 320s-end) and light stimulus (120s-240s and 280s-300s). (b) Averaged raw pERK channel whole brain images without (left side) and with (right side) light stimulus comparing vehicle to JG-3-127 treated animals. (c) Whole brain neural activity maps of larval zebrafish in response to different stimulus or genetic background. Left panel is a pairwise comparison of animals treated with JG-3-127 comparing animals exposed to light stimulus or no light stimulus. Right panel is JG-3-127 treated animals comparing wild type siblings to functionally blind *atoh7–/–* mutants to reveal neuroanatomical regions that either increase (green) or decrease (magenta) in activity due to JG-3-127 treatment and visual stimulus or genetic ablation of the visual senses. *N* = 10 animals per condition. Abbreviations: ob, olfactory bulb; te, telencephalon; hb, habenula; di, diencephalon; ot, optic tectum; cb, cerebellum; rh, rhombencephalon; ap, area postrema.

To understand how these isoflavones are influencing increased responses to light stimuli, we asked the question, is the zebrafish retina required for these behavioral responses? This question is of particular interest due to the presence of extra-ocular photoreceptors or opsins in the larval zebrafish capable of initiating motor activity.^23,24^ In addition, photochemical irritants like optovin or TRPswitch have been discovered in zebrafish behavioral screening campaigns and can initiate motor responses due to light induced changes of the molecule at nonphotoreceptive targets like TRPA1.^25−27^ We utilized a genetic ablation approach to determine the potential role of the retina in light startle responses after JG-3-127 treatment. The Atoh7 transcription factor is required for the proper development of retinal ganglion cells (RGCs).^28^ In a homozygous null of this gene, both a functioning retina and the optic tectum (the primary visual processing center of the larval zebrafish brain) are present; however, the RGCs that connect the two do not develop properly, generating a functionally blind mutant. We found that in wild type and heterozygous siblings, there was a robust light startle response in JG-3-127-treated animals (Figure 6a), however, in the homozygous mutants, light startle response to all wavelengths of light stimulus was completely ablated, while eASRs remained (Figure 6a). The identification of mutant and wild-type siblings was readily determined both by the visual phenotypic difference of increased pigmentation in the blind mutant, and through genotyping using PCR amplification of the mutated region and the disruption of the *StuI* restriction enzyme cut site^28^ (Figure S6). These data indicate that the isoflavone light startle response is mediated by the retina and requires functional RGCs to relay this signal to the CNS. We then compared vehicle-treated wild-type animals to JG-3-127 treatments with and without light stimulus and assayed for pERK in the brains and specifically the optic tectum (Figure 6b and Table S9). These conditions show a significant increase in pERK signal in the optic tectum in light stimulus and JG-3-127 treatment conditions. We then looked at whole brain activity maps in the Atoh7 mutant animals to determine if changes in neural activity in JG-3-127 treatment were due to retinal inputs or if there were some orthogonal inputs from other light sensing substrates in the animal that could activate the optic tectum (Figure 6c and Table S10). In these conditions the mesencephalic region including the optic tectum as well as the thalamus was significantly decreased when compared to wild-type siblings, indicating these anatomical regions are specific to stimulus, drug treatment, and a functioning zebrafish eye (Figure 6c and Table S10). Here, we conclude that the paradoxical excitation to light response observed with JG-127 is mediated by the visual system of the zebrafish.

## Disscussion

Understanding neuroactive compounds and their capacity to modulate behavior in animals could provide insights into the structure and function of CNS receptors. Neuroactive compounds alter neuronal function to change how an animal responds to stimuli. In this context, GABAergic sedatives and anesthetics are interesting examples. They are invaluable pharmacological tools in the clinic that prevent patients from feeling anxious or remembering painful experiences during procedures, such as surgeries and diagnostic tests. These psychophysical states are induced by the administration of distinct pharmacological agents that can cause a range of behavioral outcomes from anxiolysis, sedation, and hypnosis to amnesia and full loss of consciousness. Here, we describe a study wherein behavioral profiling, whole-brain imaging, and biophysical assays are used to build a “structure-behavior relationship” in a class of novel neuroactive isoflavones. Previous pharmacological studies have identified compounds that enhance startle reflexes in zebrafish larvae induced by sound.^2^ It is noteworthy that we have identified a novel isoflavone that enhances a visual startle response. Our finding of a molecule that enhances startle responses to both light and sound stimuli implies polypharmacology. This may be related to the numerous GABA_A_ ion channel subtypes that are the targets of many neuroactive drugs, or some other CNS receptor. Interestingly, some of the canonical GABA_A_ ligands such as benzodiazepines are known to interact with a mitochondrial transporter protein (TSPO) originally termed the peripheral benzodiazepine receptor due to its well documented binding of the ligand.^29^ In fact, radioligand displacement studies performed through the psychoactive drug screening program (PDSP) of our own initial isoflavone hit 7013338 also indicated that TSPO is engaged by this molecule.^2^ Furthermore, tested TSPO reference ligands were able to induce eASR in larval zebrafish.^2^ While this interaction is well recognized, the extent to which it may influence neuroactivity and the behavioral phenotypes associated with these ligands is not fully understood. However, it is known that TSPO controls the synthesis of neurosteroids like pregnenolone and allopregnanolone, which enhance GABA_A_ activity.^30^ Chemically, it has been well evidenced that small changes in benzodiazepines can lead to significant changes in ligand binding, as in the case of diazepam and Ro5-4864.^31^ The latter is solely a TSPO ligand despite the only structural difference being the addition of a single chlorine to the phenyl ring of diazepam, though it still displays neuroactive properties.^32−34^ JG-3-127, the molecule with light startle activity did show unique electrophysiological properties at the GABA_A_ receptor distinct from both the parent molecule and the fully sedating molecule. It is possible that some of these gained activities could be mediated through other targets like TSPO.

While many of the findings from biophysical assays aligned with our behavioral data, not all compounds showed GABA potentiation. Specifically, JG-3-18 was positive for sedation and eASRs in vivo but was not an apparent PAM at the GABA_A_ isoform tested. Potential reasons for this divergence may be related to differing subunit specificity, interactions with other targets or influencing GABAergic signaling indirectly from GABA_A_ engagement such as modulating TSPO, a known target of the parent molecule, which is known to control synthesis of neurosteroids that enhance GABA_A_ activity.^35^ This discrepancy illustrates that single-receptor studies do not fully predict neuroactive compounds’ behavioral outcomes in a whole organism and further emphasizes the need for a comprehensive drug development process that includes a range of studies to better understand the pharmacodynamics and pharmacokinetics of neuroactive compounds.

The overall sedative effects of these compounds observed in our behavioral assays were corroborated by our neural imaging studies. In general, we see reduced activity in various brain regions, particularly in the diencephalon and the pineal region for JG-3-127 and multiple regions of the telencephalon, mesencephalon, and rhombencephalon for JG-3-154 as compared to vehicle control. Both compounds showed decreased activity in telencephalon regions including dopaminergic and GABAergic neurons in the pallium and subpallium. Notably, the compound JG-3-127 led to increased activity in limited neuroanatomical regions compared to the vehicle control, including the olfactory system and the area postrema. Interestingly, known GABAergic anesthetics that induced paradoxical excitation in the form of eASR, such as etomidate, we found previously to also enhance the activity in the area postrema.^2^ Moreover, anatomical and genetic analyses identified the eye, RGCs, and optic tectum as being necessary for light startle behaviors. This was of interest due to previous drug screening campaigns that have identified photochemical irritants capable of initiating motor activity.^25−27^ In normal zebrafish, there was a strong light startle response following JG-3-127 treatment. However, in the mutants, responses to all light wavelengths were completely abolished while the acoustic response remained intact. These findings demonstrated that the isoflavone-induced light startle response depends on the retina and functional RGCs to transmit signals to the CNS. Furthermore, the mesencephalic region, including the optic tectum, showed significant decreases in mutants compared to normal siblings, suggesting that these anatomical regions are specific to the stimulus, drug treatment, and a functioning zebrafish eye. Interestingly, distinct GABA_A_ isoforms are known to be expressed in the retina of the zebrafish including the GABA_A_ Rho receptor which is a subclass of GABA_A_ receptors composed entirely of rho subunits as well as more canonical GABA_A_ receptors which include α5 and α6 subunits.^9,36,37^ It is possible that promiscuous interactions of certain isoflavones could impart their light startle activity through engagement with these different GABA_A_ isoforms enriched in the eye. We are currently working on follow up studies to better characterize the behavior, neural circuitry and further explore possible targets underlying this new phenomenon of light induced paradoxical excitation. Additional studies are needed to determine this phenotype’s possible utility in drug discovery efforts as well as the potential of these isoflavone probes for accelerating studies aimed to understand the molecular pathways and neural circuitry of defensive startle behaviors.

SAR studies typically examine how the molecule’s structure affects its interaction with specific biological targets, often focusing on a singular or subset of biomolecules. However, in the context of neuroactive agents, we emphasize the need for a comprehensive drug development process that includes a range of in vivo studies to understand fully the relationship of compound structure with the observed effect on behavior. The therapeutic and side-effect profiles of drugs can only be fully determined through in vivo studies, which can capture the complexity of biological systems and the subtleties of drug behavior. Typically, such studies are integrated at late stages of drug development pipelines using advanced mammalian systems, which are more costly and lower throughput. Implementing studies with larval zebrafish during hit discovery and initial characterization could expedite this development process. Establishing workflows that include in vivo behavioral profiling and whole brain imaging into early-stage drug discovery efforts could yield a more comprehensive perspective of candidate molecules vital for accurately predicting therapeutic potential and side effects profiles of neuroactive drugs.

## Methods

### Fish Maintenance, Breeding, and Chemical Treatments

Up to 10,000 fertilized zebrafish embryos were collected per day from group matings of wild-type zebrafish (Singapore). Larvae were raised on a 14/10 h light/dark cycle at 28 °C until 7 dpf. Larvae were anesthetized with cold egg water^38^ and distributed 8 animals per well into square 96-well plates (GE Healthcare Life Sciences). Plates were incubated at room temperature for 30 min for animals to become active. Compound stock solutions were applied directly to the egg water, and larvae were incubated with the drug at room temperature for 1 h before behavioral analysis. The zebrafish-related procedures were conducted according to established protocols approved by UCSF’s Institutional Animal Care and Use Committee (IACUC) and in accordance with the Guide to Care and Use of Laboratory Animals (National Institutes of Health 1996).

### Compounds and Treatments

All chemical libraries were dissolved in DMSO. Controls were treated with an equal volume of DMSO. Retesting of compounds was validated in 4–12 replicate wells. For dose-response behavioral assays, compounds were tested at 7 concentrations that ranged from 0.15- 100 μM, unless otherwise indicated.

### Automated Behavioral Phenotyping Assays and Calculations

Digital video was captured at 25 frames per second using an AVT Pike digital camera (Allied Vision). Each assay duration was 30–120 s and consisted of a combination of acoustic and light stimuli as described.^2,4^ Low (70 db) and high (100 db) amplitude acoustic stimuli were delivered using push-style solenoids (12 V) to tap a custom-built acrylic stage, where the 96-well plate was placed. Acoustic stimuli were recorded using a contact microphone (*Aquarian Audio Products*, model# H2a) and the freeware audio recording software Audacity (http://www.audacityteam.org). Stimulus volume was measured by using a BAFX 3608 digital sound level meter (BAFX Products). Light stimuli were delivered using high-intensity LEDs (LEDENGIN) at UV (355 nm, 875 mW), violet (405 nm, 11 μW/mm^2^), green (525 nm, 657 mW), blue (560 nm, 18 μW/mm^2^) and red (650 nm, 11 μW/mm^2^) wavelengths. Stimuli and digital recordings were applied to the entire 96-well plate simultaneously. Instrument control and data acquisition were performed by using custom scripts written in MATLAB and Python. The zebrafish motion index (MI) was calculated as follows: MI = sum(abs(frame*_n_* – frame_*n*–1_)). Normalized MI (nMI) was calculated as follows: *n*MI = (MI-min(MI))/max(MI)). For motion estimation expressed as a CD(10), the value was estimated as the count of pixels that changed from the previous frame by intensity ≥10/255.

### Calculation of the Activity and Sedating Indices

The average activity (*A*) was calculated as follows with the conditions being equivalent to vehicle or compound treatments including all of its tested concentrations.
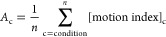


The activity was then normalized to that of the vehicle control.
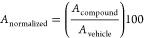


The sedating index was determined by subtracting each of the normalized activities from 100.
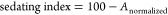


### Calculation of eASR and Light Startle Indices

Both indices for the respective stimuli were calculated as follows
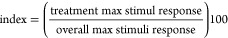


### Whole-Mount Immunostaining, Image Registration and Fluorescence Intensity Averaging

Whole-mount immunostaining and image registration were performed as described.^5,39^ The following antibodies were used: α-tERK (1:500, Cell Signaling) and α-pERK (1:500, Cell Signaling). Whole-mount fluorescent images were obtained using a Leica SP8 or a Leica Stellaris confocal microscope. Image processing was performed in ImageJ. A previously described GUI for the Computational Morphometry Toolkit^40,41^ was used to perform image registration of all analyzed immunostained brains aligned to a reference brain. Multiple brains from each condition were then averaged using custom-written Matlab scripts to obtain a representative neural activity image. Pairwise statistical analysis of anatomical regions for increase or decrease in neural activity was performed using the Z-brain atlas in conjunction with a previously published Matlab software package.^5^ To ensure biomarker accumulation and the full effect of the isoflavones, animals were treated with the respective compounds or vehicle control for 1 h. Subsequently, they underwent a 10 min behavioral assessment involving acoustic and light stimuli presented at 10 s intervals to prevent habituation. Following the behavioral experiment, animals were immediately fixed for pERK immunohistochemistry and imaged by high-resolution whole-brain confocal microscopy. The acquired images were processed and aligned to a reference brain using the computational morphometry toolkit for image registration. To reduce noise and reliably identify active neurons relevant to the behavior under investigation, multiple registered brains from each experimental condition (*n* = 10 per condition) were averaged. These neural activity maps were then aligned with a larval zebrafish brain atlas to identify regions affected by the pharmacological treatments. Brightness and contrast were adjusted using Fiji (ImageJ).

### Atoh7 Genotyping

Atoh7 genotyping was performed using PCR with the following primers: (Atoh7-F: 5′-CCGGAATTACATCCCAAGAAC-3′; Atoh7-R: 5′-GGCCATGATGTAGCTCAGAG-3′) to amplify a short 293bp fragment of genomic DNA flanking the mutation site. The introduction of the Atoh7 null mutation disrupts a *StuI* restriction enzyme cut site, which was utilized to determine genotype. In addition, homozygous mutants for Atoh7 can also be distinguished morphologically due to a dramatic increase in pigmentation of the larvae.

### Electrophysiology

Whole-cell voltage-clamp recordings were made from adherent HEK293S GnTI–cells transiently transfected with the tricistronic pEZT construct of the human α1β2γ2 GABAA receptor.^42^ Upon transfection with 0.2–0.5 μg of the plasmid per well in a 12-well dish, the cells were moved to 30 °C. On the day of recording (1–3 days later), cells were replated onto a 35 mm dish and washed with bath solution, which contained (in mM): 140 NaCl, 2.4 KCl, 4 MgCl_2_, 4 CaCl2, 10 HEPES pH 7.3, and 10 glucose. Borosilicate pipettes were pulled and polished to an initial resistance of 2–4 MΩ. The pipet solution contained (in mM): 100 CsCl, 30 CsF, 10 NaCl, 10 EGTA, and 20 HEPES pH 7.3. Cells were clamped at −75 mV. The recordings were made with an Axopatch 200B amplifier, sampled at 5 kHz, and low-pass filtered at 2 kHz using a Digidata 1550B (Molecular Devices) and analyzed with pClamp 11 software (Molecular Devices). The ligand and compound solutions were prepared in bath solution with 0.06% pluronic F-68 from concentrated stocks (1 M GABA stock was prepared in water, and 30 mM compound stocks were prepared in DMSO). Solution exchange was achieved by using a gravity-driven RSC-200 rapid solution changer (Bio-Logic). Statistical analysis of the peak currents was performed by using GraphPad Prism 10.2.0 software (GraphPad Software, Inc., La Jolla, CA). Data are expressed as means ± S.D, and Welch’s *t-test* was used in Figure 4, A *p*-value of ≤0.05 was considered statistically significant (***, *p* ≤ 0.001; **, *p* ≤ 0.01 *, 0.01 ≤ *p* ≤ 0.05). Replicate numbers are labeled in Figure 4 as *n* = number of independent cells.

### Synthesis and Characterization of Isoflavones

#### General Information

All reagents were purchased from commercial suppliers (Sigma-Aldrich, Fisher Scientific, and TCI USA) and used without further purification. Analytical thin-layer chromatography (TLC) was performed on Silica Gel 60 Å F254 precoated glass plates. Plates were visualized using ultraviolet light (254 nm) or potassium permanganate stain. ^1^H NMR and ^13^C NMR traces of all synthesized compounds were acquired by using a Bruker Ascend 600 MHz spectrometer. Chemical shifts are reported relative to the residual CHCl_3_ solvent peak (7.26 ppm for the ^1^H NMR spectra and 77.00 ppm for the ^13^C NMR spectra). Multiplicities are denoted as s = singlet, d = doublet, t = triplet, q = quartet, p = pentet, and m = multiplet. High resolution mass spectra (HRMS) were recorded by Brown University staff using a Jeol JMS 600H spectrometer and an Agilent Technologies 6530 Accurate-Mass Q TOF-LC/MS. For selected compounds that underwent neural imaging, UPLC-MS to access compound purity was performed using a Waters Acquity H Class Plus featuring a PDA and ELSD detector with a QDa II mass detector.

#### General Procedure for the Synthesis of **1**

To a mixture of resorcinol (1 equiv, 20.0 mmol) and carboxylic acid (1 equiv) was added BF_3_·Et_2_O (0.4 M) in a round-bottom flask. The reaction was heated to 80 °C and stirred overnight under inert conditions. The next day the solution was allowed to cool and poured into water. The reaction mixture was extracted with ethyl acetate, and the organic layer was separated and washed with brine, dried, and concentrated. The concentrate was purified via column chromatography using ramping ethyl acetate and hexanes to obtain ketone **1**.

#### General Procedure for the Synthesis of **2**

**1** was dissolved in trifluoroacetic acid (20 equiv), and triethylsilane (2.5 equiv) was added at room temperature. The resulting solution was stirred overnight, and the solvent was removed by flushing nitrogen gas in mild temperature. The concentrate was purified via column chromatography using ramping ethyl acetate and hexanes to obtain diol **2**.

#### General Procedure for the Synthesis of **3**

To a mixture of resorcinol (1 equiv) and carboxylic acid (1 equiv) was added BF_3_·Et_2_O (0.2 M) to a round-bottom flask. The reaction was heated to 80 °C and stirred 4–16 h under inert conditions, checking completion via TLC. The next day the solution was allowed to cool and poured into water. The reaction mixture was extracted with ethyl acetate, and the organic layer was separated and washed with brine, dried and concentrated. The concentrate was purified via column chromatography using ramping ethyl acetate and hexanes to give ketone **3**.

#### General Procedure for the Synthesis of **4**

A mixture of **3**, desired anhydride (5 equiv), and triethylamine (4 equiv) was heated at 125 °C for 12 h. Then the reaction mixture was added to cold dilute 1 M HCl solution. The mixture was extracted with ethyl acetate, and the organic layer was separated and washed with brine, dried and concentrated. The concentrate was purified via column chromatography using ramping ethyl acetate and hexanes to obtain isoflavone **4**. This was the terminal differentiation step for isoflavones **(JG-3-)17, 18, 37, 38, 39, 41, 42, 43, 120, and 121***.* Occasionally, the O-acylated byproduct **5**, was also observed and subsequently converted to **4** as described below.

#### General Procedure for the Synthesis of **4** from **5**

A solution of **5** in ethanol (0.2 M) containing 10% w/w NaOH was refluxed for 30 min. After 30 min, the same amount of water was added, and heating was continued for another 1.5 h. The reaction mixture was acidified with dilute hydrochloric acid and extracted with ethyl acetate, and the organic layer was separated and washed with brine, dried, and concentrated. The concentrate was purified via column chromatography using ramping ethyl acetate and hexanes to get isoflavone **4**.

#### General Procedure for the Synthesis of **6**

To a solution of **4** in DMF (0.5 M), methyl bromoacetate (1.1 equiv) and K_2_CO_3_ (3 equiv) was added. The mixture was heated to 90 °C for 8 h. The reaction mixture was cooled to room temperature and extracted with ethyl acetate. The organic layer was separated, washed with brine, dried, and concentrated. The concentrate was purified via column chromatography using ramping ethyl acetate and hexanes to afford isoflavones **(JG-3-)44, 45, 46, 47,48 and 49**.

#### General Procedure for the Synthesis of **7**

Isoflavone **4** was dissolved in a minimum amount of pyridine. Then, the desired acyl chloride (propionyl chloride or butyryl chloride) was added, and the reaction mixture was stirred at room temperature for 24h. Reaction was poured into cold water and extracted with ethyl acetate. The organic layer was separated, washed with brine, dried and concentrated. The concentrate was purified via column chromatography using ramping ethyl acetate and hexanes to afford isoflavones **(JG-3)13, 16, 29, 30, 31, 34, 35, and 119**.

#### Synthesis of **JG3-138** from **JG3-018**

Methyl was done by mixing **JG3-018** with MeI with DMF. K_2_CO_3_ was added as inorganic base. Reaction mixture was mixed at 80 °C in a microwave vial overnight. Reaction mixture was cooled to room temperature and washed with ethyl acetate multiple times. Organic layer was dried in vacuo and purified with column chromatography using ramping ethyl acetate and hexanes.

#### General Procedure for the Synthesis of **8**

To a mixture of desired naphthol (1 equiv) and 2-chlorophenylacetic acid (1 equiv) was added BF_3_·Et_2_O (0.2 M) in a round-bottom flask. The reaction was heated to 80 °C and stirred overnight under inert conditions, checking completion via TLC. The next day if the product had crashed out of solution, it was filtered, washed with water, and used without further purfication. the solution was allowed to cool and poured into water. If product did not precipitate, the reaction mixture alllowed to cool and poured into water. It was then extracted with ethyl acetate, and the organic layer was separated and washed with brine, dried and concentrated. The concentrate was purified via column chromatography using ramping ethyl acetate and hexanes to give ketone **8**.

#### General Procedure for the Synthesis of **9**

A mixture of **8**, propionic anhydride (5 equiv), and triethylamine (4 equiv) was heated at 125 °C for 12 h. Then the reaction mixture was added to cold dilute 1 M HCl solution. The mixture was extracted with ethyl acetate, and the organic layer was separated and washed with brine, dried and concentrated. The concentrate was purified via column chromatography using ramping ethyl acetate and hexanes to obtain isoflavone **9**.

#### 2-Ethyl-4-oxo-6-propyl-3-(3-(trifluoromethoxy)phenyl)-4H-chromen-7-yl propionate (JG-3-013)

^1^H NMR (600 MHz, chloroform-d) δ = 8.08 (s, 1 H), 7.47–7.45 (m, 1H), 7.27 (s, 1 H), 7.25–7.23 (d, 1 H), 7.23–7.21 (d, 1 H), 7.15 (s, 1 H), 7.27–7.27 (m, 2 H), 2.61–2.59 (m, 2 H), 2.57–2.55 (m, 2 H), 1.67–1.61 (m, 2 H), 1.35–1.32 (m, 3 H), 1.27–1.24 (m, 3 H), 0.97–0.94 (m, 3 H); ^13^C NMR (600 MHz, chloroform-d) δ = 176.31, 172.39, 167.95, 154.80, 153.18, 149.37, 135.19, 132.67, 129.91, 129.13, 127.43, 123.21, 121.87, 121.24, 120.42, 111.49, 32.09, 27.97, 26.32, 23.22, 14.00, 12.00, 9.29; HRMS (ESI) *m*/*z* [M + H]^+^ calculated for C_24_H_24_F_3_O_5_ 449.1476; found, 449.1568.

#### 3-(2-Chlorophenyl)-2-ethyl-4-oxo-6-propyl-4H-chromen-7-yl propionate (JG-3-016)

^1^H NMR (600 MHz, chloroform-d) δ = 8.09 (s, 1 H), 7.51–7.49 (m, 1 H), 7.36–7.31 (m, 2 H), 7.26 (s, 1 H), 7.23–7.22 (m, 1 H), 2.70–2.66 (m, 2 H), 2.60–2.59 (m, 2 H), 2.53–2.40 (m, 2 H), 1.67–1.61 (m, 2 H), 1.34–1.32 (m, 3 H), 1.23–1.20 (m, 3 H), 0.96–0.94 (m, 3 H); ^13^C NMR (600 MHz, chloroform-d) δ = 175.95, 172.44, 168.20, 154.93, 153.06, 135.00, 132.54, 132.31, 129.89, 129.72, 127.53, 127.11, 121.29, 121.00, 111.50, 32.12, 27.99, 26.35, 23.25, 14.06, 11.43, 9.33; HRMS (ESI) *m*/*z* [M + H]^+^ calculated for C_23_H_24_ClO_4_ 399.1363; found, 399.1349.

#### 2-Ethyl-7-hydroxy-6-propyl-3-(3-(trifluoromethoxy)phenyl)-4H-chromen-4-one (JG-3-017)

^1^H NMR (600 MHz, chloroform-d) δ = 7.94 (s, 1 H0, 7.42–7.39 (m, 1 H), 7.23–7.22 (d, 1 H), 7.17–7.11 (m, 2 H), 6.78 (m, 1 H), 3.66 (s, 1 H), 2.63–2.60 (m, 2 H), 2.56–2.52 (m, 2 H), 1.66–1.62 (m, 2 H), 1.25–1.22 (m, 3 H), 0.96–0.94 (m, 3 H); ^13^C NMR (600 MHz, chloroform-d) δ = 177.38, 167.95, 160.40, 156.44, 135.35, 130.00, 129.20, 129.01, 126.84, 123.22, 122.22, 121.35, 120.35, 119.93, 116.24, 102.25, 31.82, 26.33, 22.68, 14.05, 12.04; HRMS (ESI) *m*/*z* [M + H]^+^ calculated for C_21_H_20_F_3_O_4_ 393.1314; found, 393.1311.

#### 3-(2-Chlorophenyl)-2-ethyl-7-hydroxy-6-propyl-4H-chromen-4-one (JG-3-018)

^1^H NMR (600 MHz, chloroform-d) δ = 8.57 (s, 1 H), 7.94 (s, 1 H), 7.43–7.41 (d, 1 H), 7.29–7.24 (m, 3 H), 6.80 (s, 1 H), 2.62–2.59 (m, 2 H), 2.52–2.39 (m, 2 H), 1.67–1.61 (m, 2 H), 1.23–1.20 (m, 3 H), 0.96–0.94 (m, 3 H); ^13^C NMR (600 MHz, chloroform-d) δ = 177.09, 168.02, 160.08, 156.56, 135.08, 132.67, 132.39, 129.75, 129.60, 129.11, 127.04, 126.57, 120.23, 115.91, 102.28, 31.89, 26.33, 22.68, 14.12, 11.48; HRMS (ESI) *m*/*z* [M + H]^+^ calculated for C_20_H_20_ClO_3_ 343.1101; found, 343.1103.

#### 3-(2-Chlorophenyl)-4-oxo-2,6-dipropyl-4H-chromen-7-yl butyrate (JG-3-029)

^1^H NMR (600 MHz, chloroform-d) δ = 8.09 (s, 1 H), 7.50–7.48 (m, 1 H), 7.35–7.31 (m, 2 H), 7.24 (s, 1 H), 7.23–7.21 (m, 1 H), 2.63–2.61 (m, 2 H), 2.60–2.58 (m, 2 H), 2.49–2.35 (m, 2 H), 1.86–1.81 (m, 2 H), 1.73- 1.68 (m, 2 H), 1.67–1.61 (m, 2 H), 1.10–1.08 (m, 3 H), 0.97–0.94 (m, 3 H), 0.91–0.88 (m, 3 H); ^13^CNMR (600 MHz, chloroform-d) δ = 175.88, 171.64, 167.24, 154.87, 153.06, 135.03, 132.56, 132.41, 129.86, 129.70, 127.51, 127.05, 121.62, 121.25, 111.48, 36.40, 34.64, 32.13, 23.27, 20.39, 18.63, 14.07, 13.96, 13.88; HRMS (ESI) *m*/*z* [M + H]^+^ calculated for C_25_H_28_ClO_4_ 427.1676; found, 427.1698.

#### 3-(2-Chlorophenyl)-2-ethyl-4-oxo-6-phenethyl-4H-chromen-7-yl propionate (JG-3-030)

^1^H NMR (600 MHz, chloroform-d) δ = 8.14 (s, 1 H), 7.52–7.50 (m, 1H), 7.36–7.34 (m, 2 H), 7.31–7.28 (m, 3 H), 7.24–7.18 (m, 4 H), 2.92 (m, 4 H), 2.68–2.64 (m, 2 H), 2.55–2.41 (m, 2 H), 1.33–1.30 (m, 3 H), 1.24–1.21 (m, 3 H); ^13^C NMR (600 MHz, chloroform-d) δ = 175.86, 172.33, 168.28, 155.06, 152.97, 141.30, 134.99, 132.48, 132.39, 131.72, 129.89, 129.76, 128.68, 128.49, 127.56, 127.13, 126.37, 121.33, 121.03, 111.56, 36.59, 32.18, 27.92, 26.36, 11.43, 9.28; HRMS (ESI) *m*/*z* [M + H]^+^ calculated for C_28_H_26_ClO_4_ 461.1520; found, 461.1531.

#### 3-(2-Chlorophenyl)-4-oxo-6-phenethyl-2-propyl-4H-chromen-7-yl butyrate (JG-3-031)

^1^H NMR (600 MHz, chloroform-d) δ = 8.15 (s, 1 H), 7.52–7.50 (m, 1 H), 7.35–7.34 (m, 2 H), 7.31–7.28 (m, 3 H), 7.24–7.19 (m, 4 H), 2.92–2.91 (m, 4 H), 2.63–2.60 (m, 2 H), 2.52–2.36 (m, 2 H), 1.86–1.77 (m, 2 H), 1.76–1.65 (m, 2 H), 1.09–1.07 (m, 3 H), 0.92–0.90 (m, 3 H); ^13^C NMR (600 MHz, chloroform-d) δ = 175.79, 171.53, 167.34, 155.01, 152.98, 141.31, 135.02, 132.50, 132.39, 131.17, 129.88, 129.73, 128.48, 127.56, 127.07, 126.36, 121.66, 121.31, 111.55, 36.55, 34.65, 32.18, 20.40, 18.59, 13.98, 13.88; HRMS (ESI) *m*/*z* [M + H]^+^ calculated for C_30_H_30_ClO_4_ 489.1833; found, 489.1849.

#### 2-Ethyl-4-oxo-6-phenethyl-3-(3-(trifluoromethoxy)phenyl)-4H-chromen-7-yl propionate (JG-3-034)

^1^HNMR (600 MHz, chloroform-d) δ = 8.11 (s, 1 H), 7.48–7.46 (m, 1 H), 7.30–7.26 (m, 3 H), 7.23–7.21 (m, 3 H), 7.18–7.15 (m, 3 H), 2.92 (m, 4 H), 2.68–2.64 (m, 2 H), 2.59–2.55 (m, 2 H), 1.32- 1.30 (m, 3 H), 1.27–1.24 (m, 3 H); ^13^C NMR (600 MHz, chloroform-d) δ = 176.22, 172.29, 168.02, 154.49, 153.10, 149.38, 141.21, 135.13, 131.83, 129.95, 129.13, 128.70, 128.51, 127.51, 126.40, 123.21, 121.91, 121.30, 120.47, 111.55, 36.56, 32.15, 27.93, 26.35, 12.01, 9.27; HRMS (ESI) *m*/*z* [M + H]^+^ calculated for C_29_H_26_F_3_O_5_ 511.1732; found, 511.1745.

#### 4-Oxo-6-phenethyl-2-propyl-3-(3-(trifluoromethoxy)phenyl)-4H-chromen-7-yl butyrate (JG-3-035)

^1^H NMR (600 MHz, chloroform-d) δ = 8.11 (s, 1 H), 7.48–7.46 (m, 1 H), 7.30–7.28 (m, 2 H), 7.27 (s, 1 H), 7.25–7.24 (d, 1 H), 7.22–7.21 (d, 2 H), 7.18–7.17 (d, 2 H), 7.14 (s, 1 H), 2.92 (m, 4 H), 2.62–2.60 (m, 2 H), 2.53–2.51 (m, 2 H), 1.84–1.81 (m, 2 H), 1.75–1.71 (m, 2 H), 1.09–1.06 (m, 3 H), 0.93–0.90 (m, 3 H); ^13^C NMR (600 MHz, chloroform-d) δ = 176.19, 171.49, 167.01, 154.89, 153.11, 149.36, 141.21, 131.84, 129.92, 128.68, 128.50, 127.50, 126.39, 123.31, 122.56, 121.28, 120.48, 111.55, 46.52, 36.34, 34.57, 32.15, 20.98, 18.58, 13.89, 13.85; HRMS (ESI) *m*/*z* [M + H]^+^ calculated for C_31_H_30_F_3_O_5_ 539.2045; found, 539.2066.

#### 3-(2-Chlorophenyl)-7-hydroxy-2,6-dipropyl-4H-chromen-4-one (JG-3-037)

^1^H NMR (600 MHz, chloroform-d) δ = 8.76 (s, 1H), 7.94, (s, 1H), 7.42–7.40 (d, 1H), 7.27–7.24 (m, 3H), 6.80 (s, 1H), 2.61–2.59 (m, 2H), 2.50–2.35 (m, 2H), 1.75–1.68 (m, 2H), 1.68–1.60 (m, 2H), 0.96–0.94 (m, 3H), 0.91 (m, 3H); ^13^C NMR (600 MHz, chloroform-d) δ = 177.16, 167.20, 160.99, 156.60, 135.17, 132.71, 132.55, 129.77, 129.61, 129.26, 127.03, 126.53, 120.89, 115.83, 102.33, 34.67, 31.96, 22.70, 20.48, 14.17, 13.98; HRMS (ESI) *m*/*z* [M + H]^+^ calculated for C_21_H_22_ClO_3_ 357.1257; found, 357.1243.

#### 3-(2-Chlorophenyl)-2-ethyl-7-hydroxy-6-phenethyl-4H-chromen-4-one (JG-3-038)

^1^H NMR (600 MHz, chloroform-d) δ = 8.01 (s, 1 H), 7.45–7.43 (d, 1 H), 7.29–7.24 (m, 6 H), 7.22–7.19 (m, 2 H), 6.82 (s, 1 H), 2.94–2.93 (m, 4 H), 2.51–2.40 (m, 2 H), 1.25–1.20 (m, 3 H); ^13^C NMR (600 MHz, chloroform-d) δ = 176.78, 168.03, 160.12, 156.61, 141.95, 135.10, 132.64, 132.39, 129.84, 129.71, 128.67, 128.60, 127.12, 127.07, 126.24, 120.47, 116.55, 102.63, 36.17, 32.32, 26.37, 11.54; HRMS (ESI) *m*/*z* [M + H]^+^ calculated for C_25_H_22_ClO_3_ 405.1257; found, 405.1255.

#### 3-(2-Chlorophenyl)-7-hydroxy-6-phenethyl-2-propyl-4H-chromen-4-one (JG-3-039)

^1^H NMR (600 MHz, chloroform-d) δ = 8.80 (s, 1 H), 8.01 (s, 1 H), 7.41–7.40 (d, 1 H), 7.26–7.25 (m, 4 H), 7.22–7.21 (m, 3 H), 7.19–7.16 (m, 1 H), 6.83 (s, 1 H), 2.94–2.91 (m, 4 H), 2.50–2.35 (m, 2 H), 1.75–1.66 (m, 2 H), 0.91–0.88 (m, 3 H); ^13^C NMR (600 MHz, chloroform-d) δ = 177.14, 167.35, 161.06, 156.77, 142.15, 135.19, 132.63, 132.53, 129.08, 129.68, 128,68, 128,59, 127.06, 126.62, 126.11, 10.92, 115.91, 102.51, 36.07, 34.69, 32.44, 20.51, 13.98; HRMS (ESI) *m*/*z* [M + H]^+^ calculated for C_26_H_24_ClO_3_ 419.1414; found, 419.1402.

#### 7-Hydroxy-2,6-dipropyl-3-(3-(trifluoromethoxy)phenyl)-4H-chromen-4-one (JG-3-041)

^1^H NMR (600 MHz, chloroform-d) δ = 8.28 (s, 1 H), 7.94 (s, 1 H), 7.42–7.39 (m, 1 H), 7.23–7.22 (m, 1 H), 7.17–7.15 (m, 2 H), 6.77 (s, 1 H), 2.63–2.60 (m, 2 H), 2.51–2.48 (m, 2 H), 1.74–1.66 (m, 2 H), 1.66–1.61 (m, 2 H), 0.96–0.94 (m, 3 H), 0.91–0.90 (m, 3 H); ^13^C NMR (600 MHz, chloroform-d) δ = 177.40, 166.01, 160.67, 156.44, 149.38, 135.47, 129.91, 129.33, 129.19, 126.77, 123.34, 121.96, 120.33, 116.08, 102.20, 34.57, 31.86, 22.67, 21.04, 14.06, 13.80; HRMS (ESI) *m*/*z* [M + H]^+^ calculated for C_22_H_22_F_3_O_4_ 407.1470; found, 407.1482.

#### 2-Ethyl-7-hydroxy-6-phenethyl-3-(3-(trifluoromethoxy)phenyl)-4H-chromen-4-one (JG-3-042)

^1^H NMR (600 MHz, chloroform-d) δ = 7.98 (s, 1 H), 7.28–7.25 (m, 3 H), 7.23–7.22 (d, 2 H), 7.19–7.16 (m,4 H), 6.79 (s, 1 H), 3.67 (s, 2 H), 2.95–2.91 (m, 4 H), 2.55–2.51 (m, 2 H), 1.24–1.22 (m, 3 H); ^13^CNMR (600 MHz, chloroform-d) δ = 177.24, 175.89, 170.00, 160.29, 156.53, 141.79, 130.10, 129.97, 129.73, 129.21, 128.65, 128.58, 120.01, 127.03, 126.26, 123.24, 122.22, 121.41, 120.40, 119.97, 102.49, 40.65, 36.06, 32.17, 26.33, 12.04; HRMS (ESI) *m*/*z* [M + H]^+^ calculated for C_26_H_22_F_3_O_4_ 455.1470; found, 455.1460

#### 7-Hydroxy-6-phenethyl-2-propyl-3-(3-(trifluoromethoxy)phenyl)-4H-chromen-4-one (JG-3-043)

^1^H NMR (600 MHz, chloroform-d) δ = 8.00 (s, 1 H), 7.41–7.39 (m, 1 H), 7.26–7.22 (m, 3 H), 7.19–7.14 (m, 6 H), 6.79 (s, 1 H), 2.95–2.90 (m, 4 H), 2.50–2.48 (m, 2 H), 1.74–1.67 (m, 2 H), 0.91–0.89 (m, 3 H); ^13^C NMR (600 MHz, chloroform-d) δ = 177.53, 167.20, 160.95, 156.65, 149.39, 141.90, 135.46, 129.98, 129.33, 128.58, 126.72, 126.16, 123.34, 121.94, 120.41, 116.02, 102.36, 35.96, 34.59, 32.22, 21.05, 13.80; HRMS (ESI) *m*/*z* [M + H]^+^ calculated for C_27_H_24_F_3_O_4_ 469.1627; found, 469.1593.

#### Methyl 2-((3-(2-Chlorophenyl)-2-ethyl-4-oxo-6-propyl-4H-chromen-7-yl)oxy)acetate (JG-3-044)

^1^H NMR (600 MHz, chloroform-d) δ = 7.98 (s, 1 H), 7.49–7.48 (m, 1 H), 7.33–7.31 (m, 2 H), 7.23–7.21 (m, 1 H), 6.99 (s, 1 H), 4.77 (s, 1 H), 3.84 (s, 1 H), 2.73–2.71 (m, 2 H0, 2.51–2.38 (m, 2 H), 1.71–1.65 (m, 2 H), 1.23–1.20 (m, 3 H), 0.98–0.95 (m, 3 H); ^13^C NMR (600 MHz, chloroform-d) δ = 175.87, 168.72, 167.37, 160.28, 156.20, 134.99, 132.77, 132.34, 130.30, 129.81, 129.60, 127.10, 127.04, 120.91, 117.50, 98.83, 65.46, 52.63, 32.08, 29.86, 26.26, 22.87, 14.12, 11.54; HRMS (ESI) *m*/*z* [M + H]^+^ calculated for C_23_H_24_ClO_5_ 415.1312; found, 415.1306.

#### Methyl 2-((3-(2-Chlorophenyl)-4-oxo-2,6-dipropyl-4H-chromen-7-yl)oxy)acetate (JG-3-045)

^1^H NMR (600 MHz, chloroform-d) δ = 7.99 (s, 1 H), 7.49–7.48 (m, 1 H), 7.33–7.31 (m, 2 H), 7.22–7.21 (m, 1 H), 6.69 (s, 1 H), 4.77 (s. Two H), 3.85 (s, 3 H), 2.73–2.71 (m, 2 H), 2.49–2.33 (m, 2 H), 1.72–1.66 (m, 4 H), 0.98–0.95 (m, 3 H), 0.91–0.88 (m, 3 H); ^13^C NMR (600 MHz, chloroform-d) δ = 175.80, 168.74, 166.40, 160.28, 156.17, 135.04, 132.81, 132.46, 130.31, 129.83, 129.59, 127.13, 127.00, 121.58, 117.53, 98.83, 65.49, 52.65, 34.61, 32.10, 39.88, 22.91, 20.51, 14.16, 13.99; HRMS (ESI) *m*/*z* [M + H]^+^ calculated for C_24_H_26_ClO_5_ 429.1469; found, 429.1467.

#### Methyl 2-((3-(2-Chlorophenyl)-2-ethyl-4-oxo-6-phenethyl-4H-chromen-7-yl)oxy)acetate (JG-3-046)

^1^H NMR (600 MHz, chloroform-d) δ = 8.03 (s, 1 H), 7.50–7.49 (m, 1 H), 7.34–7.33 (m, 2 H), 7.30–7.27 (m, 2 H), 7.26–7.24 (m, 3 H), 7.20–7.18 (m, 1 H), 6.72 (s, 1 H), 4.74 (s, 2 H), 3.86 (s, 3 H), 3.06–2.94 (m, 4 H), 2.53–2.39 (m, 2 H), 1.24–1.21 (m, 3 H); ^13^C NMR (600 MHz, chloroform-d) δ = 175.81, 168.61, 167.46, 160.25, 156.40, 142.09, 135.02, 132.74, 132.35, 129.87, 129.66, 128.70, 128.50, 127.27, 127.09, 126.10, 121.00, 117.66, 99.05, 65.55, 52.68, 36.31, 32.51, 26.30, 11.57; HRMS (ESI) *m*/*z* [M + H]^+^ calculated for C_28_H_26_ClO_5_ 477.1469; found, 477.1470.

#### Methyl 2-((3-(2-Chlorophenyl)-4-oxo-6-phenethyl-2-propyl-4H-chromen-7-yl)oxy)acetate (JG-3-047)

^1^H NMR (600 MHz, chloroform-d) δ = 8.06 (s, 1 H0, 7.53–7.51 (d, 1 H), 7.36–7.35 (m, 2 H), 7.33–7.25 (m, 5 H), 7.23–7.20 (m, 1 H), 6.74 (s, 1 H), 4.76 (s, 2 H0, 3.89 (s, 3 H), 3.09–2.97 (m, 4 H), 2.53–2.37 (m, 2 H), 1.77–1.70 (m, 2 H), 0.94–0.92 (m, 3 H); ^13^C NMR (600 MHz, chloroform-d) δ = 175.70, 168.56, 166.46, 160.20, 156.32, 142.05, 135.01, 132.71, 132.41, 129.82, 129.60, 129.52, 128.66, 128.46, 127.20, 127.00, 126.06, 121.59, 117.60, 99.00, 65.50, 52.63, 36.27, 34.59, 32.49, 20.48, 13.97; HRMS (ESI) *m*/*z* [M + H]^+^ calculated for C_29_H_28_ClO_5_ 491.1625; found, 491.1632.

#### Methyl 2-((2-Ethyl-4-oxo-6-phenethyl-3-(3-(trifluoromethoxy)phenyl)-4H-chromen-7-yl)oxy)acetate (JG-3-048)

^1^H NMR (600 MHz, chloroform-d) δ = 8.02 (s, 1 H), 7.50–7.47 (m, 1 H), 7.32–7.29 (m, 3 H), 7.27–7.20 (m, 4 H), 7.17 (s, 1 H), 6.72 (s, 1 H), 4.75 (s, 2 H), 3.99 (s, 3 H), 3.09–2.96 (m, 4 H), 2.60–2.56 (m, 2 H), 1.30–1.27 (m, 3 H); ^13^C NMR (600 MHz, chloroform-d) δ = 176.12, 168.55, 167.20, 160.36, 156.27, 149.36, 141.97, 135.38, 129.87, 129.68, 129.18, 128.71, 128.50, 127.21, 126.12, 123.23, 121.88, 120.36, 117.63, 98.98, 65.56, 52.68, 36.27, 32.44, 26.26, 12.14; HRMS (ESI) *m*/*z* [M + H]^+^ calculated for C_29_H_26_F_3_O_6_ 527.1681; found, 527.1692.

#### Methyl 2-((4-Oxo-6-phenethyl-2-propyl-3-(3-(trifluoromethoxy)phenyl)-4H-chromen-7-yl)oxy)acetate (JG-3-049)

^1^H NMR (600 MHz, chloroform-d) δ = 8.03 (s, 1 H), 7.50–7.47 (m, 1 H), 7.32–7.20 (m, 7 H), 7.16 (s, 1 H), 6.71 (s, 1 H), 4.75 (s, 2 H), 3.89 (s, 3 H), 3.09–3.07 (2.97 (m, 4 H), 2.54–2.52 (m, 2 H), 1.79–1.72 (m, 2 H), 0.96–0.93 (m, 3 H); ^13^C NMR (600 MHz, chloroform-d) δ = 176.09, 168.54, 166.16, 160.35, 156.23, 149.33, 141.98, 135.45, 129.85, 129.68, 129.30, 128.70, 128.50, 127.20, 126.11, 123.33, 122.52, 120.37, 117.63, 98.98, 65.55, 52.67, 36.27, 34.53, 32.50, 29.89, 21.09, 13.87; HRMS (ESI) *m*/*z* [M + H]^+^ calculated for C_30_H_28_F_3_O_6_ 541.1838; found, 541.1815.

#### 3-(3,5-Dimethoxyphenyl)-2-ethyl-4-oxo-6-propyl-4H-chromen-7-yl propionate (JG-3-119)

^1^H NMR (600 MHz, chloroform-d) δ = 8.07 (s, 1 H), 7.23 (s, 1 H), 6.47 (s, 1 H), 6.40 (s, 2 H), 3.79 (s, 6 H), 2.67- 2.65 (m, 2 H), 2.60–2.55 (m, 4 H), 1.65–1.60 (m, 2 H), 1.33–1.30 (m, 3 H), 1.24–1.22 (t, 3 H), 0.96–0.93 (t, 3 H); ^13^C NMR (600 MHz, chloroform-d) δ = 176.47, 172.31, 167.61, 160.82, 154.70, 152.89, 135.12, 132.27, 127.35, 123.02, 121.29, 111.33, 108.40, 100.20, 55.50, 32.00, 27.86, 26.22, 23.17, 13.93, 12.11, 9.21; HRMS (ESI) *m*/*z* [M + H]^+^ calculated for C_25_H_29_O_6_ 425.1964; found, 425.1966.

#### 3-(2,4-Dichlorophenyl)-2-ethyl-7-hydroxy-6-propyl-4H-chromen-4-one (JG-3-120)

^1^H NMR (600 MHz, dimethyl sulfoxide-d6) δ = 10.84 (s, 1 H), 7.74 (s, 1 H), 7.69 (s, 1 H), 7.51–7.49 (d, 1 H), 7.37–7.36 (d, 1 H), 6.90 (s, 1 H), 2.60–2.58 (t, 2 H), 2.42–2.32 (m, 2 H), 1.61–1.55 (m, 2 H), 1.14–1.12 (t, 3 H), 0.95–0.93 (t, 3 H); ^13^C NMR (600 MHz, dimethyl sulfoxide-d6) δ = 174.10, 166.54, 160.81, 155.59, 135.07, 133.79, 133.48, 131.87, 128.76, 128.37, 127.47, 125.67, 118.69, 114.80, 101.42, 31.20, 25.57, 22.19, 13.71, 11.15; HRMS (ESI) *m*/*z* [M + H]^+^ calculated for C_20_H_19_Cl_2_O_3_ 377.0711; found, 377.0701.

#### 3-(3,5-Dimethoxyphenyl)-2-ethyl-7-hydroxy-6-propyl-4H-chromen-4-one (JG-3-121)

^1^H NMR (600 MHz, dimethyl sulfoxide-d6) δ = 10.75 (s, 1 H), 7.69 (s, 1 H), 6.86 (s, 1 H), 6.50 (s, 1 H), 6.37 (s, 1 H), 3.76 (s, 6 H), 2.60–2.58 (t, 2 H), 2.48–2.45 (t, 2 H), 1.61–1.54 (m, 2 H), 1.18–1.150 (3, 3 H), 0.91–0.88 (t, 3 H); ^13^C NMR (600 MHz, dimethyl sulfoxide-d6) δ = 174.87, 165.89, 160.58, 160.17, 155.44, 135.55, 128.01, 125.73, 121.70, 115.15, 108.45, 101.29, 99.21, 55.15, 31.23, 25.52, 22.18, 13.67, 11.80; HRMS (ESI) *m*/*z* [M + H]^+^ calculated for C_22_H_25_O_5_ 369.1702; found, 369.1672.

#### 2-(2-Chlorophenyl)-3-ethyl-1H-benzo[f]chromen-1-one (JG-3-127)

^1^H NMR (600 MHz, chloroform-d) δ = 10.07–10.60 (d, 1 H), 8.11–8.10 (d, 1 H), 7.92–7.91 (d, 1 H), 7.73–7.70 (t, 1 H), 7.62–7.60 (t, 1 H), 7.57–7.54 (t, 1 H), 7.39–7.38 (m, 2 H), 7.33–7.32 (t, 1 H), 2.58–2.68 (m, 2 H), 1.32–1.29 (t, 3 H); ^13^C NMR (600 MHz, chloroform-d) δ = 178.22, 165.39, 157.42, 135.39, 134.91, 132.81, 132.28, 130.86, 130.69, 129.82, 129.29, 128.23, 127.33, 127.10, 126.58, 123.47, 117.58, 116.58, 28.83, 25.93, 11.46, 8.51; HRMS (ESI) *m*/*z* [M + H]^+^ calculated for C_21_H_16_ClO_2_ 335.0839; found, 335.0843.

#### 3-(2-Chlorophenyl)-2-ethyl-7-methoxy-6-propyl-4H-chromen-4-one (JG-3-138)

^1^H NMR (600 MHz,chloroform-d) δ = 7.95 (s, 1 H), 7.50–7.48 (m, 1 H), 7.35–7.30 (m, 1 H), 7.25–7.22 (m, 1 H), 6.84v(s, 1 H), 3.94 (s, 3 H), 2.67–2.63 (t, 2 H), 2.54–2.37 (m, 2 H), 1.68–1.59 (m, 2 H), 1.25–1.21 (t, 3H), 0.93–0.90 (t, 3 H); ^13^C NMR (600 MHz, chloroform-d) δ = 176.02, 167.17, 162.37, 156.64, 135.05, 132.97, 132.42, 130.06, 129.83, 129.57, 127.05, 126.49, 120.83, 116.69, 98.11, 56.02, 32.05, 26.30, 22.92, 14.19, 11.58; HRMS (ESI) *m*/*z* [M + H]^+^ calculated for C_21_H_22_ClO_3_ 357.1257; found, 357.1260.

#### 8-Bromo-2-(2-chlorophenyl)-3-ethyl-1H-benzo[f]chromen-1-one (JG-3-139)

^1^H NMR (600 MHz, chloroform-d) δ = 9.92–9.93 (d, 1 H), 8.03–9.02 (d, 1 H), 7.98–7.97 (d, 1 H), 7.75–7.74 (d, 1 H), 7.57–7.55 (d, 1 H), 7.53–7.51 (d, 1 H), 7.38–7.34 (m, 2 H), 7.30–7.27 (t, 1 H), 2.61–2.43 (m, 2 H), 1.28–1.26 (t, 3 H); ^13^C NMR (600 MHz, chloform-d) δ = 177.93, 165.75, 157.30, 134.89, 134.20, 132.56, 132.37, 132.22, 132.10, 130.21, 129.89, 129.79, 129.44, 129.20, 127.17, 123.60, 120.77, 118.94, 116.69, 29.84, 23.98, 11.44; HRMS (ESI) *m*/*z* [M + H]^+^ calculated for C_21_H_15_BrClO_2_ 412.9944; found, 412.9939.

#### 3-(2-Chlorophenyl)-2-ethyl-4H-benzo[h]chromen-4-one (JG-3-154)

^1^H NMR (600 MHz, chloroform-d) δ = 8.54–8.53 (d, 1 H), 8.20–8.19 (d, 1 H), 8.00–7.94 (d, 1 H), 7.78–7.77 (d, 1 H), 7.73–7.70 (t, 1 H), 7.69–7.68 (m, 2 H), 7.54–7.53 (d, 1 H), 7.38–36 (m, 2 H), 7.36–7.29 (t, 1 H), 2.73–2.59 (m, 2 H), 1.40–1.37 (t, 3 H); ^13^C NMR (600 MHz, chloroform-d) δ = 176.30, 167.01, 153.53, 135.98, 134.90, 132.48, 132.26, 129.88, 129.75, 128.31, 127.16, 127.12, 125.12, 124.14, 122.29, 122.27, 121.56, 119.71, 26.31, 11.68; HRMS (ESI) *m*/*z* [M + H]^+^ calculated for C_21_H_16_ClO_2_ 335.0839; found, 335.0815.

#### 2-(2-Chlorophenyl)-3-ethyl-1-oxo-1H-benzo[f]chromen-6-yl propionate (JG-3-155)

^1^H NMR (600 MHz, chloroform-d) δ = 10.10–10.09 (d, 1 H), 8.04–8.03 (d, 1 H), 7.75–7.72 (t, 1 H), 7.64–7.61 (t, 1 H), 7.55–7.53 (t, 1 H), 7.49 (s, 1 H), 7.38–7.37 (m, 2 H), 7.31–7.30 (t, 1 H), 2.86–2.84 (m, 2), 2.61–2.47 (m, 2 H), 1.43–1.1.40 (t, 3 H), 1.29–1.26 (t, 3 H); ^13^C NMR (600 MHz, chloroform-d) δ = 177.55, 172.18, 165.54, 157.12, 151.19, 134.94, 132.69, 132.27, 132.05, 129.92, 129.85, 129.70, 127.64, 127.11, 126.87, 124.74, 123.52, 121.25, 114.93, 109.81, 28.07, 25.90, 11.41, 9.28; HRMS (ESI) *m*/*z* [M + H]^+^ calculated for C_24_H_20_ClO_4_ 407.1050; found, 407.1033.

#### 2-(2-Chlorophenyl)-3-ethyl-6-hydroxy-1H-benzo[f]chromen-1-one (JG-3-156)

^1^H NMR (600 MHz, dimethyl sulfoxide-d6) δ = 9.85–9.84 (d, 1 H), 8.38–8.37 (d, 1 H), 7.70–7.68 (t, 1 H), 7.60–7.56 (m, 2 H), 7.44–7.41 (m, 2 H), 7.34–7.33 (d, 1 H), 6.90 (s, 1 H), 2.56–2.44 (m, 2 H), 1.27–1.24 (t, 3 H); ^13^C NMR (600 MHz, dimethyl sulfoxide-d6) δ = 179.23, 166.69, 161.46, 161.21, 136.19, 134.32, 133.71, 133.24, 130.97, 130.77, 130.59, 128.37, 127.76, 126.88, 126.25, 123.95, 123.74, 110.91, 98.77, 10.84, 26.77, 11.66; HRMS (ESI) *m*/*z* [M + H]^+^ calculated for C_21_H_16_ClO_3_ 351.0788; found, 351.0402.

## Data Availability

All motion index time series data used in this study is available upon request.

## Supporting Information Available

Full dose–response effects of analogs, chemical structures of isoflavones, extended behavioral data, and genotyping results for *Atoh* mutants. Also included are Supplementary Tables with chemical names, SMILES, SAR information, neuroanatomical data from pERK studies, and Spectral Analysis of isoflavones (PDF)

## Accession Codes

All code is available upon request.

## Abbreviations

CNS: central nervous system
dpf: days post fertilization
eASR: enhanced acoustic startle response
GABA: gamma-aminobutyric acid
SAR: structure activity relationship
TSPO: translocator protein
PDSP: psychoactive drug screening program
HEK: human embryonic kidney
FLIPR: fluorometric imaging plate reader
PAM: positive allosteric modulator
RGC: retinal ganglion cells

## References

[ref1] McCarroll M. N.; Gendelev L.; Keiser M. J.; Kokel D. Leveraging Large-Scale Behavioral Profiling in Zebrafish to Explore Neuroactive Polypharmacology. ACS Chem. Biol. 2016, 11 (4), 842–849. 10.1021/acschembio.5b00800. 26845413 PMC5067259

[ref2] McCarroll M. N.; Gendelev L.; Kinser R.; Taylor J.; Bruni G.; Myers-Turnbull D.; Helsell C.; Carbajal A.; Rinaldi C.; Kang H. J.; Gong J. H.; Sello J. K.; Tomita S.; Peterson R. T.; Keiser M. J.; Kokel D. Zebrafish Behavioural Profiling Identifies GABA and Serotonin Receptor Ligands Related to Sedation and Paradoxical Excitation. Nat. Commun. 2019, 10 (1), 1–14. 10.1038/s41467-019-11936-w. 31501447 PMC6733874

[ref3] Kokel D.; Bryan J.; Laggner C.; White R.; Cheung C. Y. J.; Mateus R.; Healey D.; Kim S.; Werdich A. A.; Haggarty S. J.; MacRae C. A.; Shoichet B.; Peterson R. T. Rapid Behavior—Based Identification of Neuroactive Small Molecules in the Zebrafish. Nat. Chem. Biol. 2010, 6 (3), 231–237. 10.1038/nchembio.307. 20081854 PMC2834185

[ref4] Myers-Turnbull D.; Taylor J. C.; Helsell C.; McCarroll M. N.; Ki C. S.; Tummino T. A.; Ravikumar S.; Kinser R.; Gendelev L.; Alexander R.; Keiser M. J.; Kokel D. Simultaneous Analysis of Neuroactive Compounds in Zebrafish. bioRxiv 2022, 10.1101/2020.01.01.891432.

[ref5] Randlett O.; Wee C. L.; Naumann E. A.; Nnaemeka O.; Schoppik D.; Fitzgerald J. E.; Portugues R.; Lacoste A. M. B.; Riegler C.; Engert F.; Schier A. F. Whole-Brain Activity Mapping onto a Zebrafish Brain Atlas. Nat. Meth 2015, 12 (11), 1039–1046. 10.1038/nmeth.3581. PMC4710481 26778924

[ref6] Gendelev L.; Taylor J.; Myers-Turnbull D.; Chen S.; McCarroll M. N.; Arkin M. R.; Kokel D.; Keiser M. J. Deep Phenotypic Profiling of Neuroactive Drugs in Larval Zebrafish. bioRxiv 2024, 10.1101/2024.02.22.581657. PMC11570628 39551797

[ref7] Zhou K. C.; Harfouche M.; Cooke C. L.; Park J.; Konda P. C.; Kreiss L.; Kim K.; Jönsson J.; Doman T.; Reamey P.; Saliu V.; Cook C. B.; Zheng M.; Bechtel J. P.; Bègue A.; McCarroll M.; Bagwell J.; Horstmeyer G.; Bagnat M.; Horstmeyer R. Parallelized Computational 3D Video Microscopy of Freely Moving Organisms at Multiple Gigapixels per Second. Nat. Photonics 2023, 17 (5), 442–450. 10.1038/s41566-023-01171-7. 37808252 PMC10552607

[ref8] Bruni G.; Rennekamp A. J.; Velenich A.; McCarroll M.; Gendelev L.; Fertsch E.; Taylor J.; Lakhani P.; Lensen D.; Evron T.; Lorello P. J.; Huang X.-P.; Kolczewski S.; Carey G.; Caldarone B. J.; Prinssen E.; Roth B. L.; Keiser M. J.; Peterson R. T.; Kokel D. Zebrafish Behavioral Profiling Identifies Multitarget Antipsychotic-like Compounds. Nat. Chem. Biol. 2016, 12 (7), 559–566. 10.1038/nchembio.2097. 27239787 PMC4912417

[ref9] Monesson-Olson B.; McClain J. J.; Case A. E.; Dorman H. E.; Turkewitz D. R.; Steiner A. B.; Downes G. B. Expression of the Eight GABAA Receptor α Subunits in the Developing Zebrafish Central Nervous System. PLoS One 2018, 13 (4), e0196083 10.1371/journal.pone.0196083. 29702678 PMC5922542

[ref10] Renier C.; Faraco J. H.; Bourgin P.; Motley T.; Bonaventure P.; Rosa F.; Mignot E. Genomic and Functional Conservation of Sedative-Hypnotic Targets in the Zebrafish. Pharmacogenet. Genomics 2007, 17 (4), 237–253. 10.1097/FPC.0b013e3280119d62. 17496723

[ref11] Cocco A.; Carolina Rönnberg A. M.; Jin Z.; André G. I.; Vossen L. E.; Bhandage A. K.; Thörnqvist P.-O.; Birnir B.; Winberg S. Characterization of the γ-Aminobutyric Acid Signaling System in the Zebrafish (Danio Rerio Hamilton) Central Nervous System by Reverse Transcription-Quantitative Polymerase Chain Reaction. Neuroscience 2017, 343, 300–321. 10.1016/j.neuroscience.2016.07.018. 27453477

[ref12] Jembrek M. J.; Vlainic J. GABA Receptors: Pharmacological Potential and Pitfalls. Curr. Pharm. Des 2015, 21 (34), 4943–4959. 10.2174/1381612821666150914121624. 26365137

[ref13] Korpi E. R.; Sinkkonen S. T. GABAA Receptor Subtypes as Targets for Neuropsychiatric Drug Development. Pharmacology & Therapeutics 2006, 109 (1), 12–32. 10.1016/j.pharmthera.2005.05.009. 15996746

[ref14] Olsen R. W.; Sieghart W. GABAA Receptors: Subtypes Provide Diversity of Function and Pharmacology. Neuropharmacology 2009, 56 (1), 141–148. 10.1016/j.neuropharm.2008.07.045. 18760291 PMC3525320

[ref15] Brickley S. G.; Mody I. Extrasynaptic GABAA Receptors: Their Function in the CNS and Implications for Disease. Neuron 2012, 73 (1), 23–34. 10.1016/j.neuron.2011.12.012. 22243744 PMC3399243

[ref16] Chuang S.-H.; Reddy D. S. Genetic and Molecular Regulation of Extrasynaptic GABA-A Receptors in the Brain: Therapeutic Insights for Epilepsy. J. Pharmacol Exp Ther 2018, 364 (2), 180–197. 10.1124/jpet.117.244673. 29142081 PMC5771312

[ref17] McGrath M.; Hoyt H.; Pence A.; Jayakar S. S.; Zhou X.; Forman S. A.; Cohen J. B.; Miller K. W.; Raines D. E. Competitive Antagonism of Etomidate Action by Diazepam: In Vitro GABAA Receptor and In Vivo Zebrafish Studies. Anesthesiology 2020, 133 (3), 583–594. 10.1097/ALN.0000000000003403. 32541553 PMC7944240

[ref18] Pence A.; Hoyt H.; McGrath M.; Forman S. A.; Raines D. E. Competitive Interactions Between Propofol and Diazepam: Studies in GABAA Receptors and Zebrafish. J. Pharmacol Exp Ther 2022, 383 (3), 238–245. 10.1124/jpet.122.001337. 36167415 PMC9667980

[ref19] Gavande N.; Karim N.; Johnston G. A. R.; Hanrahan J. R.; Chebib M. Identification of Benzopyran-4-One Derivatives (Isoflavones) as Positive Modulators of GABA(A) Receptors. ChemMedChem 2011, 6 (8), 1340–1346. 10.1002/cmdc.201100120. 21560249

[ref20] Lu C.; Wang Y.; Wang D.; Zhang L.; Lv J.; Jiang N.; Fan B.; Liu X.; Wang F. Neuroprotective Effects of Soy Isoflavones on Scopolamine-Induced Amnesia in Mice. Nutrients 2018, 10 (7), 853 10.3390/nu10070853. 29966363 PMC6073222

[ref21] Wang F.; Yan Huen M. S.; Tsang S. Y.; Xue H. Neuroactive Flavonoids Interacting with GABAA Receptor Complex. Current Drug Targets - CNS & Neurological Disorders 2005, 4 (5), 575–585. 10.2174/156800705774322030. 16266290

[ref22] Matin A.; Doddareddy M. R.; Gavande N.; Nammi S.; Groundwater P. W.; Roubin R. H.; Hibbs D. E. The Discovery of Novel Isoflavone Pan Peroxisome Proliferator-Activated Receptor Agonists. Bioorg. Med. Chem. 2013, 21 (3), 766–778. 10.1016/j.bmc.2012.11.040. 23265844

[ref23] Kokel D.; Dunn T. W.; Ahrens M. B.; Alshut R.; Cheung C. Y. J.; Saint-Amant L.; Bruni G.; Mateus R.; van Ham T. J.; Shiraki T.; Fukada Y.; Kojima D.; Yeh J.-R. J.; Mikut R.; von Lintig J.; Engert F.; Peterson R. T. Identification of Nonvisual Photomotor Response Cells in the Vertebrate Hindbrain. J. Neurosci. 2013, 33 (9), 3834–3843. 10.1523/JNEUROSCI.3689-12.2013. 23447595 PMC3600642

[ref24] Fernandes A. M.; Fero K.; Arrenberg A. B.; Bergeron S. A.; Driever W.; Burgess H. A. Deep Brain Photoreceptors Control Light-Seeking Behavior in Zebrafish Larvae. Curr. Biol. 2012, 22 (21), 2042–2047. 10.1016/j.cub.2012.08.016. 23000151 PMC3494761

[ref25] Kokel D.; Cheung C. Y. J.; Mills R.; Coutinho-Budd J.; Huang L.; Setola V.; Sprague J.; Jin S.; Jin Y. N.; Huang X.-P.; Bruni G.; Woolf C. J.; Roth B. L.; Hamblin M. R.; Zylka M. J.; Milan D. J.; Peterson R. T. Photochemical Activation of TRPA1 Channels in Neurons and Animals. Nat. Chem. Biol. 2013, 9 (4), 257–263. 10.1038/nchembio.1183. 23396078 PMC3604056

[ref26] Lam P.-Y.; Thawani A. R.; Balderas E.; White A. J. P.; Chaudhuri D.; Fuchter M. J.; Peterson R. T. TRPswitch-A Step-Function Chemo-Optogenetic Ligand for the Vertebrate TRPA1 Channel. J. Am. Chem. Soc. 2020, 142 (41), 17457–17468. 10.1021/jacs.0c06811. 32966062 PMC8011302

[ref27] Cheng D.; McCarroll M. N.; Taylor J. C.; Wu T.; Kokel D. Identification of Compounds Producing Non-Visual Photosensation via TRPA1 in Zebrafish. bioRxiv 2020, 10.1101/2020.06.10.111203.

[ref28] Covello G.; Rossello F. J.; Filosi M.; Gajardo F.; Duchemin A.; Tremonti B. F.; Eichenlaub M.; Polo J. M.; Powell D.; Ngai J.; Allende M. L.; Domenici E.; Ramialison M.; Poggi L. Transcriptome Analysis of the Zebrafish Atoh7–/–Mutant, Lakritz, Highlights Atoh7-dependent Genetic Networks with Potential Implications for Human Eye Diseases. FASEB Bioadv 2020, 2 (7), 434–448. 10.1096/fba.2020-00030. 32676583 PMC7354691

[ref29] Bonsack F.; Sukumari-Ramesh S. TSPO: An Evolutionarily Conserved Protein with Elusive Functions. Int. J. Mol. Sci. 2018, 19 (6), 1694 10.3390/ijms19061694. 29875327 PMC6032217

[ref30] Chen S.; Gao L.; Li X.; Ye Y. Allopregnanolone in Mood Disorders: Mechanism and Therapeutic Development. Pharmacol. Res. 2021, 169, 105682 10.1016/j.phrs.2021.105682. 34019980

[ref31] Leonelli E.; Yague J. G.; Ballabio M.; Azcoitia I.; Magnaghi V.; Schumacher M.; Garcia-Segura L. M.; Melcangi R. C. Ro5–4864, a Synthetic Ligand of Peripheral Benzodiazepine Receptor, Reduces Aging-Associated Myelin Degeneration in the Sciatic Nerve of Male Rats. Mech Ageing Dev 2005, 126 (11), 1159–1163. 10.1016/j.mad.2005.06.001. 16045970

[ref32] Pellow S.; File S. E. Behavioural Actions of Ro 5–4864: A Peripheral-Type Benzodiazepine?. Life Sciences 1984, 35 (3), 229–240. 10.1016/0024-3205(84)90106-1. 6087055

[ref33] Gavioli E. C.; Duarte F. S.; Bressan E.; Ferrara P.; Farges R. C.; De Lima T. C. M. Antidepressant-like Effect of Ro5–4864, a Peripheral-Type Benzodiazepine Receptor Ligand, in Forced Swimming Test. Eur. J. Pharmacol. 2003, 471 (1), 21–26. 10.1016/S0014-2999(03)01789-8. 12809948

[ref34] Weissman B. A.; Cott J.; Hommer D.; Quirion R.; Paul S.; Skolnick P. Pharmacological, Electrophysiological, and Neurochemical Actions of the Convulsant Benzodiazepine Ro 5–4864 (4’-Chlordiazepam). Adv. Biochem. Psychopharmacol. 1983, 38, 139–151. 6670623

[ref35] Costa B.; Pozzo E. D.; Martini C. Translocator Protein as a Promising Target for Novel Anxiolytics. Curr. Top. Med. Chem. 2012, 12 (4), 270–285. 10.2174/156802612799078720. 22204481

[ref36] Sadamitsu K.; Shigemitsu L.; Suzuki M.; Ito D.; Kashima M.; Hirata H. Characterization of Zebrafish GABAA Receptor Subunits. Sci. Rep 2021, 11 (1), 6242 10.1038/s41598-021-84646-3. 33737538 PMC7973766

[ref37] Kent M. R.; Kara N.; Patton J. G. Inhibition of GABAA-ρ Receptors Induces Retina Regeneration in Zebrafish. Neural Regener. Res. 2021, 16 (2), 367–374. 10.4103/1673-5374.286972. PMC7896201 32859800

[ref38] Westerfield M. The Zebrafish Book: A Guide for the Laboratory Use of Zebrafish (Danio Rerio), 4th ed.; University of Oregon Press, 2020.

[ref39] Thyme S. B.; Pieper L. M.; Li E. H.; Pandey S.; Wang Y.; Morris N. S.; Sha C.; Choi J. W.; Herrera K. J.; Soucy E. R.; Zimmerman S.; Randlett O.; Greenwood J.; McCarroll S. A.; Schier A. F. Phenotypic Landscape of Schizophrenia-Associated Genes Defines Candidates and Their Shared Functions. Cell 2019, 177 (2), 478–491.e20. 10.1016/j.cell.2019.01.048. 30929901 PMC6494450

[ref40] Cachero S.; Ostrovsky A. D.; Yu J. Y.; Dickson B. J.; Jefferis G. S. X. E. Sexual Dimorphism in the Fly Brain. Curr. Biol. 2010, 20 (18), 1589–1601. 10.1016/j.cub.2010.07.045. 20832311 PMC2957842

[ref41] Ostrovsky A.; Cachero S.; Jefferis G. Clonal Analysis of Olfaction in Drosophila: Immunochemistry and Imaging of Fly Brains. Cold Spring Harb. Protoc. 2013, 2013 (4), 342–346. 10.1101/pdb.prot071720. 23547149

[ref42] Kim J. J.; Gharpure A.; Teng J.; Zhuang Y.; Howard R. J.; Zhu S.; Noviello C. M.; Walsh R. M.; Lindahl E.; Hibbs R. E. Shared Structural Mechanisms of General Anaesthetics and Benzodiazepines. Nature 2020, 585 (7824), 303–308. 10.1038/s41586-020-2654-5. 32879488 PMC7486282

