# Supplementary material for: A Multimodal, In Vivo Approach for Assessing Structurally and Phenotypically Related Neuroactive Molecules
Source: ACS Chem Neurosci. 2024 Sep 17;15(22):4171–84. doi: 10.1021/acschemneuro.4c00426 (PMC11587515; doi:10.1021/acschemneuro.4c00426)
Supplement: Supplementary file 1 — cn4c00426_si_001.pdf [file cn4c00426_si_001.pdf]

**Supplementary Material for**

# A Multimodal, In Vivo Approach for Assessing Structurally and Phenotypically Related Neuroactive Molecules

Matthew N McCarroll[1*], Elizabeth Sisko[1], Jung Ho Gong[5], Jinfeng Teng[3], Jack Taylor[2,4], Douglas Myers-Turnbull[2], Drew Young[2], Grant Burley[1], Lain X Pierce[1], Ryan Hibbs[3*], David Kokel[2], Jason K Sello[1*]

1. Department of Pharmaceutical Chemistry, University of California, San Francisco, San Francisco, CA, 94158, USA
2. Institute for Neurodegenerative Diseases, University of California, San Francisco, San Francisco, CA, 94158, USA
3. Department of Neurobiology, University of California, San Diego, CA, 92093, USA
4. UCSF Weill Institute for Neurosciences Memory and Aging Center, University of California, San Francisco, CA, 94158, USA
5. Department of Chemistry, Brown University, Providence, RI, 02912, USA

* Correspondence to: matthew.mccarroll@ucsf.edu, rehibbs@ucsd.edu, jason.sello@ucsf.edu

## SUPPLEMENTARY FIGURES

**S1a**

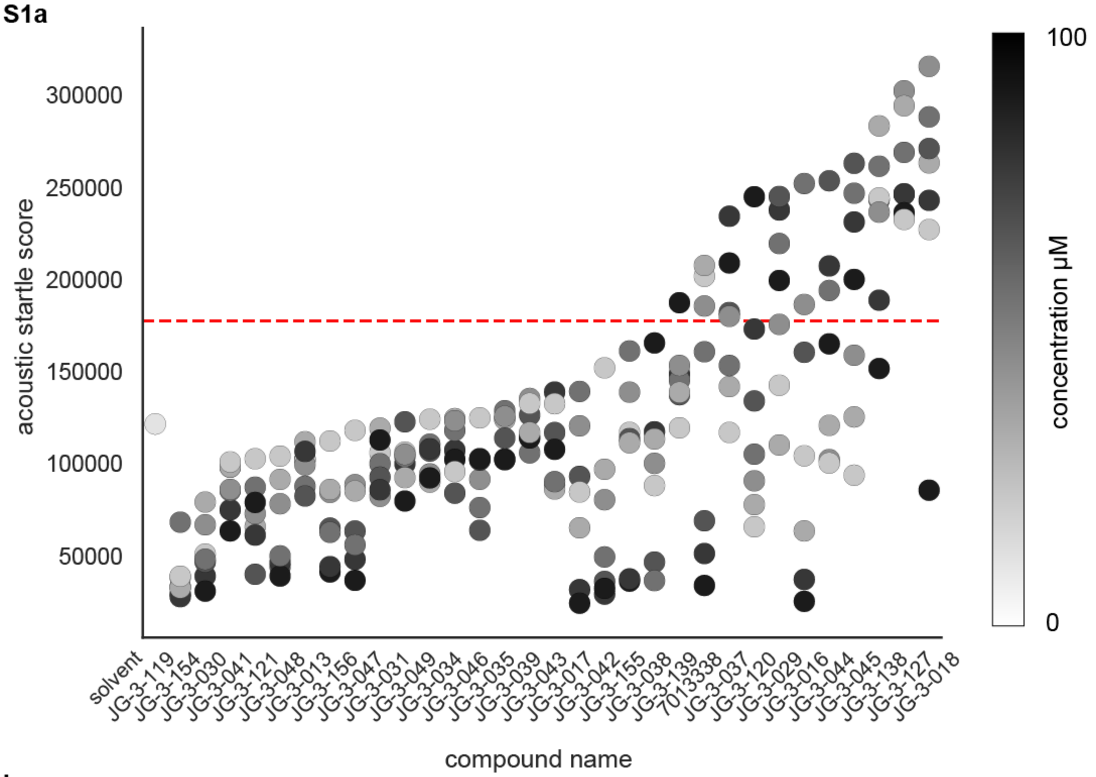


**b**

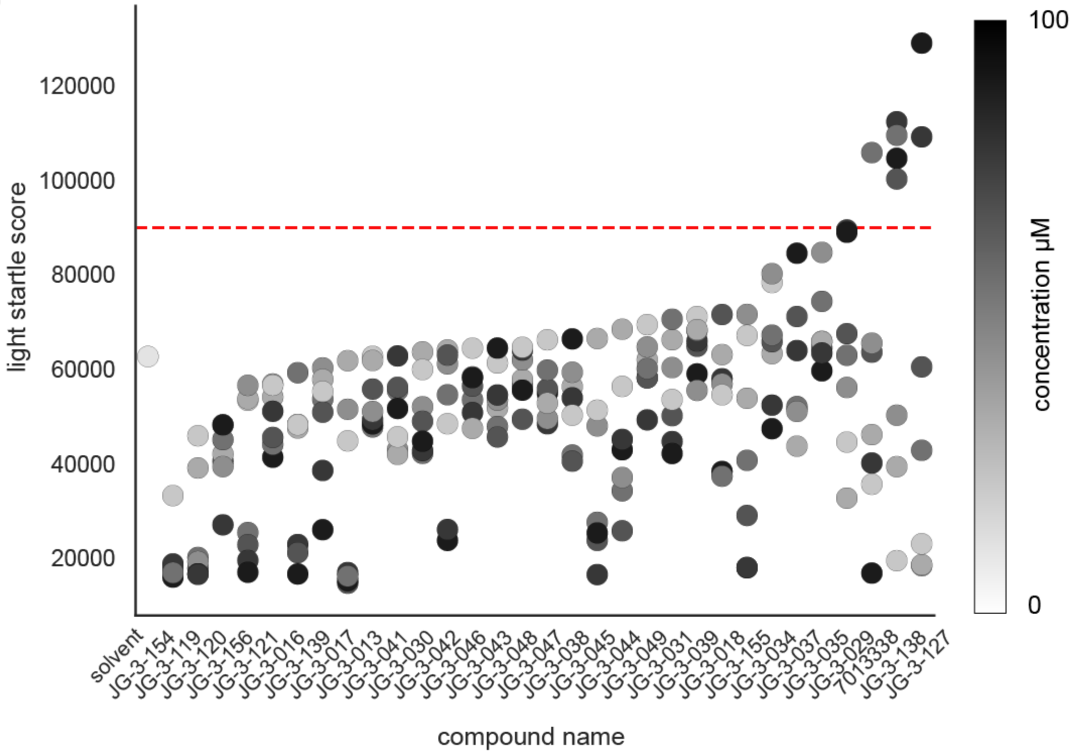


**Figure S1. Analysis of all Isoflavones quantifying the enhance acoustic startle and light startle responses in zebrafish larvae.** Zebrafish were treated with the indicated compounds and analyzed for changes in behavioral responses. (a) The strip plot quantifies acoustic startle response as a score

(*y*-axis) or a light startle score (b) in zebrafish treated with the indicated isoflavones (*x*-axis) at the indicated concentrations (color bar). Each point represents the average of *n* = 3-6 wells and 8 animals per well. Dotted red line represents 2 standard deviations above the vehicle treated wells.

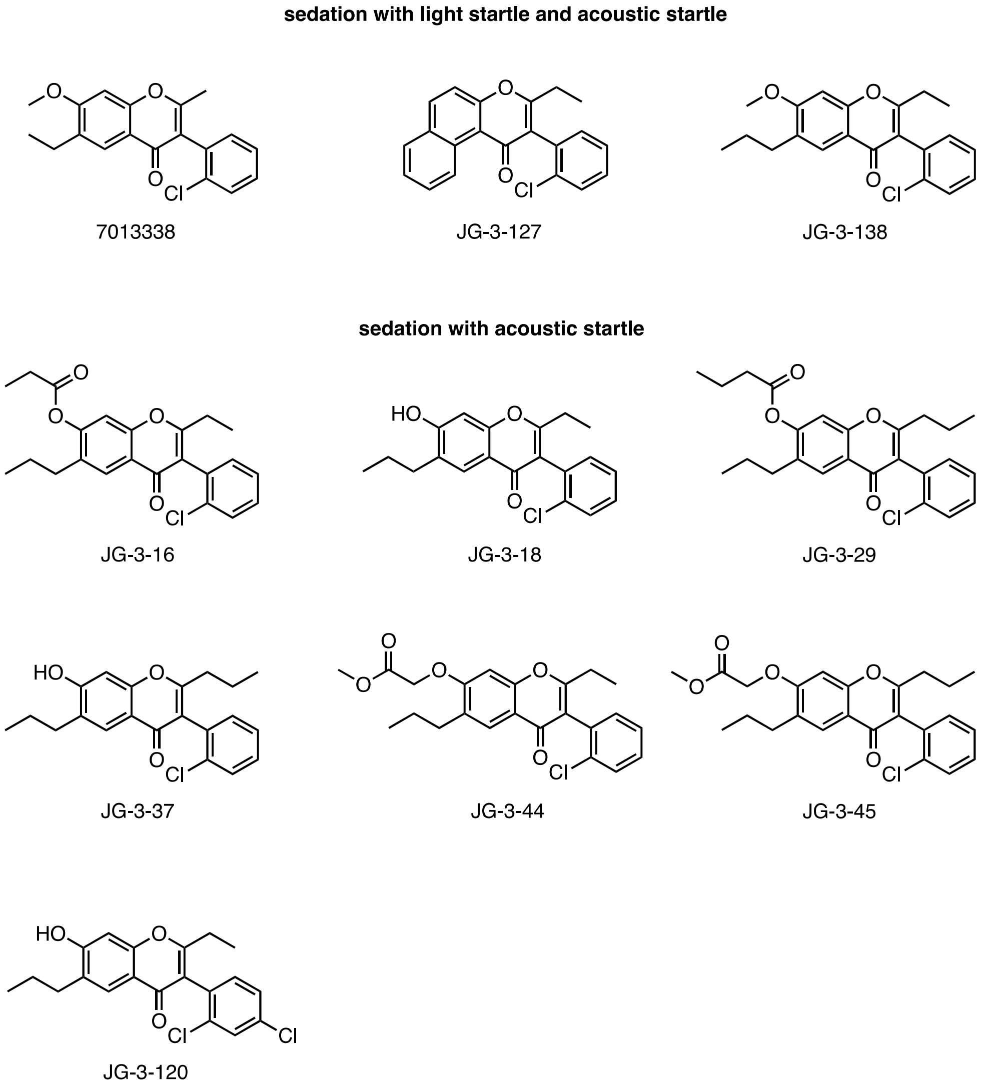


**Figure S2. Chemical structures of isoflavones with indicated behavioral modifications observed in the larval zebrafish.** The top 3 compounds show sedation in zebrafish with both light and acoustic startle responses. The bottom 7 compounds have both sedation and acoustic startle behaviors observed in larval zebrafish.

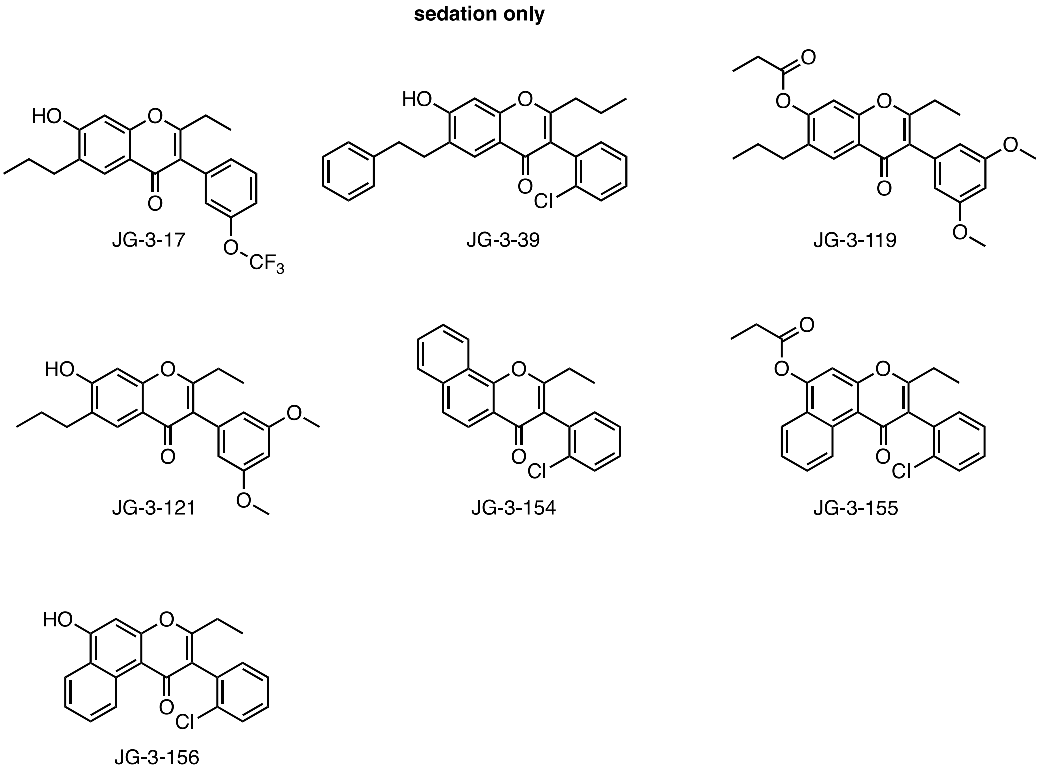


**Figure S3. Chemical structures of isoflavones with sedation only behavioral phenotypes in larval zebrafish.**

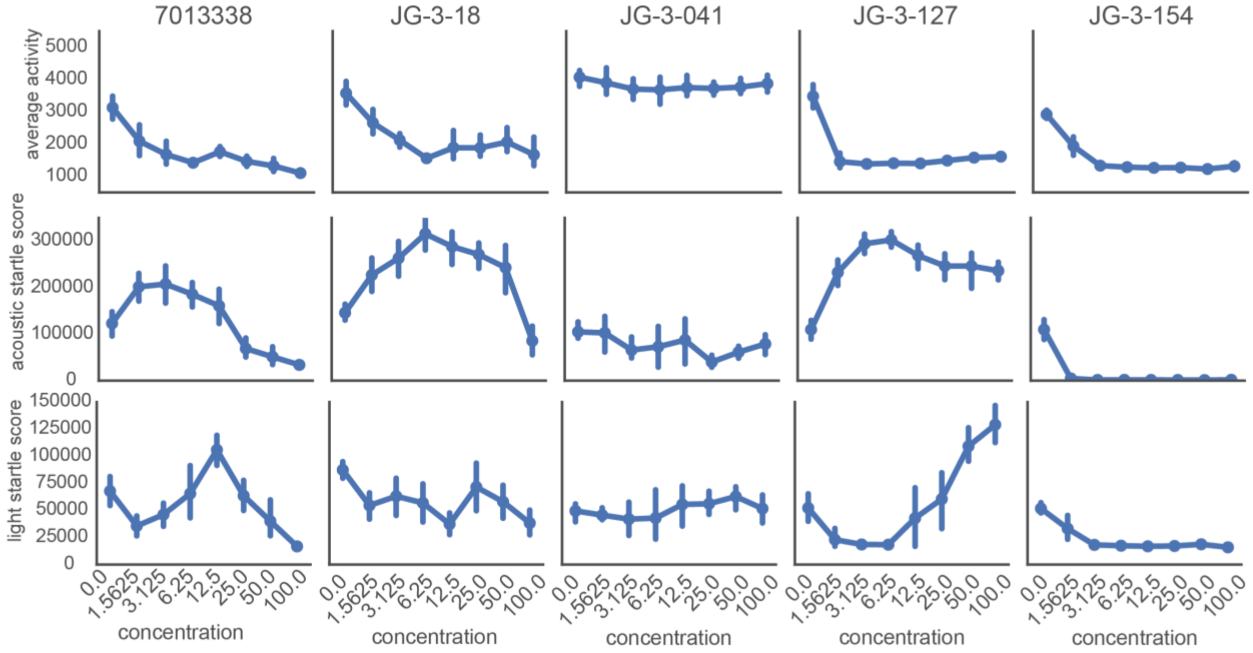


**Figure S4. Full dose response retesting of the parent isoflavone with the different phenotypic category hit compounds.** Average activity, acoustic startle, and light startle scores (y-axis) of

zebrafish treated with the indicated compounds (n = 6-12 wells/condition 8 fish/well) at the indicated concentrations in µM (x-axis).

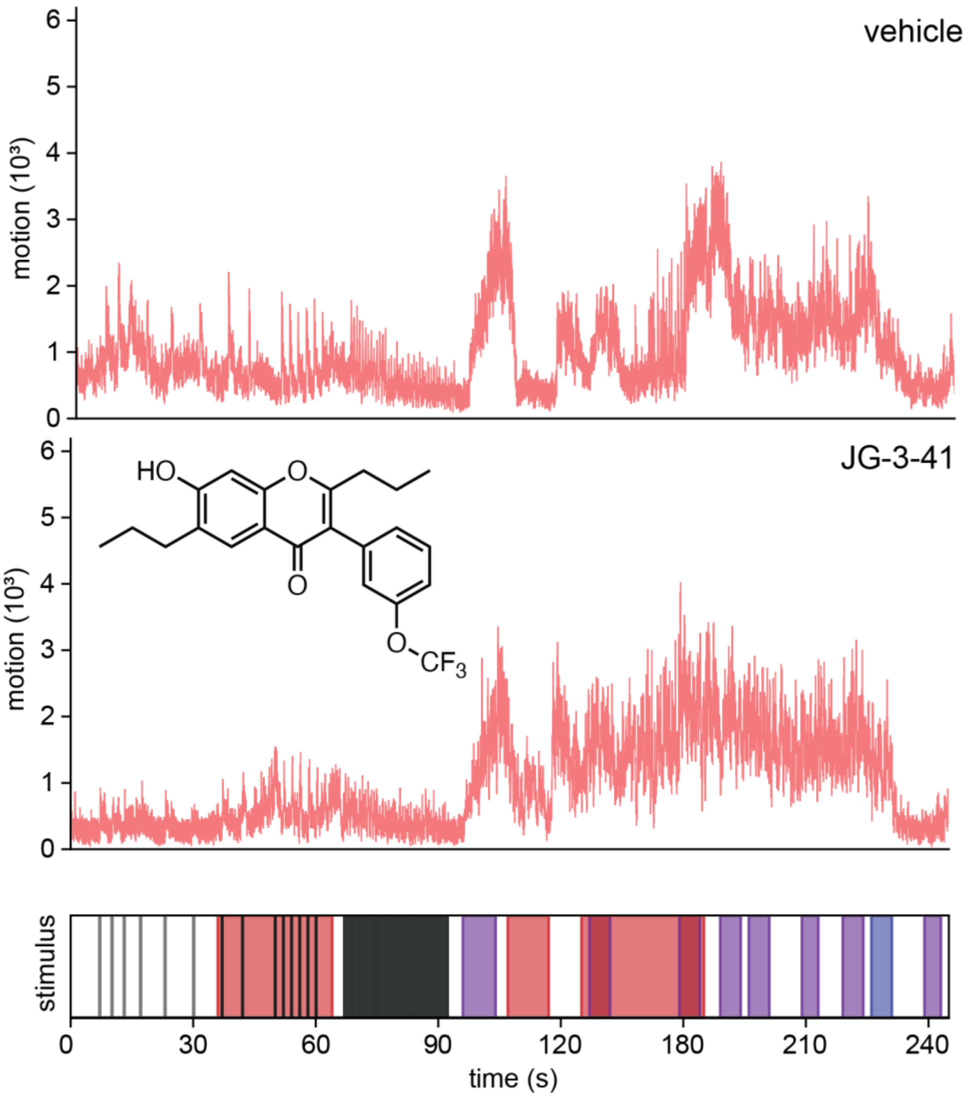


**Figure S5. Isoflavone analog JG-3-41 exhibits no behavioral modification in larval zebrafish.** The plots show motor activity (y-axis) of zebrafish treated with either vehicle control (top) or JG-3-41 (n = 6-12 wells). Colored bars above the x-axis represent the timing and duration of acoustic and light stimuli.

**Figure S6**

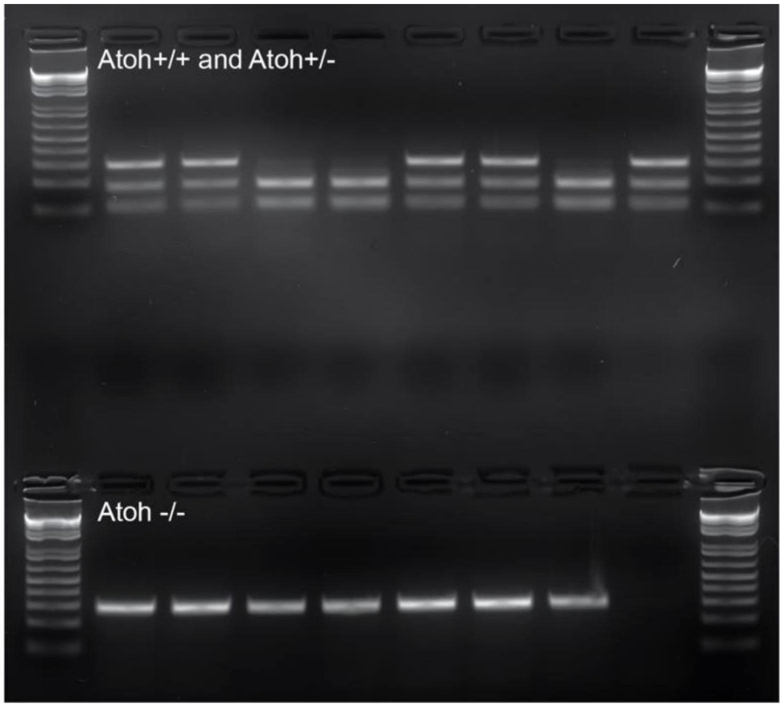


**Figure S6. Mutant genotyping.** DNA agarose gel showing the genotyping verification of the *atoh-/-* mutant, loss of a *stu-I* restriction enzyme cut site for the locus in the homozygous mutation indicating blind mutants.

## SUPPLEMENTARY TABLES

**Table S1. Chemical names and SMILES of synthesized isoflavone collection**

| Name | SMILES |
|---|---|
| 7013338 | CCc1cc2c(cc1OC)oc(C)c(-c1ccccc1Cl)c2=O |
| JG-3-029 | CCCC1=C(C=C2C(=C1)C(=O)C(=C(CCC)O2)C3=C(C=CC=C3)Cl)OC(=O)CCC |
| JG-3-030 | CCC1=C(C2=C(C=CC=C2)Cl)C(=O)C3=CC(=C(C=C3O1)OC(=O)CC)CCC4=CC=CC=C4 |
| JG-3-031 | CCCC1=C(C2=C(C=CC=C2)Cl)C(=O)C3=CC(=C(C=C3O1)OC(=O)CCC)CCC4=CC=CC=C4 |
| JG-3-034 | CCC1=C(C2=CC(=CC=C2)OC(F)(F)F)C(=O)C3=CC(=C(C=C3O1)OC(=O)CC)CCC4=CC=CC=C4 |
| JG-3-035 | CCCC1=C(C2=CC(=CC=C2)OC(F)(F)F)C(=O)C3=CC(=C(C=C3O1)OC(=O)CCC)CCC4=CC=CC=C4 |
| JG-3-037 | CCCC1=C(C=C2C(=C1)C(=O)C(=C(CCC)O2)C3=C(C=CC=C3)Cl)O |
| JG-3-038 | CCC1=C(C2=C(C=CC=C2)Cl)C(=O)C3=CC(=C(C=C3O1)O)CCC4=CC=CC=C4 |
| JG-3-039 | CCCC1=C(C2=C(C=CC=C2)Cl)C(=O)C3=CC(=C(C=C3O1)O)CCC4=CC=CC=C4 |
| JG-3-041 | FC(F)(F)OC1=CC(C(C(C2=CC(CCC)=C(C=C2O3)O)=O)=C3CCC)=CC=C1 |
| JG-3-042 | CCC1=C(C2=CC(=CC=C2)OC(F)(F)F)C(=O)C3=CC(=C(C=C3O1)O)CCC4=CC=CC=C4 |
| JG-3-043 | CCCC1=C(C2=CC(=CC=C2)OC(F)(F)F)C(=O)C3=CC(=C(C=C3O1)O)CCC4=CC=CC=C4 |
| JG-3-044 | CCCC1=C(C=C2C(=C1)C(=O)C(=C(CC)O2)C3=C(C=CC=C3)Cl)OCC(=O)OC |
| JG-3-045 | CCCC1=C(C=C2C(=C1)C(=O)C(=C(CCC)O2)C3=C(C=CC=C3)Cl)OCC(=O)OC |
| JG-3-046 | CCC1=C(C2=C(C=CC=C2)Cl)C(=O)C3=CC(=C(C=C3O1)OCC(=O)OC)CCC4=CC=CC=C4 |
| JG-3-047 | CCCC1=C(C2=C(C=CC=C2)Cl)C(=O)C3=CC(=C(C=C3O1)OCC(=O)OC)CCC4=CC=CC=C4 |
| JG-3-048 | CCC1=C(C2=CC(=CC=C2)OC(F)(F)F)C(=O)C3=CC(=C(C=C3O1)OCC(=O)OC)CCC4=CC=CC=C4 |
| JG-3-049 | CCCC1=C(C2=CC(=CC=C2)OC(F)(F)F)C(=O)C3=CC(=C(C=C3O1)OCC(=O)OC)CCC4=CC=CC=C4 |
| JG-3-018 | OC1=C(CCC)C=C2C(OC(CC)=C(C3=C(Cl)C=CC=C3)C2=O)=C1 |
| JG-3-013 | CCC1=C(C2=CC=CC(OC(F)(F)F)=C2)C(C3=CC(CCC)=C(OC(CC)=O)C=C3O1)=O |
| JG-3-016 | CCC1=C(C2=C(Cl)C=CC=C2)C(C3=CC(CCC)=C(OC(CC)=O)C=C3O1)=O |
| JG-3-017 | CCC1=C(C2=CC=CC(OC(F)(F)F)=C2)C(C3=CC(CCC)=C(O)C=C3O1)=O |
| JG-3-119* | CCCC1=C(C=C2C(=C1)C(=O)C(=C(CC)O2)C3=CC(=CC(=C3)OC)OC)OC(=O)CC |
| JG-3-120* | CCCC1=C(C=C2C(=C1)C(=O)C(=C(CC)O2)C3=C(C=C(C=C3)Cl)Cl)O |
| JG-3-121* | CCCC1=C(C=C2C(=C1)C(=O)C(=C(CC)O2)C3=CC(=CC(=C3)OC)OC)O |
| JG-3-127* | CCC1=C(C(C2=C(O1)C=CC3=C2C=CC=C3)=O)C4=C(Cl)C=CC=C4 |
| JG-3-155* | CCC1=C(C2=C(C=CC=C2)Cl)C(=O)C3=C4C=CC=CC4=C(C=C3O1)OC(=O)CC |
| JG-3-139* | CCC1=C(C2=C(C=CC=C2)Cl)C(=O)C3=C4C=CC(=CC4=CC=C3O1)Br |
| JG-3-154* | CCC1=C(C2=C(C=CC=C2)Cl)C(=O)C3=C(C4=C(C=CC=C4)C=C3)O1 |
| JG-3-156* | CCC1=C(C2=C(C=CC=C2)Cl)C(=O)C3=C4C=CC=CC4=C(C=C3O1)O |
| JG-3-138* | CCCC1=C(C=C2C(=C1)C(=O)C(=C(CC)O2)C3=C(C=CC=C3)Cl)OC |

* First report of newly synthesized Isoflavone analogs

**Table S2. Structure behavior relationship of isoflavones**

| Compound | R1 (position 6) | R2 (position 3) | R3 (position 2) | R4 (position 7) | Sedation Index | eASR Index | Light Startle Index |
|---|---|---|---|---|---|---|---|
| JG-3-029 | propyl | 2-chlorophenyl | propyl | 1-oxobutoxy | 62.02 | 77.63 | 69.33 |
| JG-3-127 | 6,7-benzannulation | 2-chlorophenyl | ethyl | 6,7-benzannulation | 57.79 | 95.79 | 100 |
| JG-3-154 | hydro | 2-chlorophenyl | ethyl | 7,8-benzannulation | 54.42 | 25.09 | 25.76 |
| 7013338 | ethyl | 2-chlorophenyl | methyl | methoxy | 50.62 | 65.77 | 82.03 |
| JG-3-138 | propyl | 2-chlorophenyl | ethyl | methoxy | 50.35 | 89.76 | 87.1 |
| JG-3-119 | propyl | 3,5-dimethoxyphenyl | ethyl | 1-oxopropoxy | 48.97 | 21.63 | 35.57 |
| JG-3-039 | 2-phenylethyl | 2-chlorophenyl | propyl | hydroxyl | 48.41 | 42.89 | 55.13 |
| JG-3-016 | propyl | 2-chlorophenyl | ethyl | 1-oxopropoxy | 47.39 | 78 | 45.92 |
| JG-3-037 | propyl | 2-chlorophenyl | propyl | hydroxyl | 46.69 | 74.22 | 65.52 |
| JG-3-017 | propyl | 3-trifluoromethoxyphenyl | ethyl | hydroxyl | 45.98 | 44.1 | 47.9 |
| JG-3-045 | propyl | 2-chlorophenyl | propyl | methyl ethanoate ether | 43.63 | 83.33 | 51.53 |
| JG-3-018 | propyl | 2-chlorophenyl | ethyl | hydroxyl | 42.44 | 100 | 55.47 |
| JG-3-044 | propyl | 2-chlorophenyl | ethyl | methyl ethanoate ether | 40.78 | 80.32 | 53.05 |
| JG-3-120 | propyl | 2,4-dichlorophenyl | ethyl | hydroxyl | 39.87 | 77.59 | 37.36 |
| JG-3-156 | 5,6-benzannulation | 2-chlorophenyl | ethyl | hydroxyl | 32.71 | 37.41 | 43.85 |
| JG-3-155 | 5,6-benzannulation | 2-chlorophenyl | ethyl | 1-oxopropoxy | 32.45 | 51.07 | 55.49 |
| JG-3-121 | propyl | 3,5-dimethoxyphenyl | ethyl | hydroxyl | 31.64 | 33 | 44.07 |
| JG-3-042 | 2-phenylethyl | 3-trifluoromethoxyphenyl | ethyl | hydroxyl | 21.79 | 48.16 | 51.46 |
| JG-3-013 | 5,6-benzannulation | 2-chlorophenyl | ethyl | hydroxyl | 21.49 | 35.57 | 48.61 |
| JG-3-038 | 2-phenylethyl | 2-chlorophenyl | ethyl | hydroxyl | 17.31 | 52.44 | 51.46 |
| JG-3-047 | 2-phenylethyl | 2-chlorophenyl | propyl | methyl ethanoate ether | 15.92 | 37.79 | 51.31 |
| JG-3-048 | 2-phenylethyl | 3-trifluoromethoxyphenyl | ethyl | methyl ethanoate ether | 15.75 | 35.46 | 50.09 |
| JG-3-046 | 2-phenylethyl | 2-chlorophenyl | ethyl | methyl ethanoate ether | 15.21 | 39.56 | 49.64 |
| JG-3-030 | 2-phenylethyl | 2-chlorophenyl | ethyl | 1-oxopropoxy | 12.93 | 32 | 49.33 |
| JG-3-049 | 2-phenylethyl | 3-trifluoromethoxyphenyl | propyl | methyl ethanoate ether | 11.98 | 39.36 | 53.8 |
| JG-3-041 | propyl | 3-trifluoromethoxyphenyl | propyl | hydroxyl | 10.88 | 32.54 | 48.67 |
| JG-3-035 | 2-phenylethyl | 3-trifluoromethoxyphenyl | propyl | 1-oxobutoxy | 10.65 | 40.79 | 65.66 |
| JG-3-043 | 2-phenylethyl | 3-trifluoromethoxyphenyl | propyl | hydroxyl | 7.51 | 44 | 49.96 |
| JG-3-031 | 2-phenylethyl | 2-chlorophenyl | propyl | 1-oxobutoxy | 6.69 | 38.88 | 54.7 |
| JG-3-034 | 2-phenylethyl | 3-trifluoromethoxyphenyl | ethyl | 1-oxopropoxy | -1.33 | 39.42 | 62.18 |
| JG-3-139 | 5,6-(2-bromo)benzannulation | 2-chlorophenyl | ethyl | hydro | -9.92 | 59.36 | 46.71 |

**Table S3. Neuroanatomical regions significantly reduced in animals treated with 7013338 vs vehicle presented with light and acoustic stimulus**

| Neuroanatomical region name | label used to identify brain region | signal in ROI |
|---|---|---|
| Telencephalon - Subpallial dopaminergic cluster | EtVmat2-GFP | 64417.7998 |
| Telencephalon - Olfactory bulb | Gad1b-GFP | 64309.447 |
| Telencephalon - Vmat2 cluster | EtVmat2-GFP | 64290.0128 |
| Telencephalon - Subpallial Otpb Cluster 2 | Gad1b-GFP | 55404.1027 |
| Telencephalon - Isl1 cluster 1 | Gad1b-GFP | 53400.7802 |
| Telencephalon - Subpallial Gad1b cluster | Gad1b-GFP | 53250.4728 |
| Telencephalon - S1181t Cluster | Gad1b-GFP | 49949.2353 |
| Telencephalon - Pallium | Anti-tERK | 48602.9549 |
| Telencephalon - Olfactory Bulb | Vglut2a-GFP | 46143.8541 |
| Telencephalon - | Anti-tERK | 44391.8616 |
| Telencephalon - Subpallium | Gad1b-GFP | 40399.5138 |
| Telencephalon - Telencephalic Migrated Area 4 (M4) | Anti-Zn1 | 37470.0323 |
| Telencephalon - Anterior Commisure | Anti-Zrf2 | 37445.6061 |
| Telencephalon - Vglut2 rind | Vglut2a-GFP | 35294.1512 |
| Rhombencephalon - Otpb Cluster 4 | Otpb.A-Gal4-UAS-GCaMP_6 | 32010.6324 |
| Rhombencephalon - Otpb Cluster 3 | Otpb.A-Nsfb-GFP | 23477.2983 |
| Telencephalon - Subpallial Otpb strip | Vglut2a-GFP | 21321.5359 |
| Rhombencephalon - Qrfp neuron cluster sparse | Vglut2a-GFP | 20467.8982 |
| Rhombencephalon - Vglut2 cluster 1 | Vglut2a-GFP | 18190.0775 |
| Rhombencephalon - Gad1b Cluster 4 | Elavl3-H2BRFP | 17071.0615 |
| Telencephalon - Isl1 cluster 2 | Isl1-GFP | 16796.9497 |
| Rhombencephalon - Isl1 Cluster 3 | Vglut2a-GFP | 16594.925 |
| Telencephalon - Olig2 Cluster | Gad1b-GFP | 15296.3475 |
| Diencephalon - Eminentia Thalami | Vglut2a-GFP | 15219.1688 |
| Mesencephalon - Oxtl Cluster Sparse | Anti-Zrf1(GFAP) | 15066.652 |
| Rhombencephalon - Olig2 Cluster | Olig2-GFP | 14870.5878 |
| Rhombencephalon - Vmat2 Stripe3 | EtVmat2-GFP | 14512.5128 |
| Rhombencephalon - Isl1 Cluster 1 | Isl1-GFP | 14027.1609 |
| Rhombencephalon - Vglut2 cluster 2 | Vglut2a-GFP | 13597.6179 |
| Rhombencephalon - Gad1b Cluster 18 | Gad1b-GFP | 13567.2398 |

**Table S4. Neuroanatomical regions significantly increased in animals treated with 7013338 vs vehicle presented with light and acoustic stimulus**

| Neuroanatomical region name | label used to identify brain region | signal in ROI |
|---|---|---|
| Ganglia - Lateral Line Neuromast D1 | Pet1-GFP | 47952.3097 |
| Ganglia - Lateral Line Neuromast D2 | Anti-Zrf2 | 47498.2791 |
| Mesencephalon - Retinal Arborization Field 7 (AF7) | Isl2bGal4-uasDendra | 36343.479 |
| Ganglia - Lateral Line Neuromast SO1 | Olig2-GFP | 30179.8924 |
| Rhombencephalon - Gad1b Cluster 20 | Anti-Zrf1(GFAP) | 23278.5673 |
| Ganglia - Lateral Line Neuromast OC1 | Anti-Zrf1(GFAP) | 19821.013 |
| Spinal Cord - Dorsal Sparse Isl1 cluster | Anti-GlyR | 18951.3217 |
| Ganglia - Lateral Line Neuromast N | Anti-GlyR | 18512.9944 |
| Rhombencephalon - Noradrendergic neurons | Anti-Zrf1(GFAP) | 13401.3587 |
| Spinal Cord - Vglut2 Stripe 1 | Elavl3-H2BRFP | 13156.4952 |
| Ganglia - Lateral Line Neuromast SO2 | Anti-Zrf1(GFAP) | 13076.4338 |
| Spinal Cord - Vmat2 Stripe1 | Elavl3-H2BRFP | 11221.1512 |
| Ganglia - Olfactory Epithelium | Olig2-GFP | 9675.7854 |
| Spinal Cord - Glyt2 Stripe | Anti-Zrf1(GFAP) | 9593.7015 |
| Rhombencephalon - Area Postrema | Anti-TH | 7584.5797 |
| Diencephalon - Retinal Arborization Field 6 (AF6) | Isl2bGal4-uasDendra | 6997.6997 |
| Spinal Cord - Neurons with descending projections spinal backfills | Anti-Zrf1(GFAP) | 4223.2519 |
| Spinal Cord - 6.7FDhcrtR-Gal4 Stripe | Anti-Zrf1(GFAP) | 3933.6481 |
| Diencephalon - Retinal Arborization Field 5 (AF5) | Isl2bGal4-uasDendra | 3587.4592 |
| Rhombencephalon - Vglut2 Stripe 1 | Anti-Zrf1(GFAP) | 3576.942 |
| Spinal Cord | Anti-Zrf1(GFAP) | 3156.0939 |
| Ganglia - Lateral Line Neuromast SO3 | Anti-Zrf1(GFAP) | 2356.3236 |
| Spinal Cord - Neuropil Region | Anti-Znp1(Synaptotagmin2) | 2012.8067 |
| Diencephalon - Pineal Vmat2 cluster | Anti-TH | 1999.4928 |
| Diencephalon - Migrated Area of the Pretectum (M1) | Isl2bGal4-uasDendra | 1923.6612 |
| Rhombencephalon - Lateral Reticular Nucleus | Anti-Zrf1(GFAP) | 1882.9675 |
| Rhombencephalon - Vmat2 Stripe1 | EtVmat2-GFP | 1842.3845 |
| Rhombencephalon - Rhombomere 7 | Anti-Zrf1(GFAP) | 1790.363 |
| Rhombencephalon - Neuropil Region 2 | Anti-Znp1(Synaptotagmin2) | 1584.7121 |
| Rhombencephalon - Neuropil Region 3 | Anti-GlyR | 1481.5834 |

**Table S5. Neuroanatomical regions significantly reduced in animals treated with JG-127 vs vehicle presented with light and acoustic stimulus.**

| Neuroanatomical region name | label used to identify brain region | signal in ROI |
|---|---|---|
| Telencephalon - Subpallial dopaminergic cluster | EtVmat2-GFP | 65533.9836 |
| Telencephalon - Vmat2 cluster | EtVmat2-GFP | 65142.2213 |
| Telencephalon - Olfactory bulb dopaminergic neuron areas | Gad1b-GFP | 63391.0468 |
| Telencephalon - Isl1 cluster 1 | Gad1b-GFP | 60283.2513 |
| Telencephalon - Subpallial Otpb Cluster 2 | Gad1b-GFP | 57540.1532 |
| Telencephalon - Subpallial Gad1b cluster | Gad1b-GFP | 54250.1041 |
| Telencephalon - S1181t Cluster | Gad1b-GFP | 51416.8133 |
| Telencephalon - Telencephalic Migrated Area 4 (M4) | Anti-Zn1 | 48656.6838 |
| Diencephalon - Pineal | Anti-TH | 48523.0139 |
| Telencephalon - Pallium | Anti-tERK | 46841.8721 |
| Rhombencephalon - Olig2 Cluster | Olig2-GFP | 45535.0534 |
| Telencephalon - Olfactory Bulb | Vglut2a-GFP | 45408.956 |
| Telencephalon - | Anti-tERK | 44603.4292 |
| Telencephalon - Subpallium | Gad1b-GFP | 42510.105 |
| Telencephalon - Anterior Commisure | Anti-Zrf2 | 40429.6921 |
| Rhombencephalon - Glyt2 Cluster 2 | EtVmat2-GFP | 37654.8605 |
| Rhombencephalon - Otpb Cluster 4 | Otpb.A-Gal4-UAS-GCaMP_6 | 37597.6512 |
| Rhombencephalon - Gad1b Cluster 14 | 6.7FRhcrtR-Gal4-uasKaede | 36448.4611 |
| Rhombencephalon - Qrfp neuron cluster sparse | Vglut2a-GFP | 34204.8282 |
| Rhombencephalon - Gad1b Cluster 18 | Gad1b-GFP | 32808.7359 |
| Rhombencephalon - 6.7FDhcrtR-Gal4 Stripe 1 | Gad1b-GFP | 32021.4809 |
| Telencephalon - Vglut2 rind | Vglut2a-GFP | 31944.3871 |
| Rhombencephalon - 6.7FDhcrtR-Gal4 Stripe 2 | 6.7FRhcrtR-Gal4-uasKaede | 30689.1722 |
| Rhombencephalon - Vglut2 Stripe 2 | Gad1b-GFP | 30106.101 |
| Rhombencephalon - Vmat2 Stripe3 | EtVmat2-GFP | 29507.7138 |
| Rhombencephalon - Glyt2 Cluster 13 | Anti-Znp1(Synaptotagmin2) | 28886.1973 |
| Rhombencephalon - Otpb Cluster 3 | Otpb.A-Nsfb-GFP | 28245.3254 |
| Rhombencephalon - Isl1 Cluster 3 | Vglut2a-GFP | 27465.0938 |
| Rhombencephalon - Glyt2 Stripe 2 | Elavl3-H2BRFP | 27056.1247 |
| Rhombencephalon - 6.7FDhcrtR-Gal4 Cluster 5 | 6.7FRhcrtR-Gal4-uasKaede | 26980.79 |

**Table S6. Neuroanatomical regions significantly increased in animals treated with JG-154 vs vehicle presented with light and acoustic stimulus.**

| Neuroanatomical region name | label used to identify brain region | signal in ROI |
|---|---|---|
| Telencephalon - Subpallial dopaminergic cluster | EtVmat2-GFP | 65535 |
| Telencephalon - Vmat2 cluster | EtVmat2-GFP | 65318.8159 |
| Telencephalon - Olfactory bulb | Gad1b-GFP | 64736.9728 |
| Telencephalon - Isl1 cluster 1 | Gad1b-GFP | 57436.545 |
| Telencephalon - Subpallial Otpb Cluster 2 | Gad1b-GFP | 55606.5851 |
| Telencephalon - Subpallial Gad1b cluster | Gad1b-GFP | 52931.4544 |
| Telencephalon - Pallium | Anti-tERK | 51406.6326 |
| Telencephalon - Telencephalic Migrated Area 4 (M4) | Anti-Zn1 | 48719.3378 |
| Telencephalon - S1181t Cluster | Gad1b-GFP | 48268.2419 |
| Telencephalon - Olfactory Bulb | Vglut2a-GFP | 47330.2613 |
| Telencephalon - | Anti-tERK | 46220.5387 |
| Mesencephalon - Oxtl Cluster Sparse | Anti-Zrf1(GFAP) | 40636.683 |
| Mesencephalon - Retinal Arborization Field 9 (AF9) | Anti-Zrf2 | 40340.7912 |
| Telencephalon - Subpallium | Gad1b-GFP | 39473.4027 |
| Telencephalon - Vglut2 rind | Vglut2a-GFP | 38065.4541 |
| Mesencephalon - Tecum Neuropil | Isl2bGal4-uasDendra | 36589.4688 |
| Mesencephalon - Medial Tectal Band | Gad1b-GFP | 35600.65 |
| Telencephalon - Anterior Commisure | Anti-Zrf2 | 35335.7316 |
| Mesencephalon - Retinal Arborization Field 7 (AF7) | Isl2bGal4-uasDendra | 34824.0213 |
| Rhombencephalon - Otpb Cluster 4 | Otpb.A-Gal4-UAS-GCaMP_6 | 29661.1498 |
| Mesencephalon - Retinal Arborization Field 8 (AF8) | EtVmat2-GFP | 26509.0047 |
| Mesencephalon - | Isl2bGal4-uasDendra | 23074.5987 |
| Rhombencephalon - Olig2 Cluster | Olig2-GFP | 22594.094 |
| Diencephalon - Pineal | Anti-TH | 22242.8482 |
| Mesencephalon - Tectum Stratum Periventriculare | Elavl3-H2BRFP | 21251.2551 |
| Diencephalon - Retinal Arborization Field 5 (AF5) | Isl2bGal4-uasDendra | 18868.1678 |
| Telencephalon - Subpallial Otpb strip | Vglut2a-GFP | 16791.214 |
| Rhombencephalon - Otpb Cluster 3 | Otpb.A-Nsfb-GFP | 15861.3153 |
| Mesencephalon - Torus Semicircularis | Anti-Znp1(Synaptotagmin2) | 14912.4246 |
| Diencephalon - Pretectum | Anti-Zrf1(GFAP) | 14829.9167 |

**Table S7. Neuroanatomical regions significantly increased in animals treated with 7013338 vs JG-154 presented with light and acoustic stimulus.**

| Neuroanatomical region name | label used to identify brain region | signal in ROI |
|---|---|---|
| Mesencephalon - Retinal Arborization Field 7 (AF7) | Isl2bGal4-uasDendra | 64444.5401 |
| Diencephalon - Retinal Arborization Field 5 (AF5) | Isl2bGal4-uasDendra | 47912.7646 |
| Mesencephalon - Tecum Neuropil | Isl2bGal4-uasDendra | 38232.9695 |
| Mesencephalon - Retinal Arborization Field 8 (AF8) | EtVmat2-GFP | 29930.5498 |
| Diencephalon - Migrated Area of the Pretectum (M1) | Isl2bGal4-uasDendra | 29019.1996 |
| Diencephalon - Retinal Arborization Field 6 (AF6) | Isl2bGal4-uasDendra | 27521.2222 |
| Ganglia - Lateral Line Neuromast D1 | Anti-Zn12(Hnk-1) | 26635.5409 |
| Mesencephalon - | Isl2bGal4-uasDendra | 17163.4047 |
| Mesencephalon - Tectum Stratum Periventriculare | Elavl3-H2BRFP | 13405.3874 |
| Mesencephalon - Medial Tectal Band | Gad1b-GFP | 11722.1365 |
| Mesencephalon - Retinal Arborization Field 9 (AF9) | Anti-Zrf2 | 11327.9753 |
| Spinal Cord - Dorsal Sparse Isl1 cluster | Anti-GlyR | 10921.7235 |
| Ganglia - Lateral Line Neuromast SO1 | Olig2-GFP | 10511.7861 |
| Ganglia - Lateral Line Neuromast D2 | Anti-GlyR | 8285.0769 |
| Ganglia - Lateral Line Neuromast N | Anti-GlyR | 8211.6047 |
| Spinal Cord - Vmat2 Stripe1 | Elavl3-H2BRFP | 6618.8375 |
| Rhombencephalon - 6.7FDhcrtR-Gal4 Cluster 5 | EtVmat2-GFP | 6542.8592 |
| Spinal Cord - Vglut2 Stripe 1 | Elavl3-H2BRFP | 6185.9161 |
| Mesencephalon - Torus Semicircularis | Anti-5HT | 5508.2549 |
| Diencephalon - Right Habenula Vglut2 Cluster | Vglut2a-GFP | 4896.5592 |
| Spinal Cord - Glyt2 Stripe | Elavl3-H2BRFP | 4776.3531 |
| Mesencephalon - Tegmentum | Gad1b-GFP | 4043.6846 |
| Ganglia - Olfactory Epithelium | Olig2-GFP | 3025.2181 |
| Diencephalon - Habenula | Vglut2a-GFP | 2949.7384 |
| Rhombencephalon - Noradrendergic neurons of theVagal areas | Anti-Zrf1(GFAP) | 2764.7814 |
| Spinal Cord - Neurons with descending projections by spinal backfills | Anti-Zrf1(GFAP) | 2465.903 |
| Diencephalon - Retinal Arborization Field 4 (AF4) | Isl2bGal4-uasDendra | 2352.453 |
| Spinal Cord - 6.7FDhcrtR-Gal4 Stripe | Anti-Zrf1(GFAP) | 2304.9016 |
| Diencephalon - Dorsal Thalamus | Elavl3-H2BRFP | 1946.6192 |
| Rhombencephalon - Lobus caudalis cerebelli | Olig2-GFP | 1918.6333 |

**Table S8. Neuroanatomical regions significantly increased in animals treated with JG-127 vs JG-154 presented with light and acoustic stimulus.**

| Neuroanatomical region name | label used to identify brain region | signal in ROI |
|---|---|---|
| Mesencephalon - Medial Tectal Band | Gad1b-GFP | 20938.7415 |
| Mesencephalon - Tecum Neuropil | Isl2bGal4-uasDendra | 15470.6883 |
| Mesencephalon - Tectum Stratum Periventriculare | Elavl3-H2BRFP | 7625.62 |
| Mesencephalon | Isl2bGal4-uasDendra | 6807.8276 |

**Table S9. Neuroanatomical regions significantly activated in JG-127 treated animals in response to light stimulius**

| Neuroanatomical region name | label used to identify brain region | signal in ROI |
|---|---|---|
| Mesencephalon - Tecum Neuropil | Isl2bGal4-uasDendra | 28251.6321 |
| Diencephalon - Migrated Area of the Pretectum (M1) | Anti-Zrf2 | 20581.8364 |
| Mesencephalon - Tectum Stratum Periventriculare | Elavl3-H2BRFP | 16860.8737 |
| Mesencephalon - Medial Tectal Band | Gad1b-GFP | 15978.2395 |
| Diencephalon - Dorsal Thalamus | Elavl3-H2BRFP | 15856.29 |
| Diencephalon - Retinal Arborization Field 5 (AF5) | Isl2bGal4-uasDendra | 14962.4079 |
| Mesencephalon | Isl2bGal4-uasDendra | 14484.816 |
| Diencephalon - Hypothalamus Gad1b Cluster 3 Sparse | Elavl3-H2BRFP | 14241.0546 |
| Diencephalon - Retinal Arborization Field 6 (AF6) | Isl2bGal4-uasDendra | 13335.4826 |
| Diencephalon - Ventral Thalamus | Gad1b-GFP | 11075.9482 |
| Diencephalon - Retinal Arborization Field 4 (AF4) | Isl2bGal4-uasDendra | 10922.8495 |
| Diencephalon - Hypothalamus Vglut2 Cluster 6 | Elavl3-H2BRFP | 7995.1362 |
| Diencephalon - Hypothalamus 6.7FRhcrtR-Gal4 cluster 1 | 6.7FRhcrtR-Gal4-uasKaede | 6236.0943 |
| Mesencephalon - Isl1 cluster of the mesencephalic region | Gad1b-GFP | 5522.4708 |
| Diencephalon - Hypothalamus Gad1b Cluster 1 | Gad1b-GFP | 5272.2179 |
| Mesencephalon - Retinal Arborization Field 8 (AF8) | Isl2bGal4-uasDendra | 4440.2654 |
| Mesencephalon - Torus Semicircularis | Gad1b-GFP | 3686.9674 |
| Diencephalon - Olig2 Band | Olig2-GFP | 3470.829 |
| Diencephalon - Hypothalamus 6.7FRhcrtR-Gal4 cluster 2 | Elavl3-H2BRFP | 3297.0241 |
| Diencephalon - Anterior pretectum cluster of vmat2 Neurons | EtVmat2-GFP | 3243.3545 |
| Diencephalon - Retinal Arborization Field 3 (AF3) | Anti-Zrf2 | 2922.0352 |
| Diencephalon | Elavl3-H2BRFP | 2893.7308 |
| Diencephalon - Hypothalamus Vglut2 Cluster 3 | Vglut2a-GFP | 2771.4039 |
| Mesencephalon - Tegmentum | Gad1b-GFP | 2538.9865 |
| Diencephalon - Hypothalamus Gad1b Cluster 2 | Anti-Zrf2 | 2385.128 |
| Diencephalon - Medial vglut2 cluster | Elavl3-H2BRFP | 2298.7565 |
| Diencephalon - Intermediate Hypothalamus | Anti-tERK | 2100.4608 |

**Table S10. Neuroanatomical regions significantly reduced in Atoh -/- mutant animals treated with JG-127 and presented light stimulus.**

| Neuroanatomical region name | label used to identify brain region | signal in ROI |
|---|---|---|
| Diencephalon - Anterior group of posterior tubercular vmat2 neurons | Hcrt-RFP | 62211.6488 |
| Mesencephalon - Retinal Arborization Field 8 (AF8) | EtVmat2-GFP | 58110.8104 |
| Diencephalon - Oxtl Cluster 3 | Oxtl-GFP | 56333.736 |
| Diencephalon - Hypothalamus 6.7FRhcrtR-Gal4 cluster 2 | Elavl3-H2BRFP | 54073.0035 |
| Diencephalon - Oxtl Cluster 5 | Oxtl-GFP | 54058.5302 |
| Mesencephalon - Ptf1a Cluster | Ptf1aGal4-uasKaede | 52442.9128 |
| Mesencephalon - Isl1 cluster of the mesencephalic region | Gad1b-GFP | 50689.2248 |
| Diencephalon - Dopaminergic Cluster 2 - posterior tuberculum | Qrfp-GFP | 50654.465 |
| Diencephalon - Migrated Area of the Pretectum (M1) | Anti-Zrf2 | 50570.1917 |
| Mesencephalon - Otpb Cluster | Isl1-GFP | 49936.8584 |
| Mesencephalon - Oculomotor Nucleus nIII | Isl1-GFP | 49456.0253 |
| Diencephalon - Hypothalamus s1181t Cluster | Qrfp-GFP | 45314.3053 |
| Diencephalon - Hypothalamus Hcrt Neurons | Hcrt-RFP | 45197.4461 |
| Diencephalon - Medial vglut2 cluster | Vglut2a-GFP | 44241.4094 |
| Mesencephalon - Sparse 6.7FRhcrtR cluster | 6.7FRhcrtR-Gal4-uasKaede | 43660.3482 |
| Mesencephalon - NucMLF (nucleus of the medial longitudinal fascicle) | SpinalBackfills | 39412.0028 |
| Mesencephalon - Tegmentum | Elavl3-H2BRFP | 38488.4395 |
| Mesencephalon - Vglut2 cluster 1 | Vglut2a-GFP | 38296.8286 |
| Diencephalon - Retinal Arborization Field 5 (AF5) | Isl2bGal4-uasDendra | 36704.514 |
| Diencephalon - Dopaminergic Cluster 3 - hypothalamus | Hcrt-RFP | 35177.1511 |
| Diencephalon - Hypothalamus 6.7FRhcrtR-Gal4 cluster 1 | 6.7FRhcrtR-Gal4-uasKaede | 34988.4192 |
| Diencephalon - Posterior Tuberculum | Qrfp-GFP | 34244.0906 |
| Diencephalon - Isl1 cluster 3 | Elavl3-H2BRFP | 29222.9195 |
| Mesencephalon - Tecum Neuropil | Isl2bGal4-uasDendra | 26839.4098 |
| Diencephalon - Otpb Cluster 1 | Anti-TH | 24439.9637 |
| Mesencephalon - Retinal Arborization Field 9 (AF9) | Anti-Zrf2 | 23772.6521 |
| Mesencephalon - Torus Semicircularis | Anti-5HT | 22307.0857 |
| Mesencephalon | Isl2bGal4-uasDendra | 22135.6584 |
| Rhombencephalon - Isl1 Cluster 1 | Isl1-GFP | 21784.4766 |

## Spectral Analysis of Isoflavone Analogs

JG-3-013

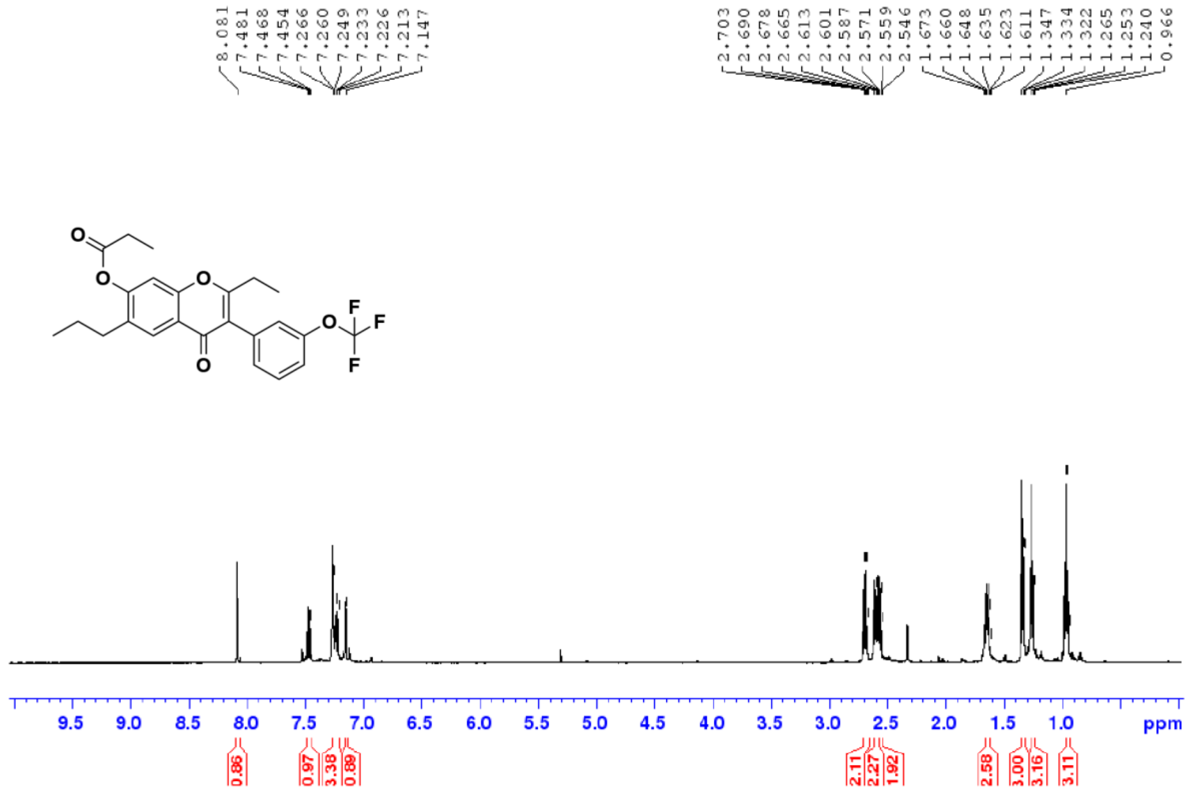


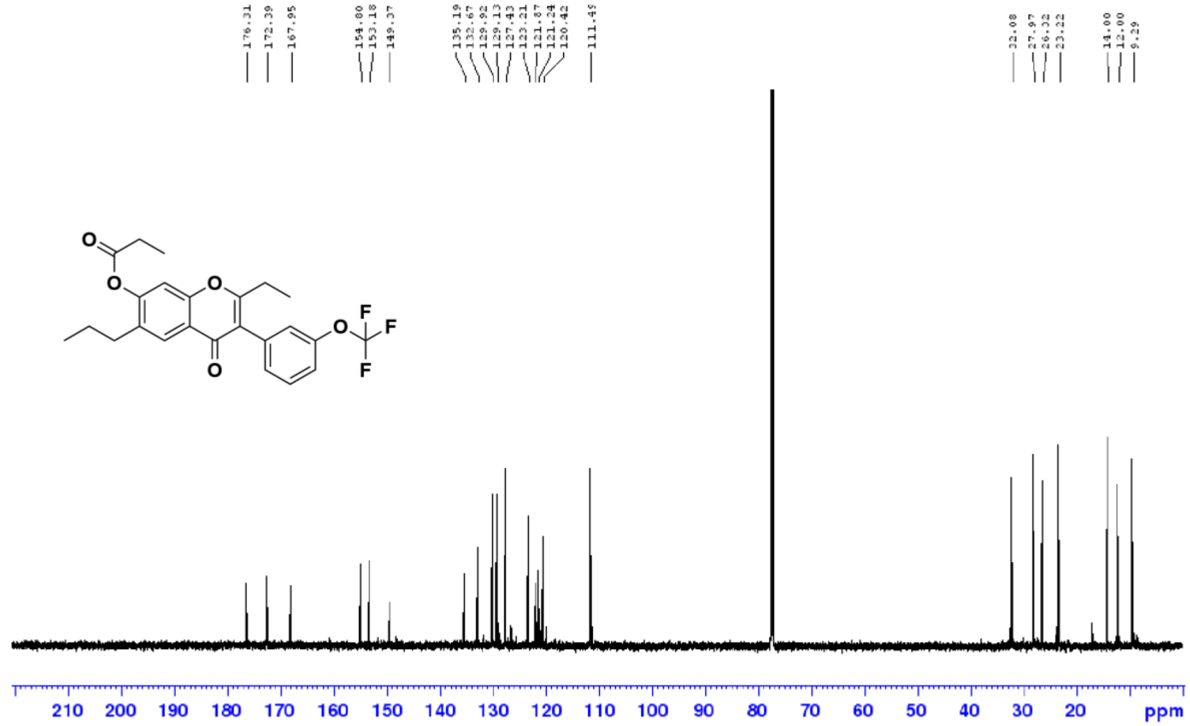

JG-3-016

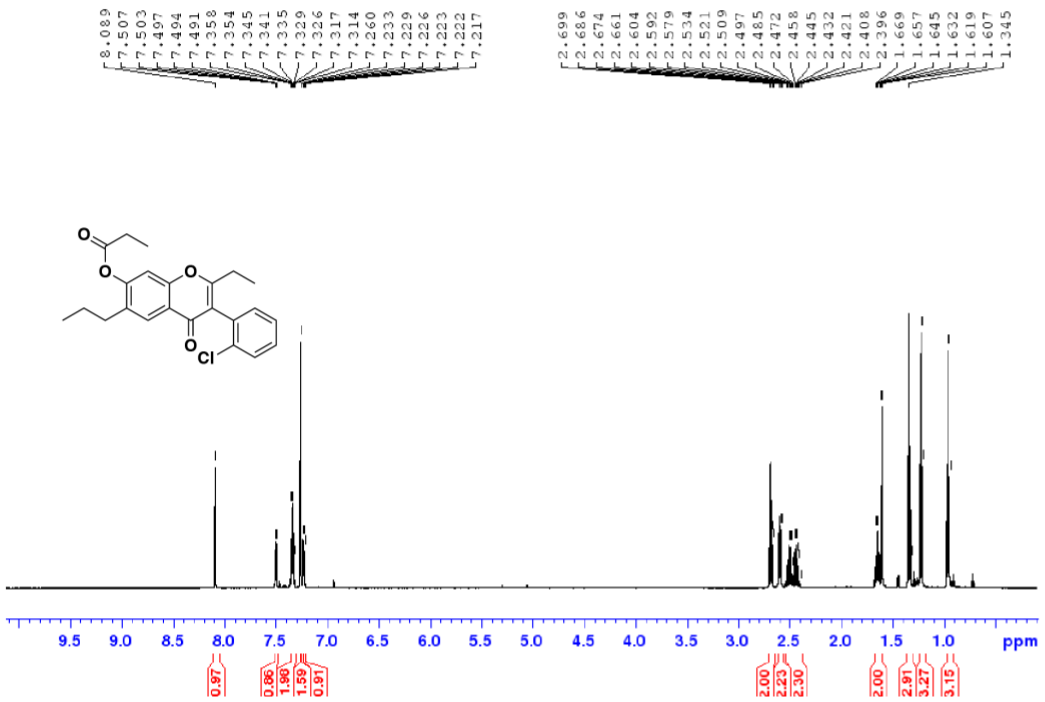
8.089 7.507 7.503 7.497 7.494 7.491 7.358 7.354 7.345 7.341 7.335 7.329 7.326 7.317 7.314 7.260 7.233 7.229 7.226 7.223 7.222 7.217
2.699 2.686 2.674 2.661 2.604 2.592 2.579 2.534 2.521 2.509 2.497 2.485 2.472 2.458 2.445 2.432 2.421 2.408 2.396 1.669 1.657 1.645 1.632 1.619 1.607 1.345
O
O
O
O
Cl
9.5 9.0 8.5 8.0 7.5 7.0 6.5 6.0 5.5 5.0 4.5 4.0 3.5 3.0 2.5 2.0 1.5 1.0 ppm
0.97 0.86 1.98 1.59 0.91 2.00 2.23 2.30 2.00 2.91 3.27 3.15

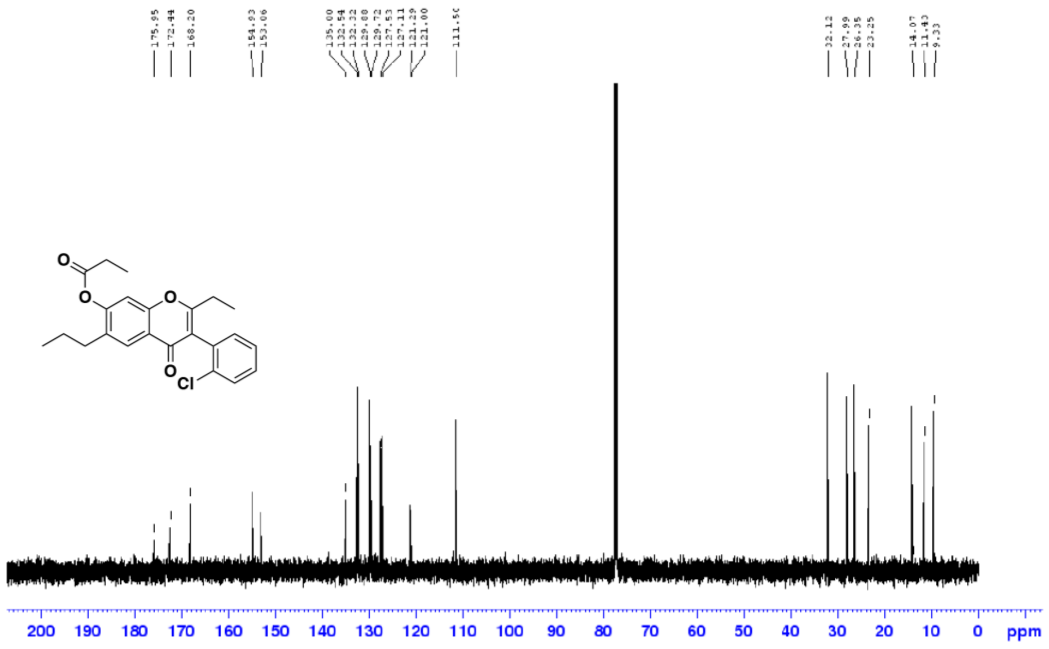
175.95 172.44 168.20 154.93 153.06 135.00 132.54 132.32 129.80 129.72 127.53 127.11 121.29 121.00 111.50
32.12 27.99 26.35 23.25 14.07 11.43 9.33
O
O
O
O
Cl
200 190 180 170 160 150 140 130 120 110 100 90 80 70 60 50 40 30 20 10 0 ppm

JG-3-017

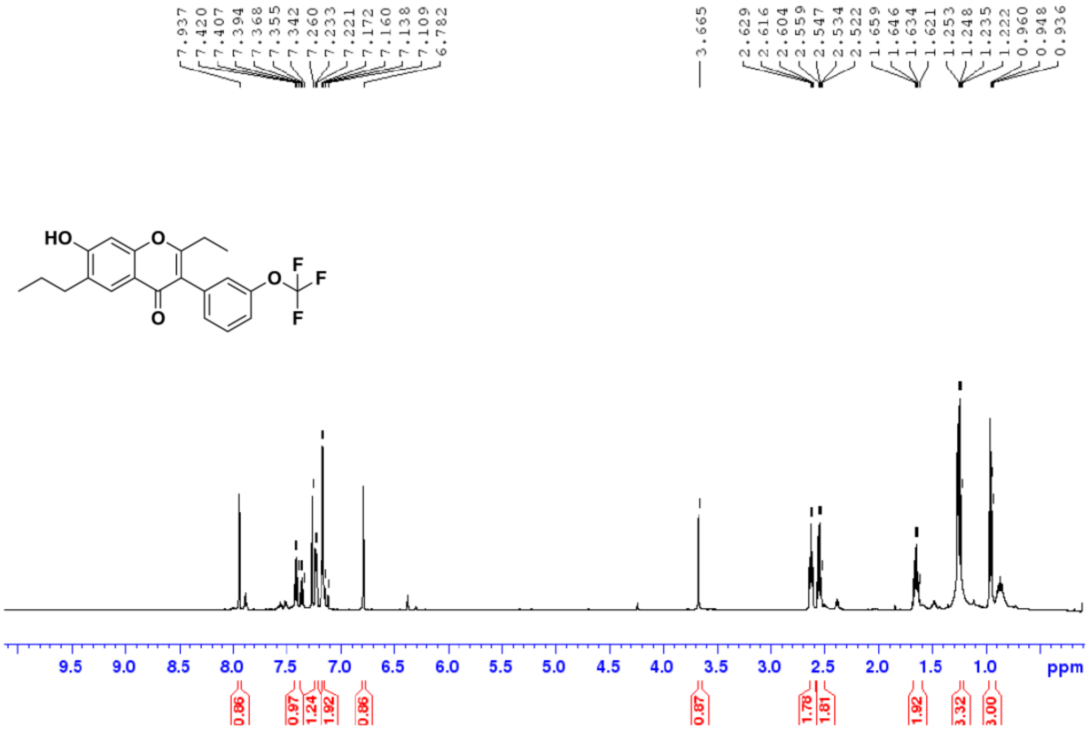
7.937
7.420
7.407
7.394
7.368
7.355
7.342
7.260
7.233
7.221
7.172
7.160
7.138
7.109
6.782
3.665
2.629
2.616
2.604
2.559
2.547
2.534
2.522
1.659
1.646
1.634
1.621
1.253
1.248
1.235
1.222
0.960
0.948
0.936
HO
O
O
O
F
F
F
9.5 9.0 8.5 8.0 7.5 7.0 6.5 6.0 5.5 5.0 4.5 4.0 3.5 3.0 2.5 2.0 1.5 1.0 ppm
0.86
0.97
1.24
1.92
0.86
0.87
1.78
1.81
1.92
3.32
3.00

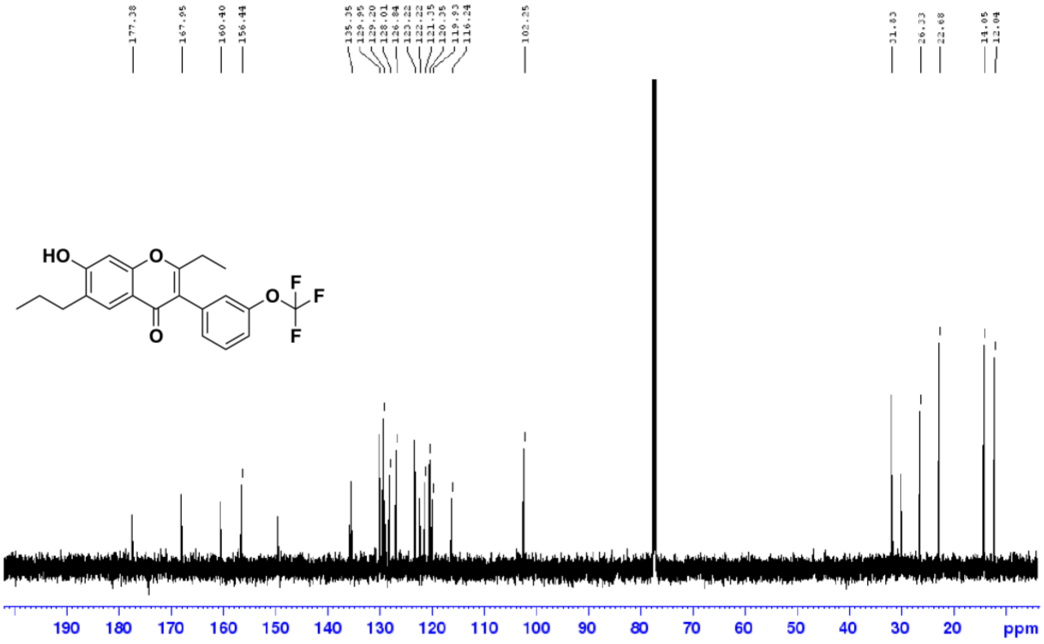
177.38
167.95
160.40
156.44
135.35
129.95
129.20
128.01
126.84
123.22
122.22
121.35
120.35
119.93
116.24
102.25
31.83
26.33
22.68
14.05
12.04
HO
O
O
O
F
F
F
190 180 170 160 150 140 130 120 110 100 90 80 70 60 50 40 30 20 ppm

JG-3-018

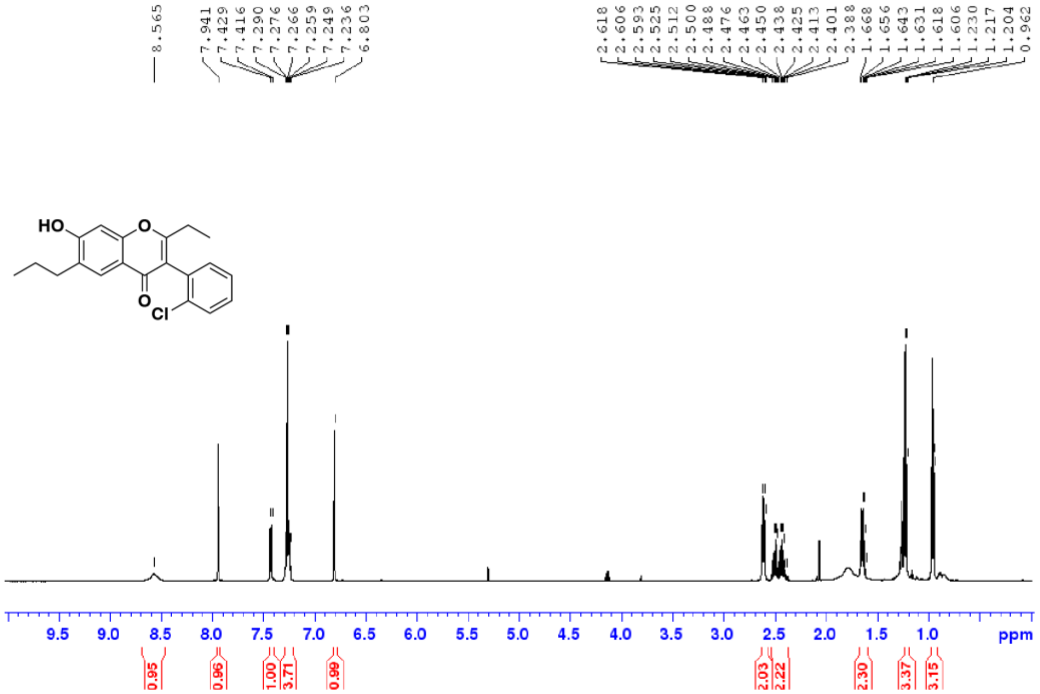
8.565
7.941
7.429
7.416
7.290
7.276
7.266
7.259
7.249
7.236
6.803
2.618
2.606
2.593
2.525
2.512
2.500
2.488
2.476
2.463
2.450
2.438
2.425
2.413
2.401
2.388
1.668
1.656
1.643
1.631
1.618
1.606
1.230
1.217
1.204
0.962
HO
O
O
Cl
9.5
9.0
8.5
8.0
7.5
7.0
6.5
6.0
5.5
5.0
4.5
4.0
3.5
3.0
2.5
2.0
1.5
1.0
ppm
0.95
0.96
1.00
3.71
0.99
2.03
2.22
2.30
3.37
3.15

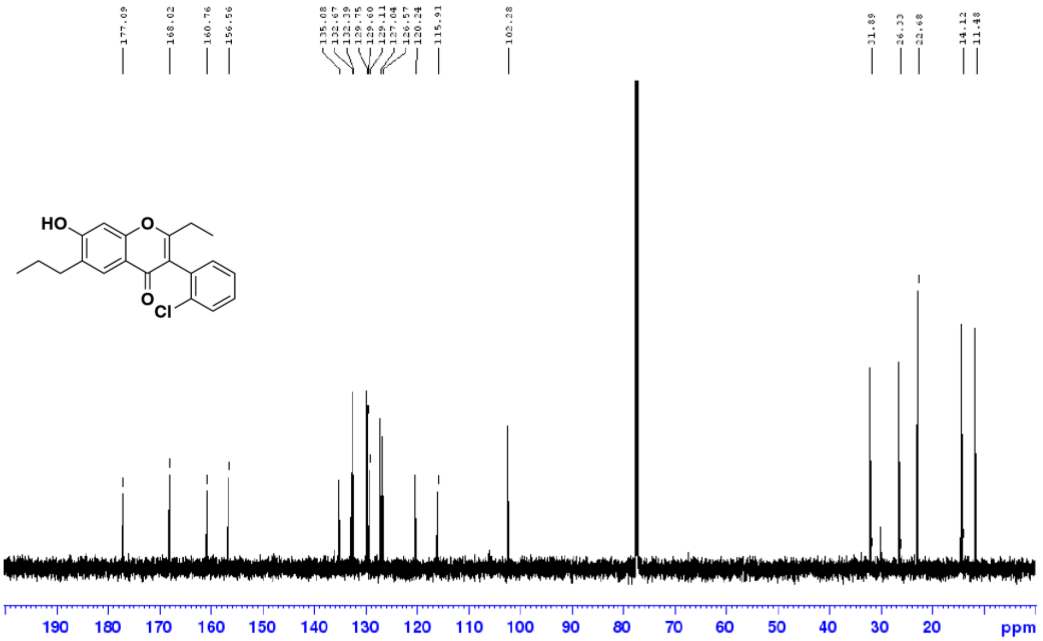
177.09
168.02
160.76
156.56
135.08
132.67
132.39
129.75
129.60
129.11
127.04
126.57
120.24
115.91
102.28
31.89
26.33
22.68
14.12
11.48
HO
O
O
Cl
190
180
170
160
150
140
130
120
110
100
90
80
70
60
50
40
30
20
ppm

JG-3-029

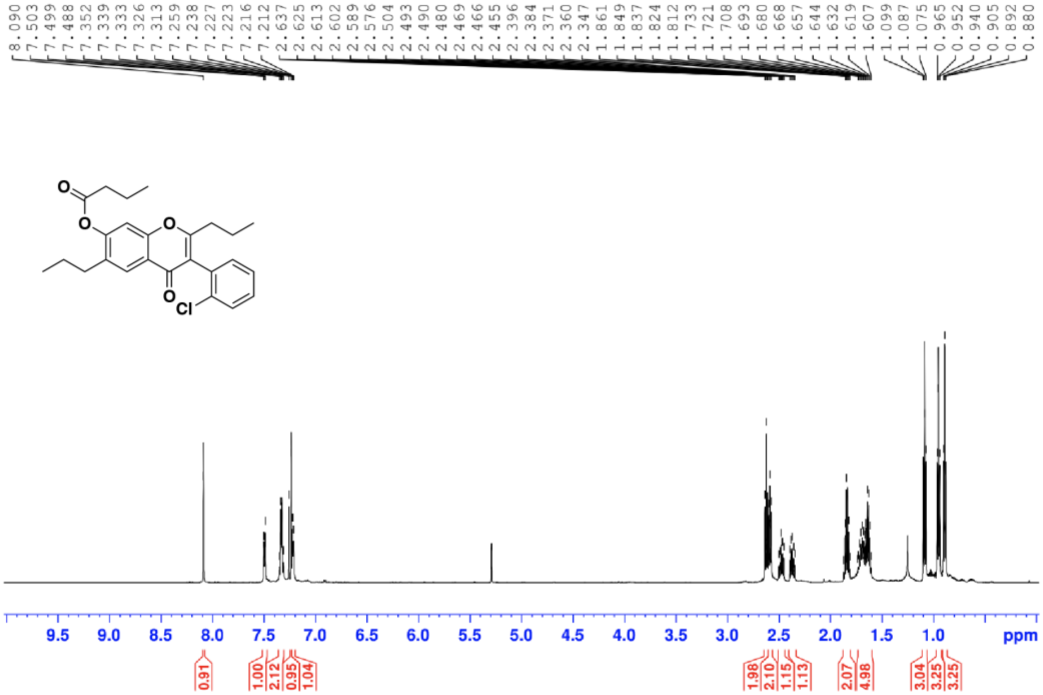
8.090
7.503
7.499
7.488
7.352
7.339
7.333
7.326
7.313
7.259
7.238
7.227
7.223
7.216
7.212
2.637
2.625
2.613
2.602
2.589
2.576
2.504
2.493
2.490
2.480
2.469
2.466
2.455
2.396
2.384
2.371
2.360
2.347
1.861
1.849
1.837
1.824
1.812
1.733
1.721
1.708
1.693
1.680
1.668
1.657
1.644
1.632
1.619
1.607
1.099
1.087
1.075
0.965
0.952
0.940
0.905
0.892
0.880
O
O
O
O
Cl
9.5 9.0 8.5 8.0 7.5 7.0 6.5 6.0 5.5 5.0 4.5 4.0 3.5 3.0 2.5 2.0 1.5 1.0 ppm
0.91
1.00
2.12
0.95
1.04
1.98
2.10
1.15
1.13
2.07
4.98
3.04
3.25
3.25

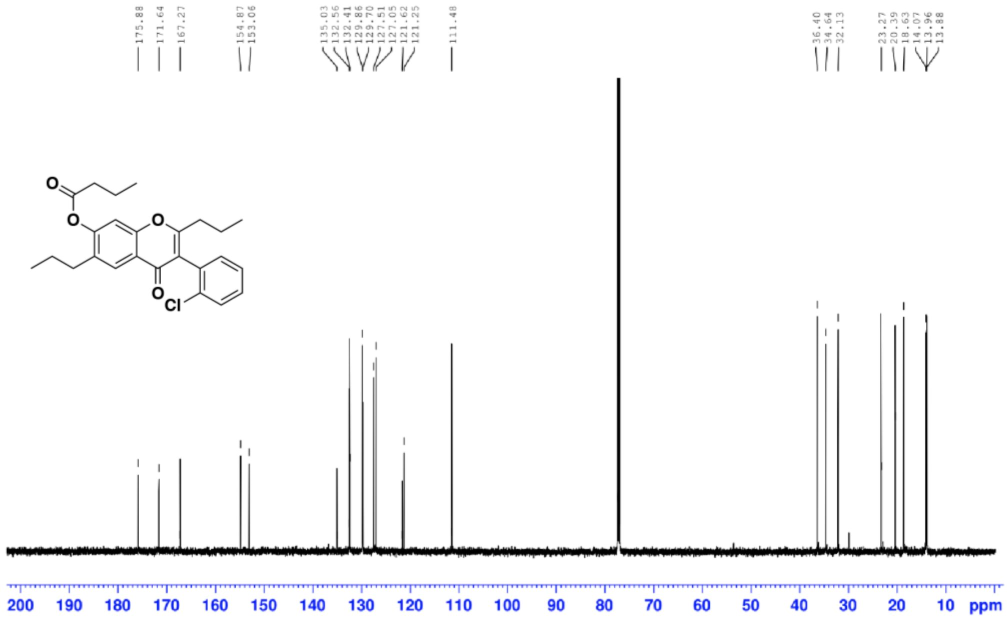
175.88
171.64
167.27
154.87
153.06
135.03
132.56
132.41
129.86
129.70
127.51
127.05
121.62
121.25
111.48
36.40
34.64
32.13
23.27
20.39
18.63
14.07
13.96
13.88
O
O
O
O
Cl
200 190 180 170 160 150 140 130 120 110 100 90 80 70 60 50 40 30 20 10 ppm

JG-3-030

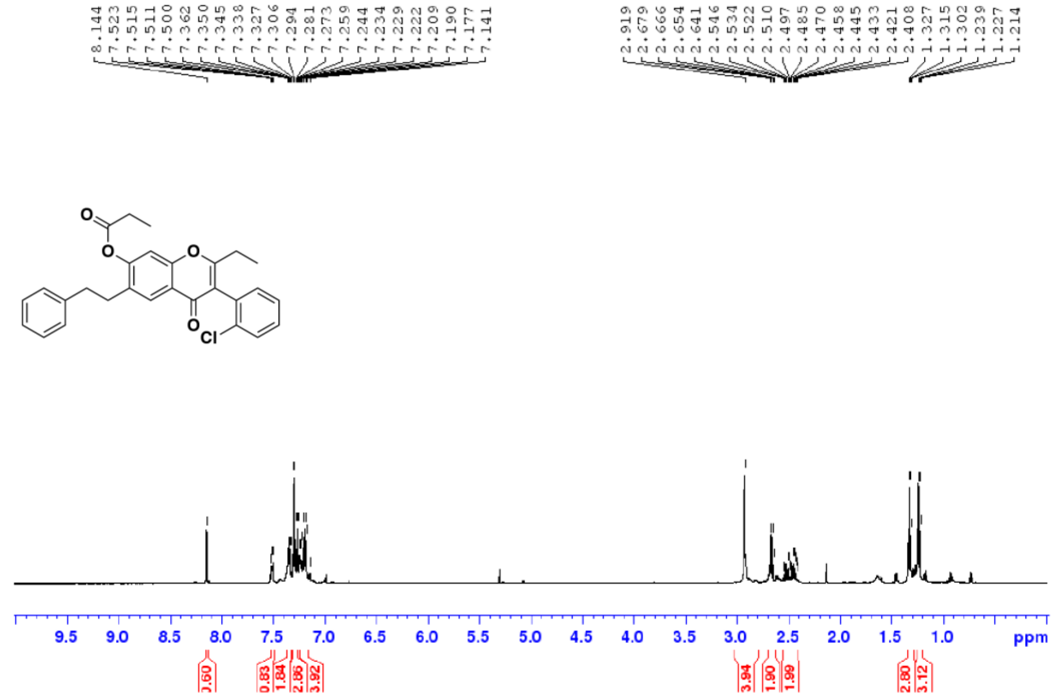

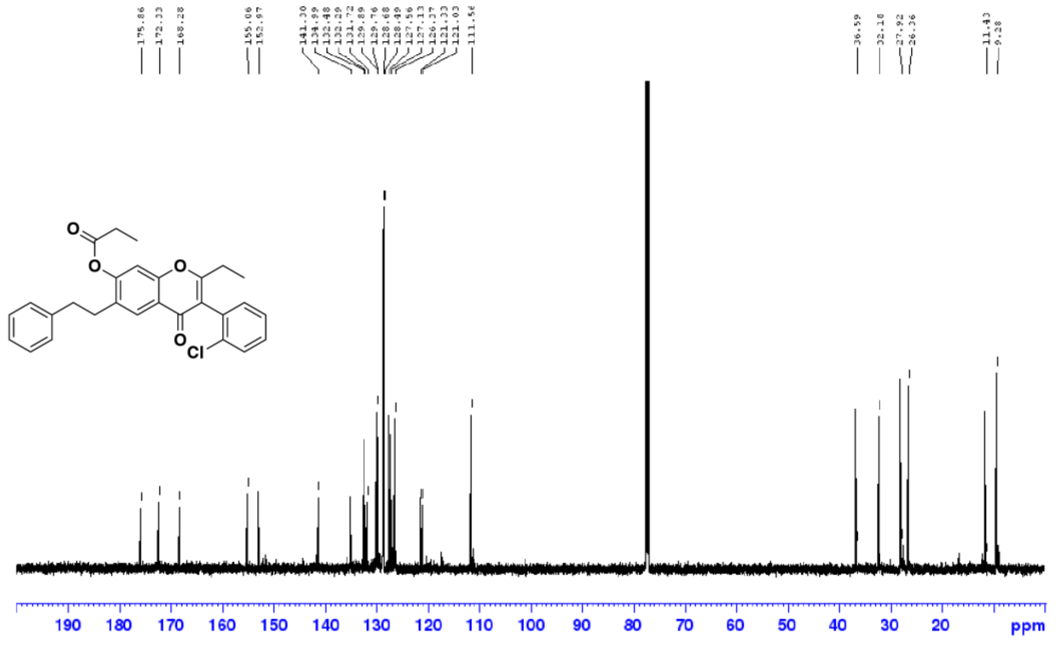

JG-3-031

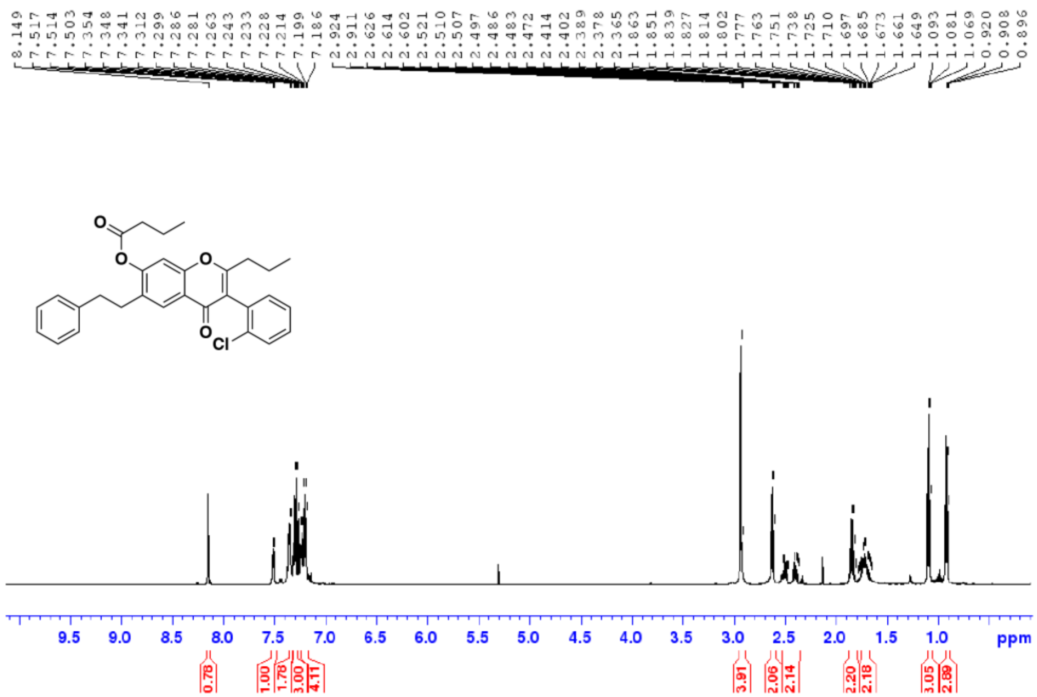
8.149 7.517 7.514 7.503 7.354 7.348 7.341 7.312 7.299 7.286 7.281 7.263 7.243 7.233 7.228 7.214 7.199 7.186 2.924 2.911 2.626 2.614 2.602 2.521 2.510 2.507 2.497 2.486 2.483 2.472 2.414 2.402 2.389 2.378 2.365 1.863 1.851 1.839 1.827 1.814 1.802 1.777 1.763 1.751 1.738 1.725 1.710 1.697 1.685 1.673 1.661 1.649 1.093 1.081 1.069 0.920 0.908 0.896
O
O
O
O
Cl
9.5 9.0 8.5 8.0 7.5 7.0 6.5 6.0 5.5 5.0 4.5 4.0 3.5 3.0 2.5 2.0 1.5 1.0 ppm
0.78 1.00 1.78 3.00 4.11 3.91 2.06 2.14 2.20 2.18 3.05 2.89

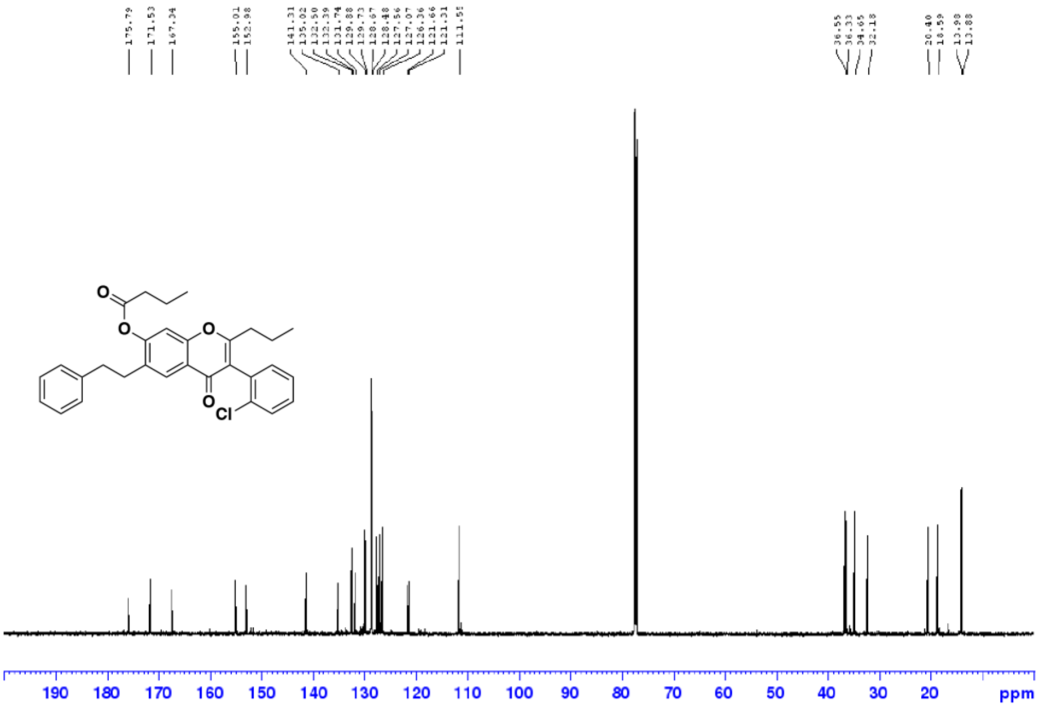
O
O
O
O
Cl
190 180 170 160 150 140 130 120 110 100 90 80 70 60 50 40 30 20 ppm

JG-3-034

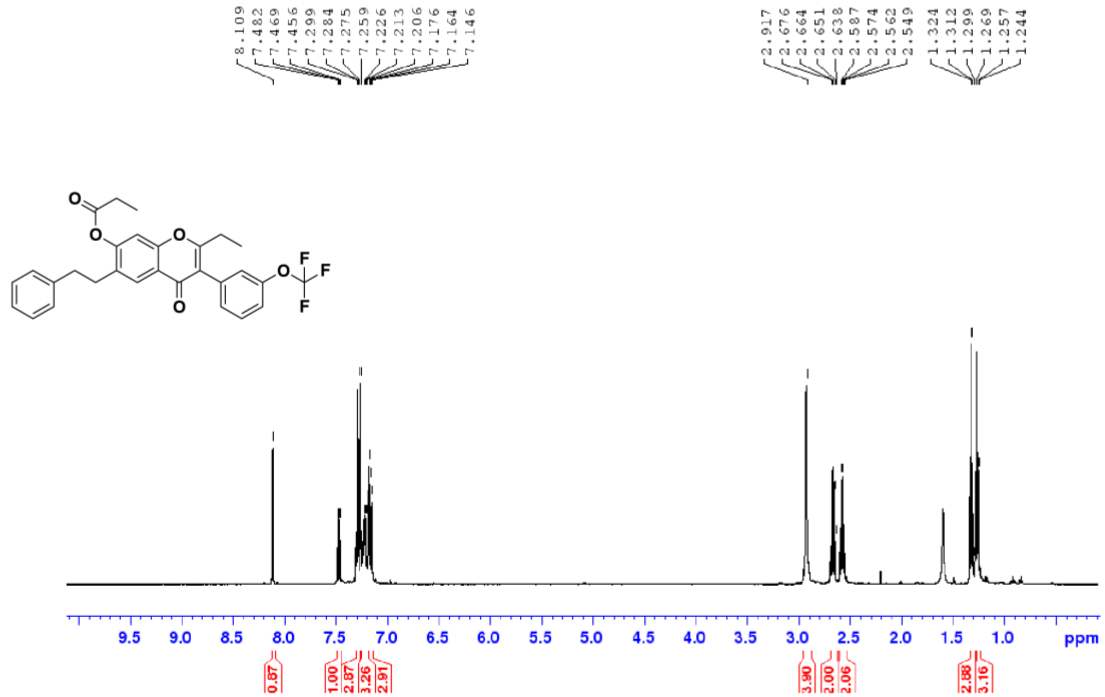

8.109
7.482
7.469
7.456
7.299
7.284
7.275
7.259
7.226
7.213
7.206
7.176
7.164
7.146
2.917
2.676
2.664
2.651
2.638
2.587
2.574
2.562
2.549
1.324
1.312
1.299
1.269
1.257
1.244
O
O
O
O
F
F
F
0.87
1.00
2.87
3.26
2.91
3.90
2.00
2.06
2.88
3.16
ppm


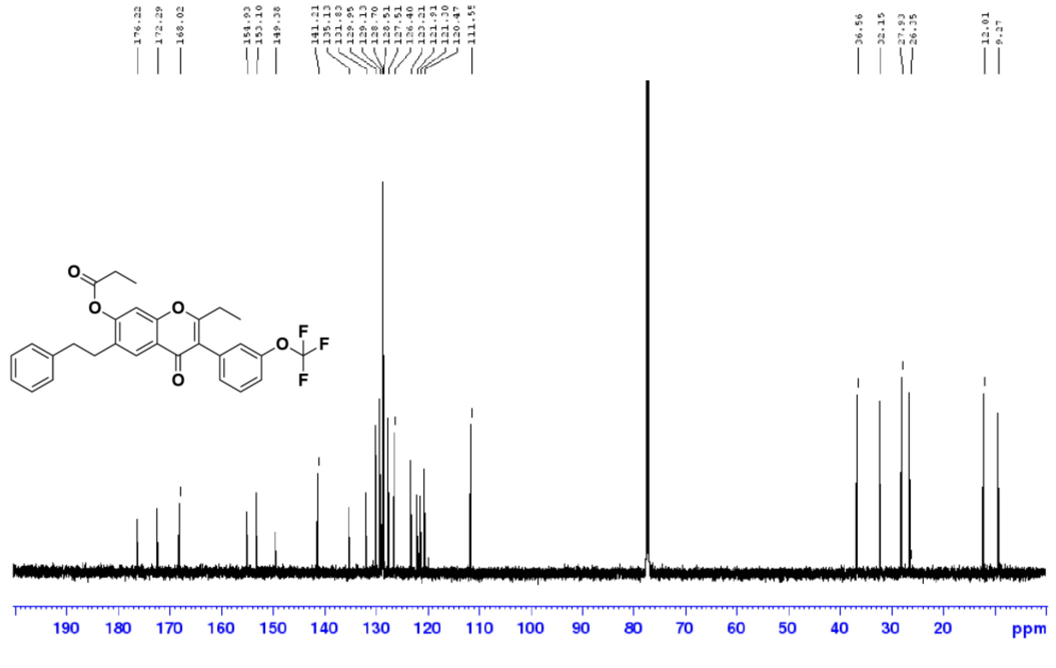

176.22
172.29
168.02
154.93
153.10
149.38
141.21
135.13
131.83
129.95
129.13
128.70
128.51
127.51
126.40
123.21
121.91
121.30
120.47
111.55
36.56
32.15
27.93
26.35
12.01
9.27
O
O
O
O
F
F
F
ppm

JG-3-035

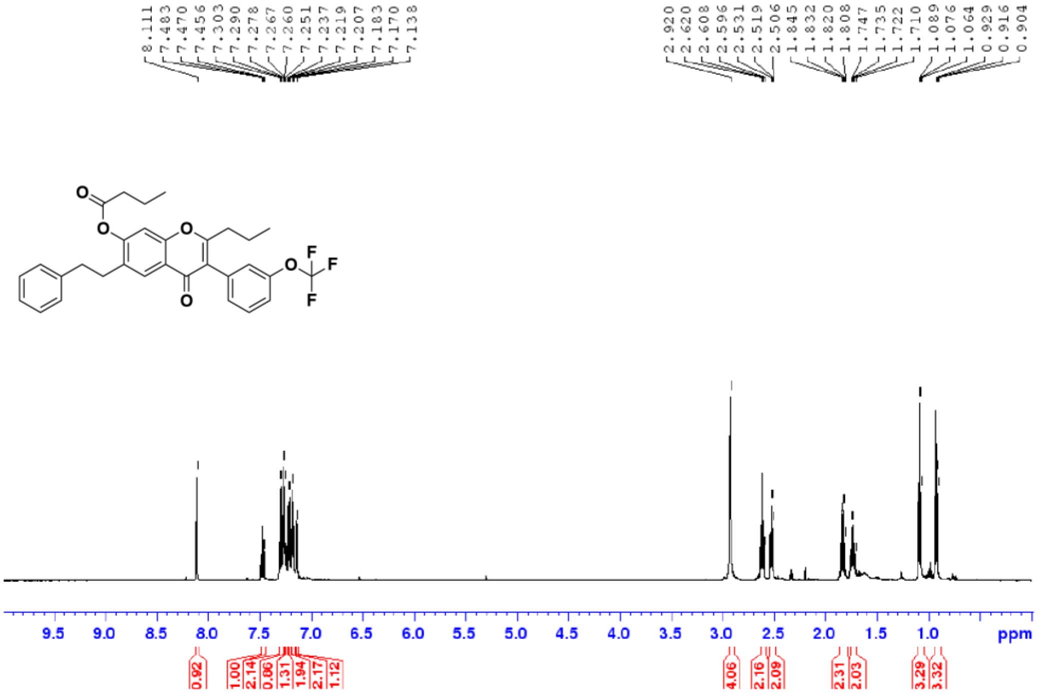
8.111 7.483 7.470 7.456 7.303 7.290 7.278 7.267 7.260 7.251 7.237 7.219 7.207 7.183 7.170 7.138
2.920 2.620 2.608 2.596 2.531 2.519 2.506 1.845 1.832 1.820 1.808 1.747 1.735 1.722 1.710 1.089 1.076 1.064 0.929 0.916 0.904
O
O
O
O
F
F
F
O
9.5 9.0 8.5 8.0 7.5 7.0 6.5 6.0 5.5 5.0 4.5 4.0 3.5 3.0 2.5 2.0 1.5 1.0 ppm
0.92 1.00 2.14 0.06 1.31 1.94 2.17 1.12 4.06 2.16 2.09 2.31 2.03 3.29 3.32

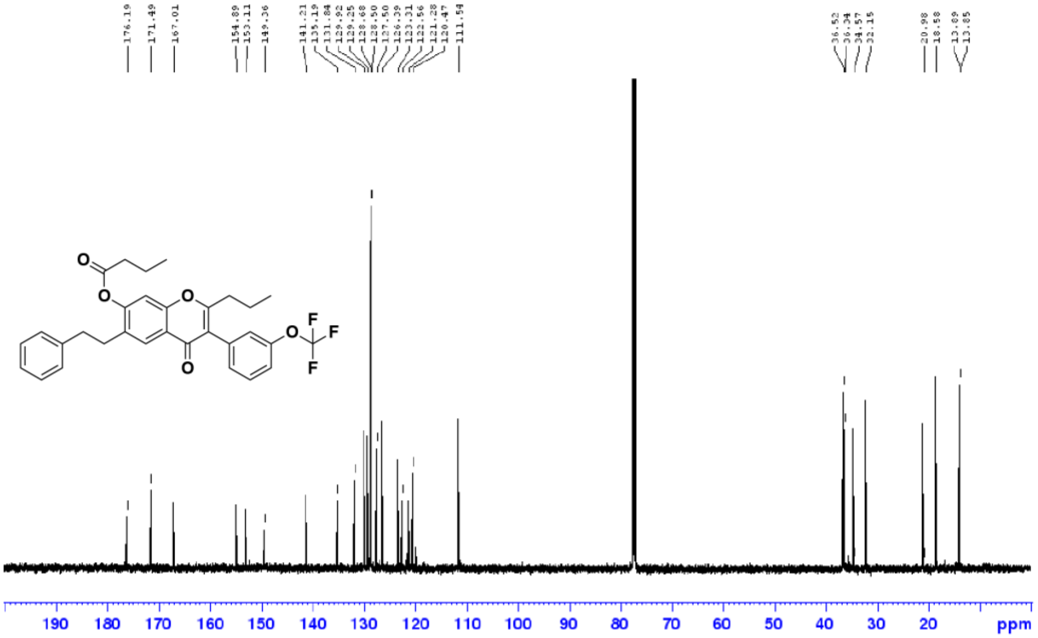
176.19 171.49 167.01 154.89 153.11 149.36 141.21 135.19 131.84 129.92 129.25 128.68 128.50 127.50 126.39 123.31 122.56 121.28 120.47 111.54
36.52 36.34 34.57 32.15 20.98 18.58 13.89 13.85
O
O
O
O
F
F
F
O
190 180 170 160 150 140 130 120 110 100 90 80 70 60 50 40 30 20 ppm

JG-3-037

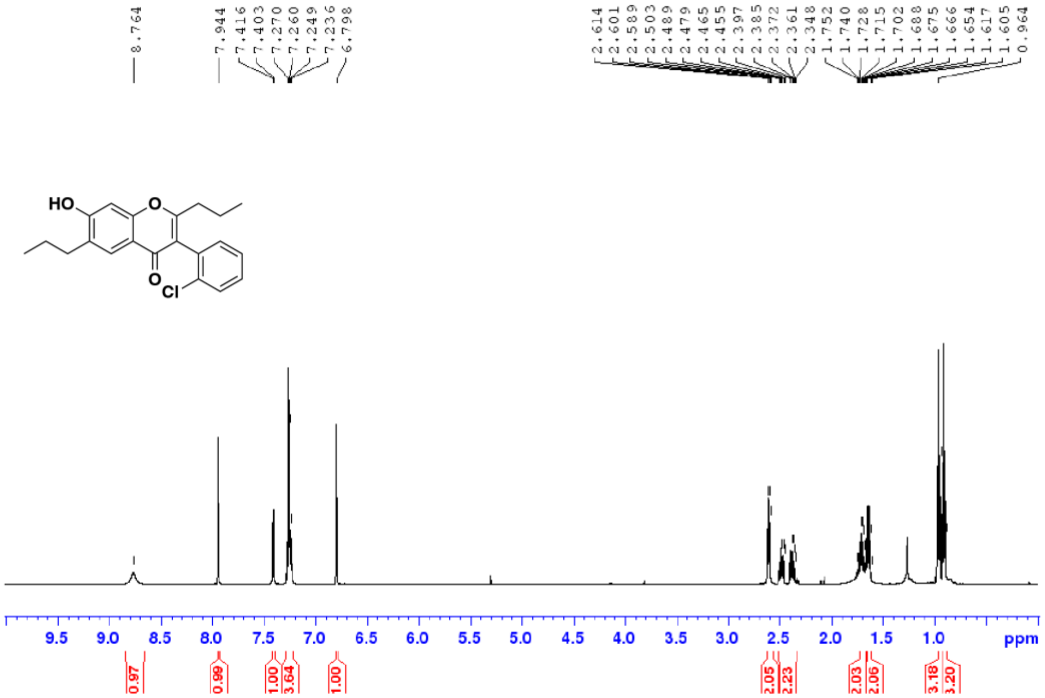
8.764
7.944
7.416
7.403
7.270
7.260
7.249
7.236
6.798
2.614
2.601
2.589
2.503
2.489
2.479
2.465
2.455
2.397
2.385
2.372
2.361
2.348
1.752
1.740
1.728
1.715
1.702
1.688
1.675
1.666
1.654
1.617
1.605
0.964
HO
O
Cl
9.5
9.0
8.5
8.0
7.5
7.0
6.5
6.0
5.5
5.0
4.5
4.0
3.5
3.0
2.5
2.0
1.5
1.0
ppm
0.97
0.99
1.00
3.64
1.00
2.05
2.23
2.03
2.06
3.18
3.20

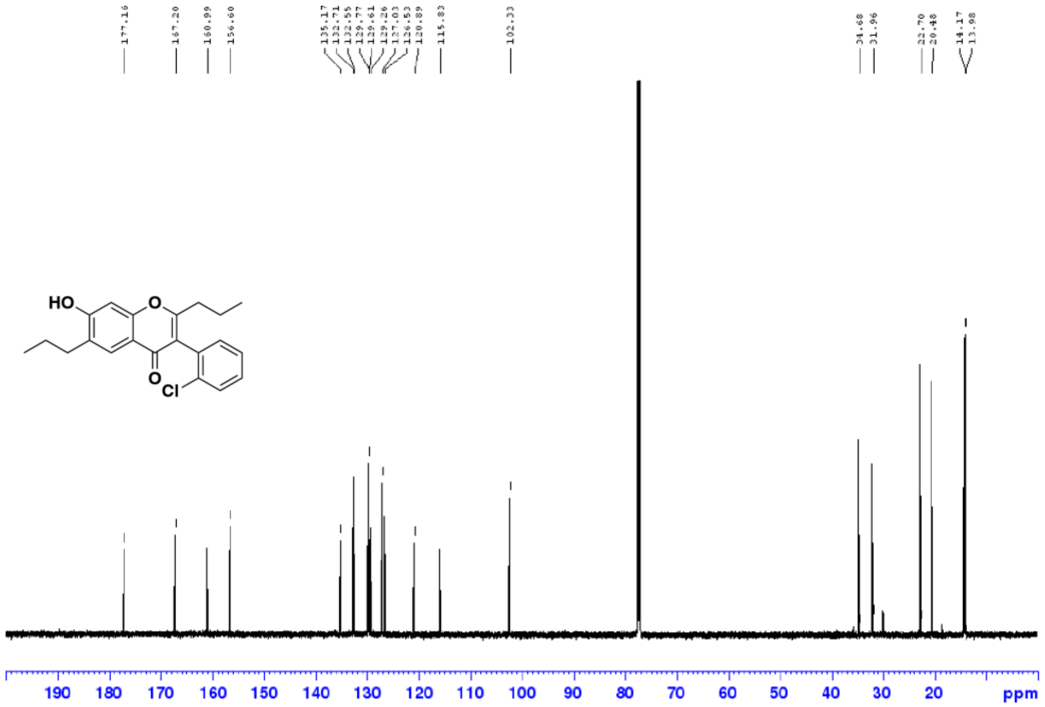
177.16
167.20
160.99
156.60
135.17
132.71
132.55
129.77
129.61
129.26
127.03
126.53
120.89
115.83
102.33
34.68
31.96
22.70
20.48
14.17
13.98
HO
O
Cl
190
180
170
160
150
140
130
120
110
100
90
80
70
60
50
40
30
20
ppm

JG-3-038

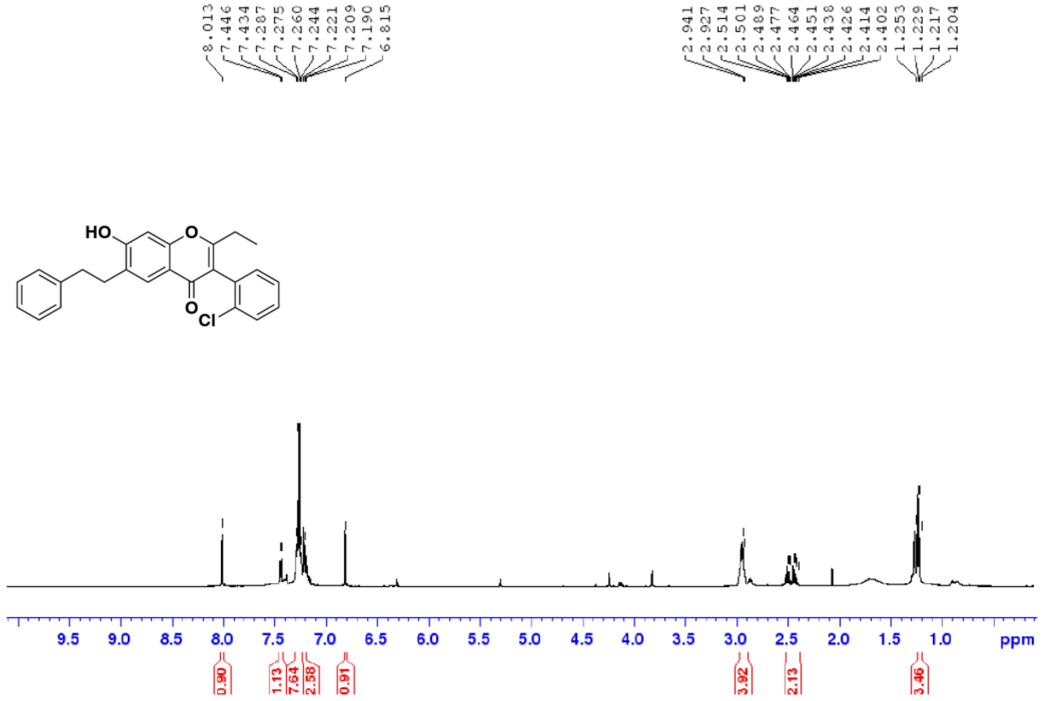
8.013
7.446
7.434
7.287
7.275
7.260
7.244
7.221
7.209
7.190
6.815
2.941
2.927
2.514
2.501
2.489
2.477
2.464
2.451
2.438
2.426
2.414
2.402
1.253
1.229
1.217
1.204
HO
O
O
Cl
9.5 9.0 8.5 8.0 7.5 7.0 6.5 6.0 5.5 5.0 4.5 4.0 3.5 3.0 2.5 2.0 1.5 1.0 ppm
0.90
1.13
7.64
2.58
0.91
3.92
2.13
3.46

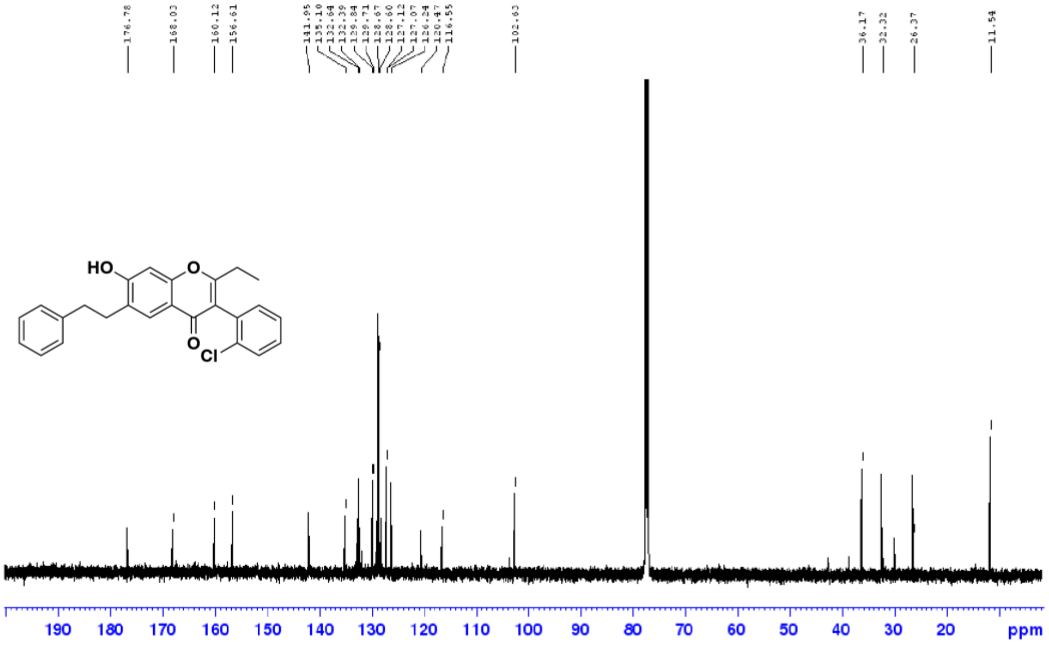
176.78
168.03
160.12
156.61
141.95
135.10
132.64
132.39
129.84
129.71
128.67
128.60
127.12
127.07
126.24
120.47
116.55
102.63
36.17
32.32
26.37
11.54
HO
O
O
Cl
190 180 170 160 150 140 130 120 110 100 90 80 70 60 50 40 30 20 ppm

JG-3-039

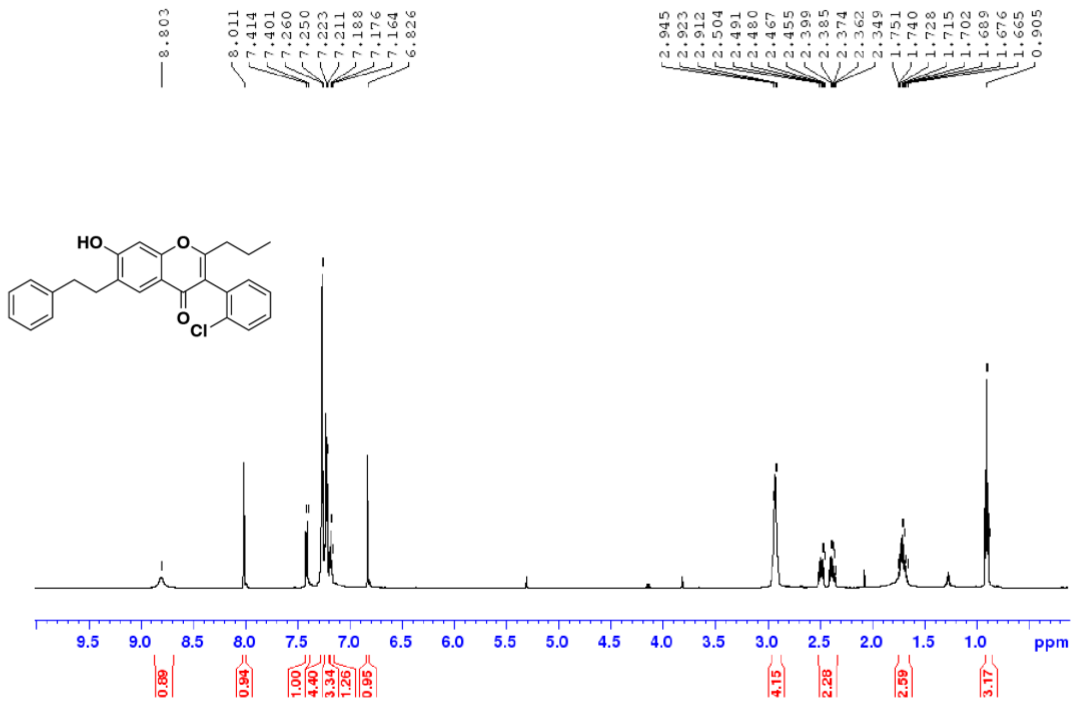
8.803
8.011
7.414
7.401
7.260
7.250
7.223
7.211
7.188
7.176
7.164
6.826
2.945
2.923
2.912
2.504
2.491
2.480
2.467
2.455
2.399
2.385
2.374
2.362
2.349
1.751
1.740
1.728
1.715
1.702
1.689
1.676
1.665
0.905
HO
O
O
Cl
9.5 9.0 8.5 8.0 7.5 7.0 6.5 6.0 5.5 5.0 4.5 4.0 3.5 3.0 2.5 2.0 1.5 1.0 ppm
0.89
0.94
1.00
4.40
3.34
1.26
0.95
4.15
2.28
2.59
3.17

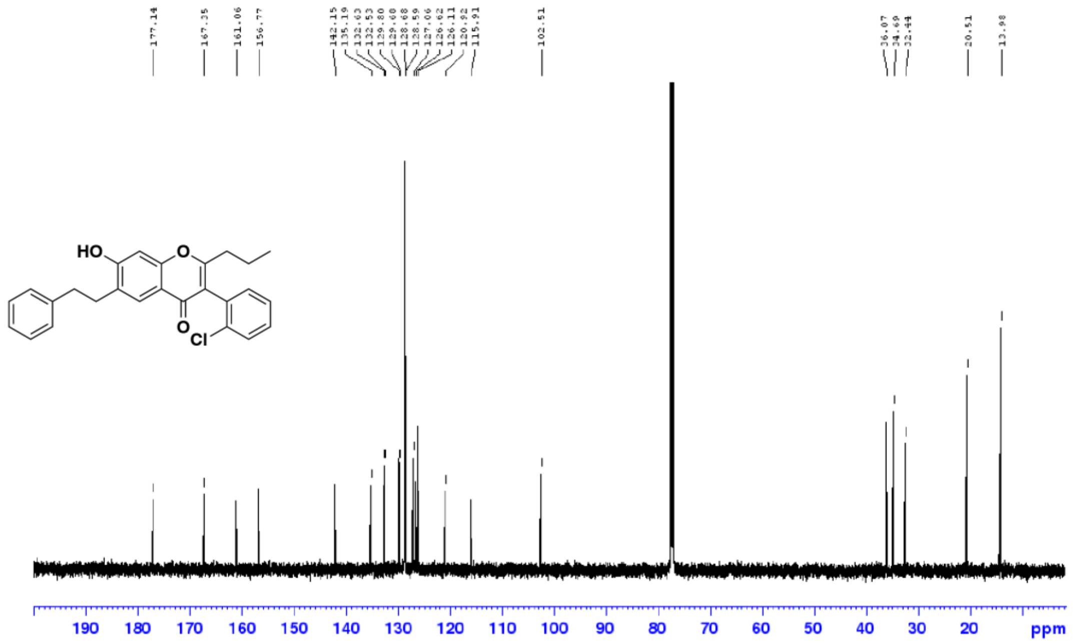
177.14
167.35
161.06
156.77
142.15
135.19
132.63
132.53
129.80
129.68
128.68
128.59
127.06
126.62
126.11
120.92
115.91
102.51
36.07
34.69
32.44
20.51
13.98
HO
O
O
Cl
190 180 170 160 150 140 130 120 110 100 90 80 70 60 50 40 30 20 ppm

JG-3-041

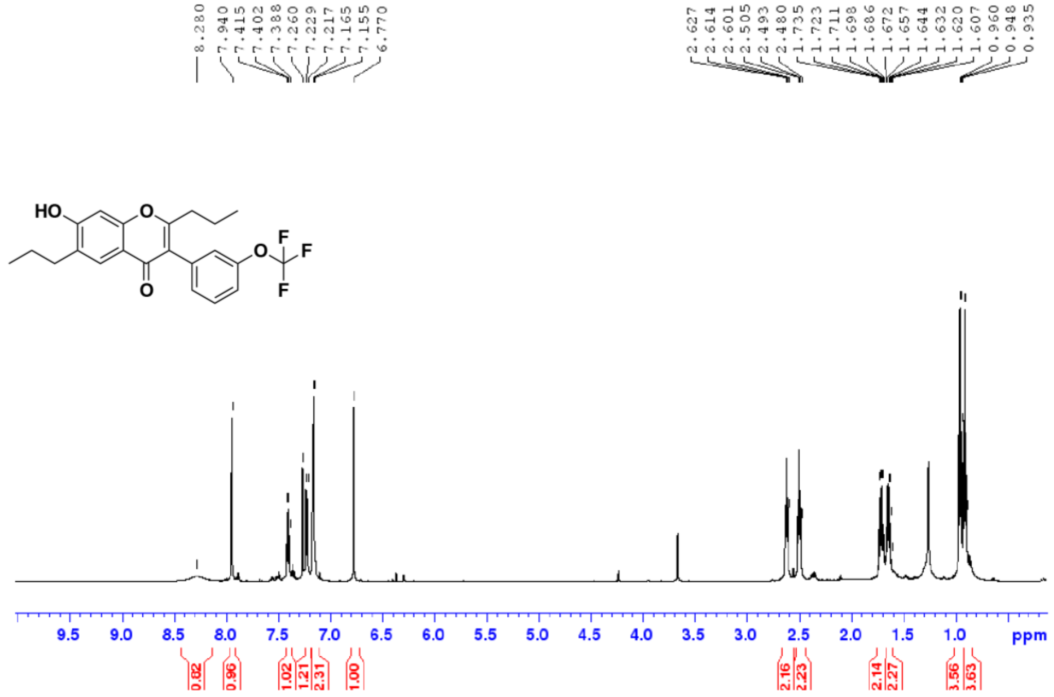
8.280
7.940
7.415
7.402
7.388
7.260
7.229
7.217
7.165
7.155
6.770
2.627
2.614
2.601
2.505
2.493
2.480
1.735
1.723
1.711
1.698
1.686
1.672
1.657
1.644
1.632
1.620
1.607
0.960
0.948
0.935
HO
O
F
F
F
O
ppm
0.82
0.96
1.02
1.21
2.31
1.00
2.16
2.23
2.14
2.27
3.56
3.63

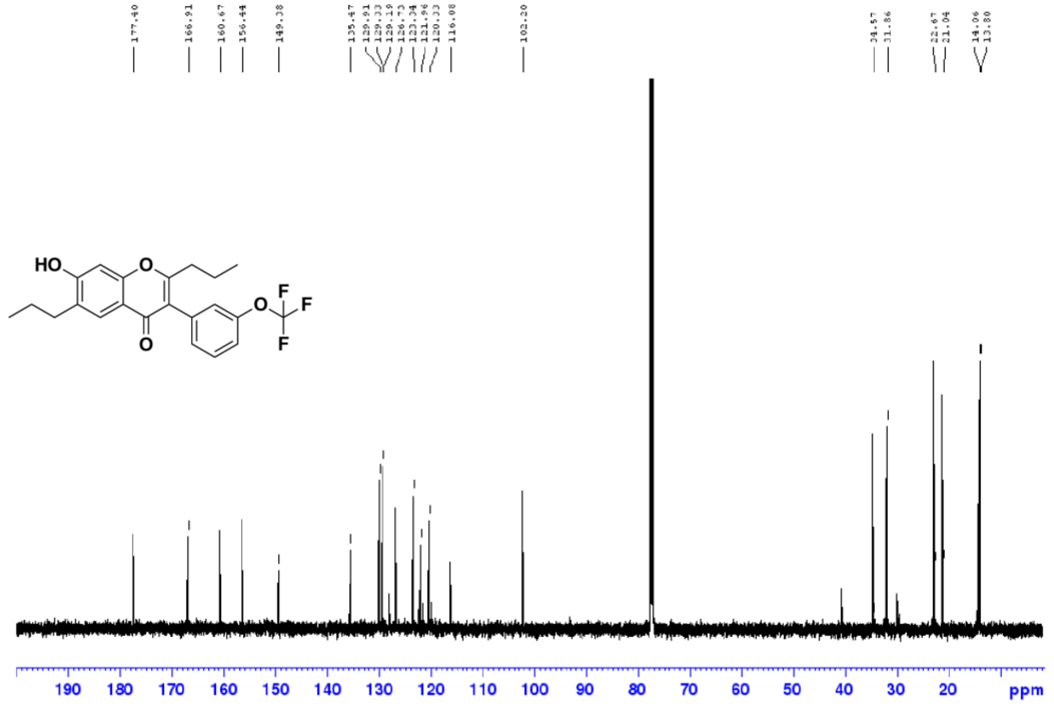
177.40
166.91
160.67
156.44
149.38
135.47
129.91
129.33
128.19
126.73
123.34
121.96
120.33
116.08
102.20
34.57
31.86
22.67
21.04
14.06
13.80
HO
O
F
F
F
O
ppm

JG-3-042

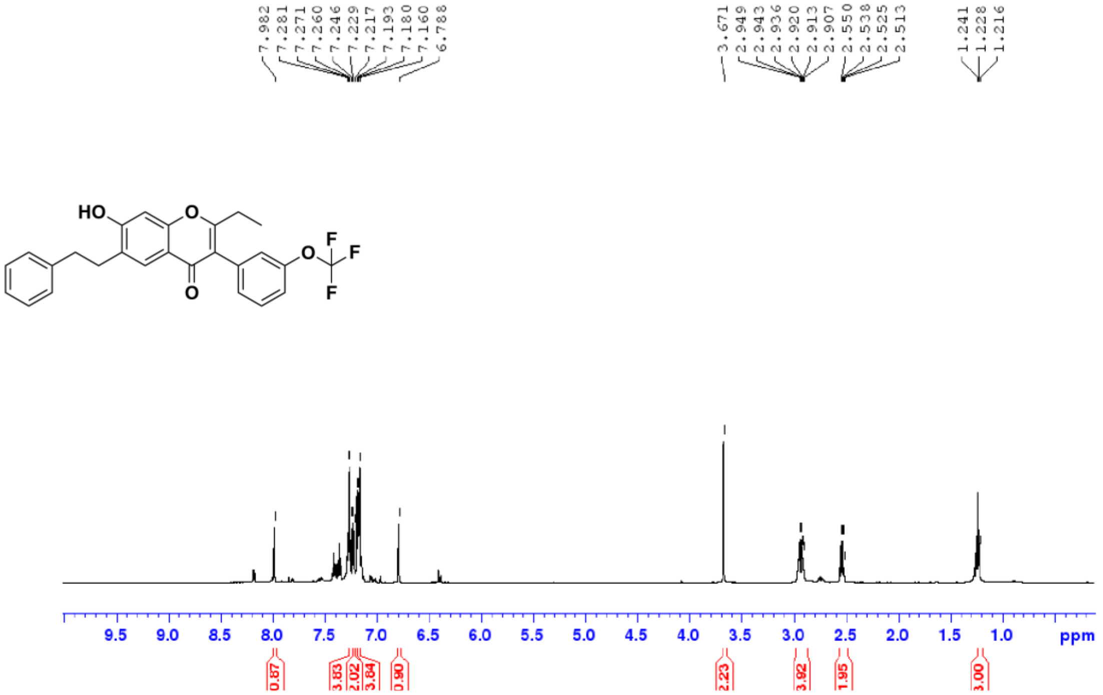

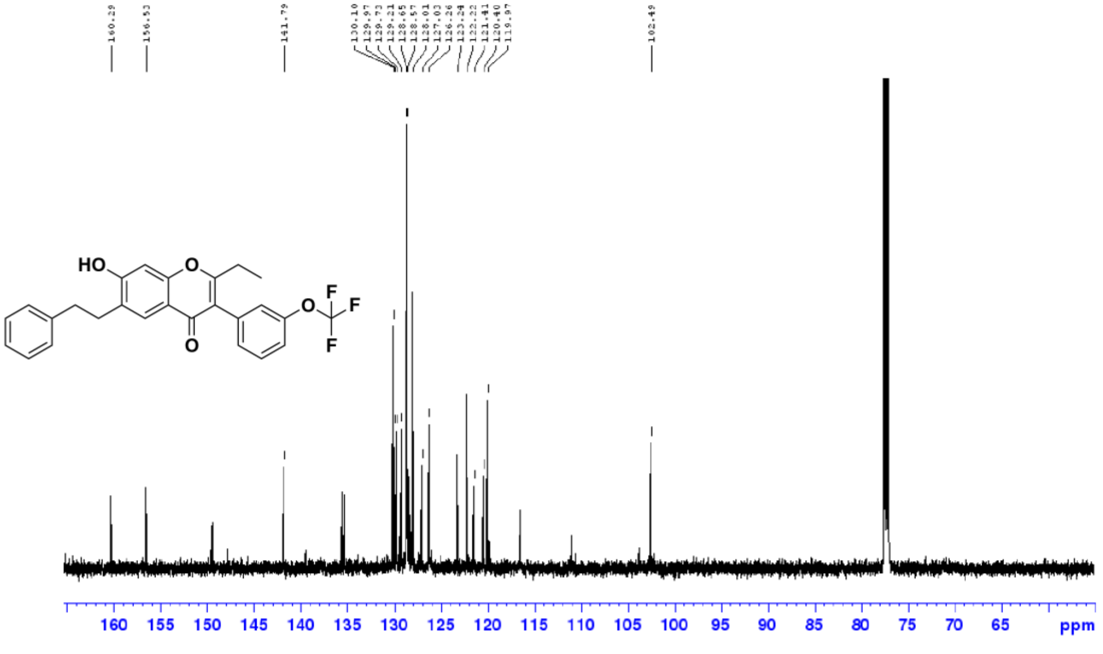

JG-3-043

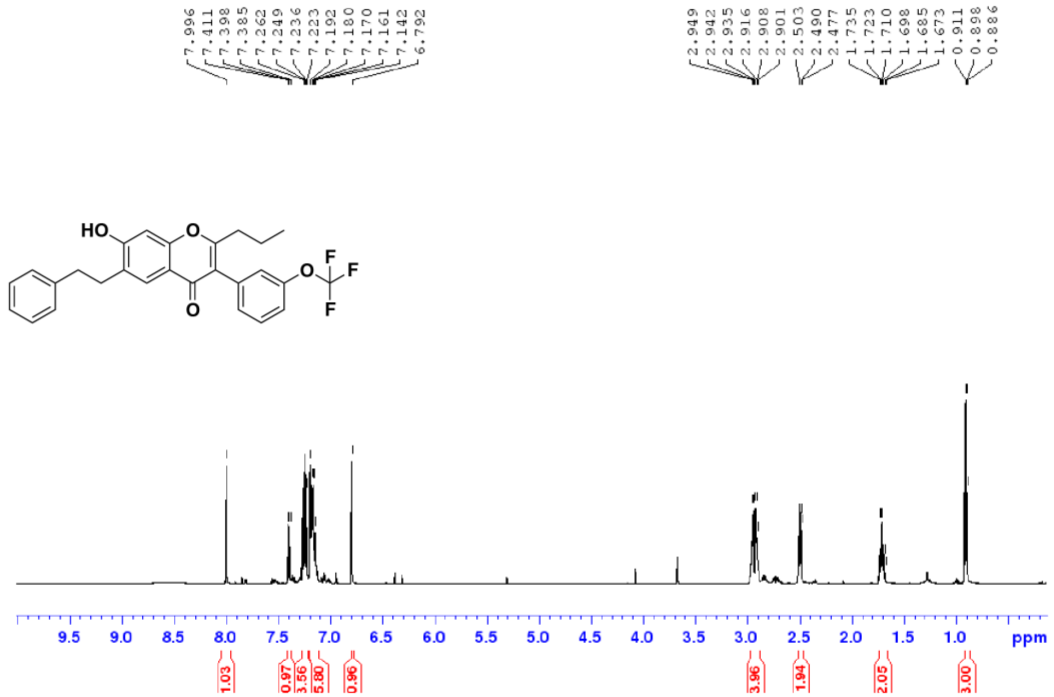
7.996
7.411
7.398
7.385
7.262
7.249
7.236
7.223
7.192
7.180
7.170
7.161
7.142
6.792
2.949
2.942
2.935
2.916
2.908
2.901
2.503
2.490
2.477
1.735
1.723
1.710
1.698
1.685
1.673
0.911
0.898
0.886
HO
O
O
F
F
F
9.5 9.0 8.5 8.0 7.5 7.0 6.5 6.0 5.5 5.0 4.5 4.0 3.5 3.0 2.5 2.0 1.5 1.0 ppm
1.03
0.97
3.56
5.80
0.96
3.96
1.94
2.05
3.00

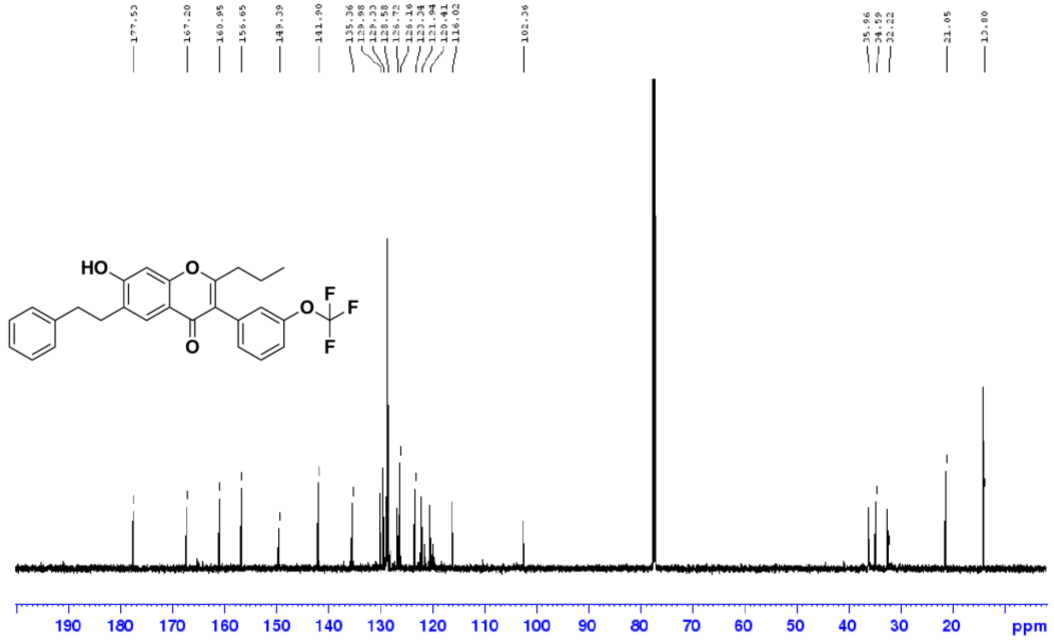
177.53
167.20
160.95
156.65
149.39
141.90
135.36
129.98
129.33
128.58
126.72
126.16
123.34
121.94
120.41
116.02
102.36
35.96
34.59
32.22
21.05
13.80
HO
O
O
F
F
F
190 180 170 160 150 140 130 120 110 100 90 80 70 60 50 40 30 20 ppm

JG-3-044

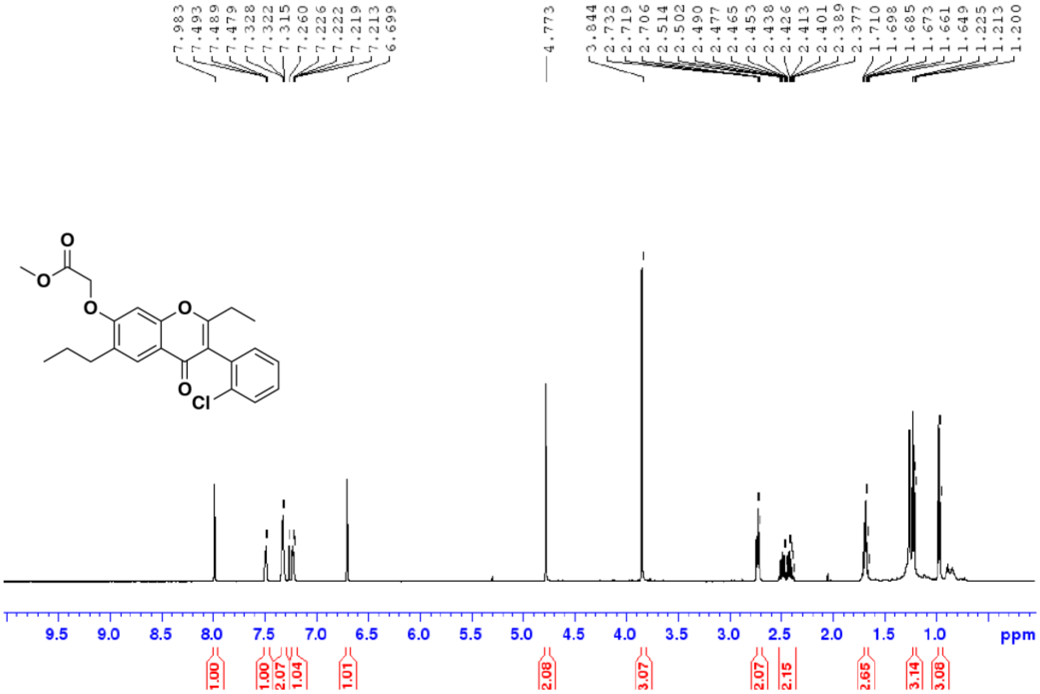
7.983
7.493
7.489
7.479
7.328
7.322
7.315
7.260
7.226
7.222
7.219
7.213
6.699
4.773
3.844
2.732
2.719
2.706
2.514
2.502
2.490
2.477
2.465
2.453
2.438
2.426
2.413
2.401
2.389
2.377
1.710
1.698
1.685
1.673
1.661
1.649
1.225
1.213
1.200
O
O
O
O
O
Cl
9.5 9.0 8.5 8.0 7.5 7.0 6.5 6.0 5.5 5.0 4.5 4.0 3.5 3.0 2.5 2.0 1.5 1.0 ppm
1.00
1.00
2.07
1.04
1.01
2.08
3.07
2.07
2.15
2.65
3.14
3.08

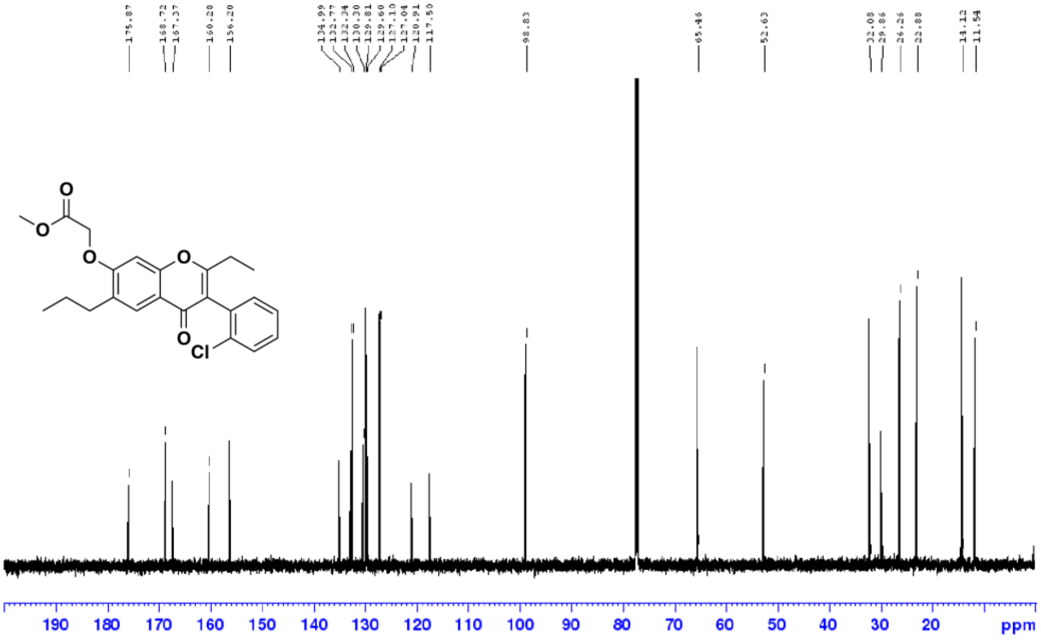
175.87
168.72
167.37
160.28
156.20
134.99
132.77
132.34
130.30
129.81
129.60
127.10
127.04
120.91
117.50
98.83
65.46
52.63
32.08
29.86
26.26
22.88
14.12
11.54
O
O
O
O
O
Cl
190 180 170 160 150 140 130 120 110 100 90 80 70 60 50 40 30 20 ppm

JG-3-045

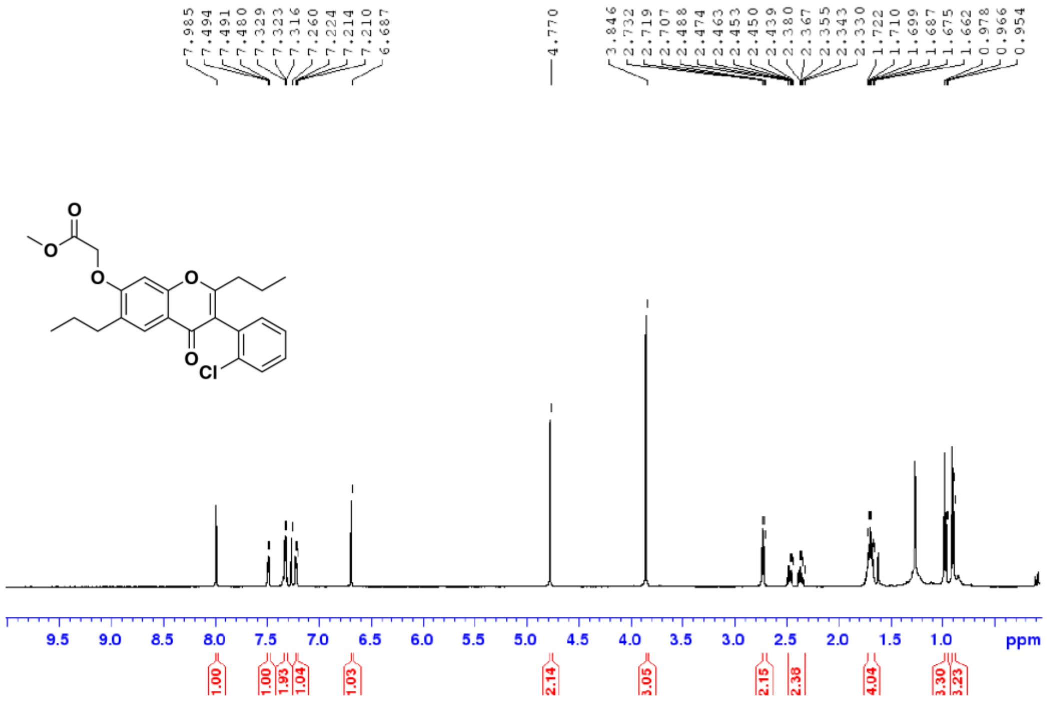

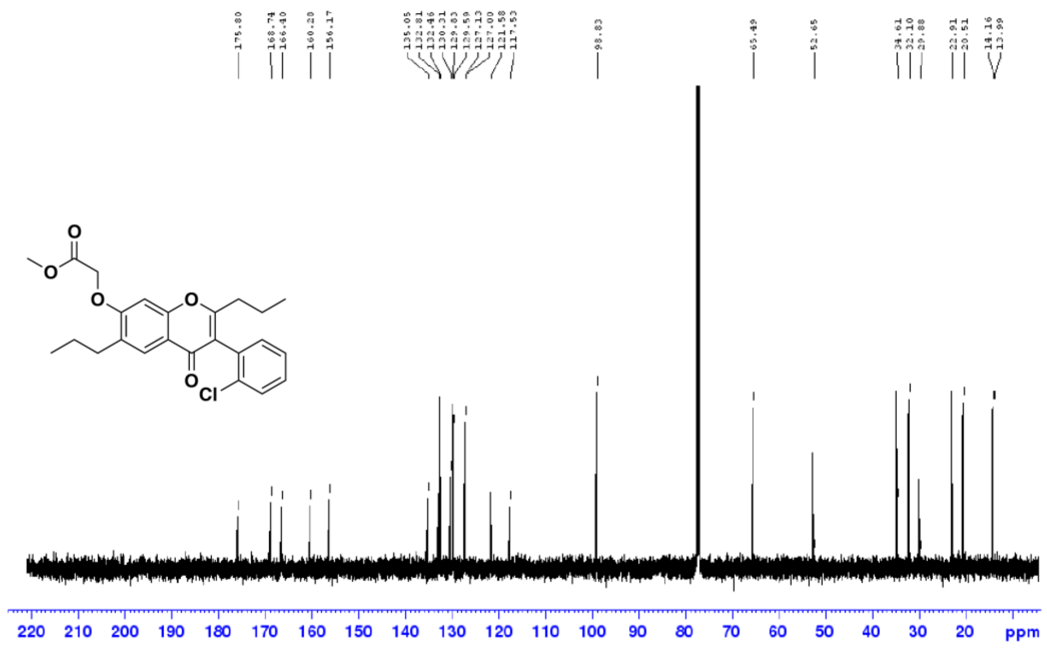

JG-3-046

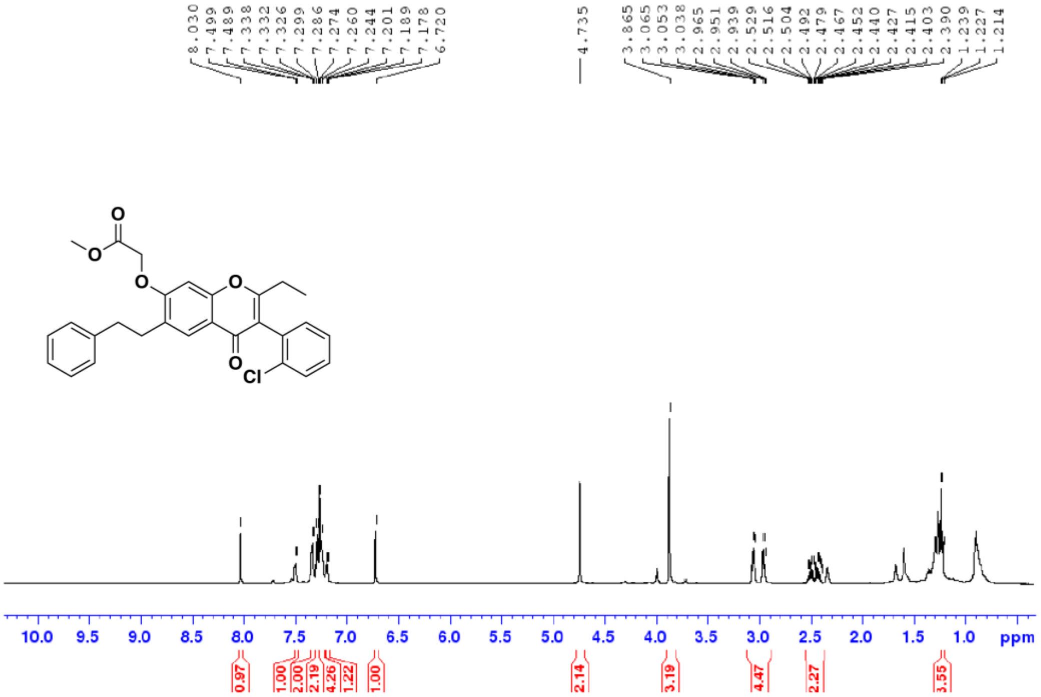
8.030
7.499
7.489
7.338
7.332
7.326
7.299
7.286
7.274
7.260
7.244
7.201
7.189
7.178
6.720
4.735
3.865
3.065
3.053
3.038
2.965
2.951
2.939
2.529
2.516
2.504
2.492
2.479
2.467
2.452
2.440
2.427
2.415
2.403
2.390
1.239
1.227
1.214
O
O
O
O
O
O
Cl
10.0 9.5 9.0 8.5 8.0 7.5 7.0 6.5 6.0 5.5 5.0 4.5 4.0 3.5 3.0 2.5 2.0 1.5 1.0 ppm
0.97
1.00
2.00
2.19
4.26
1.22
1.00
2.14
3.19
4.47
2.27
3.55

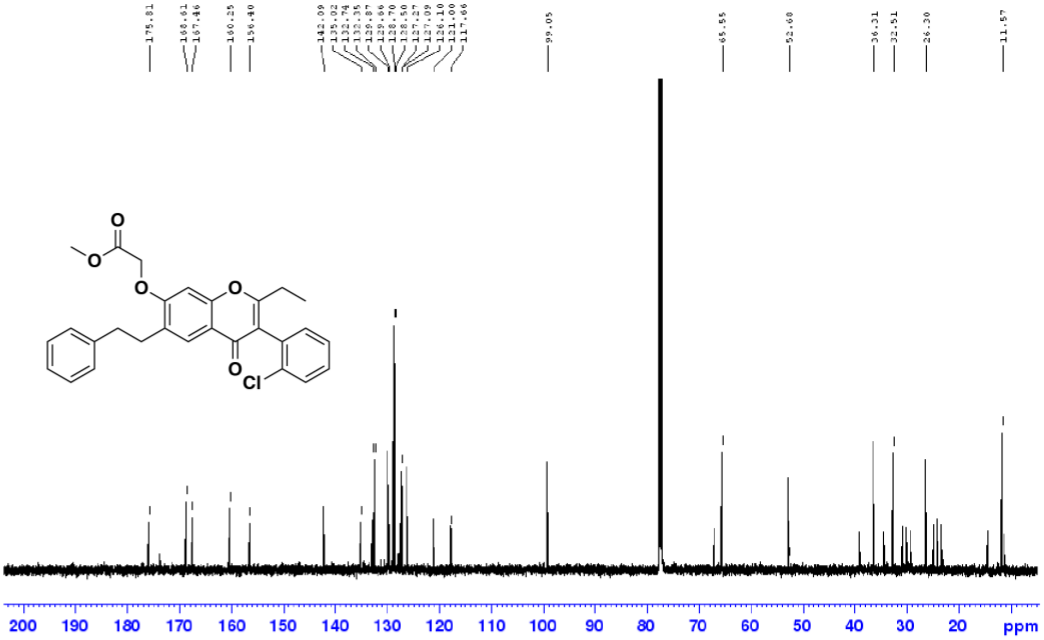
175.81
168.61
167.46
160.25
156.40
142.09
135.02
132.74
132.35
129.87
129.66
128.70
128.50
127.27
127.09
126.10
121.00
117.66
99.05
65.55
52.60
36.31
32.51
26.30
11.57
O
O
O
O
O
O
Cl
200 190 180 170 160 150 140 130 120 110 100 90 80 70 60 50 40 30 20 ppm

JG-3-047

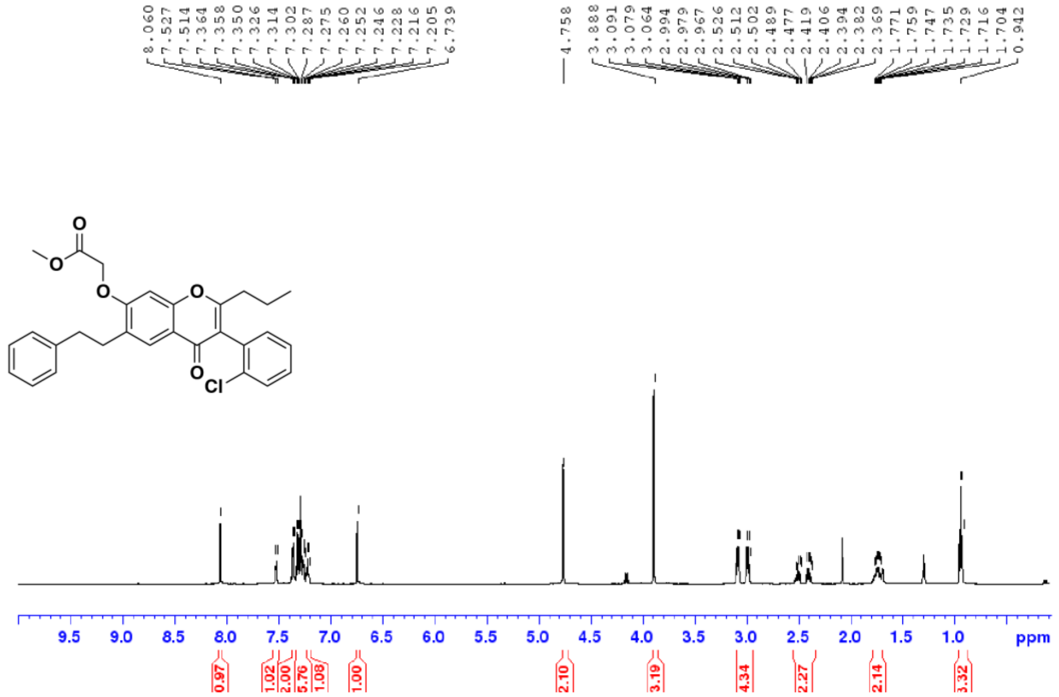

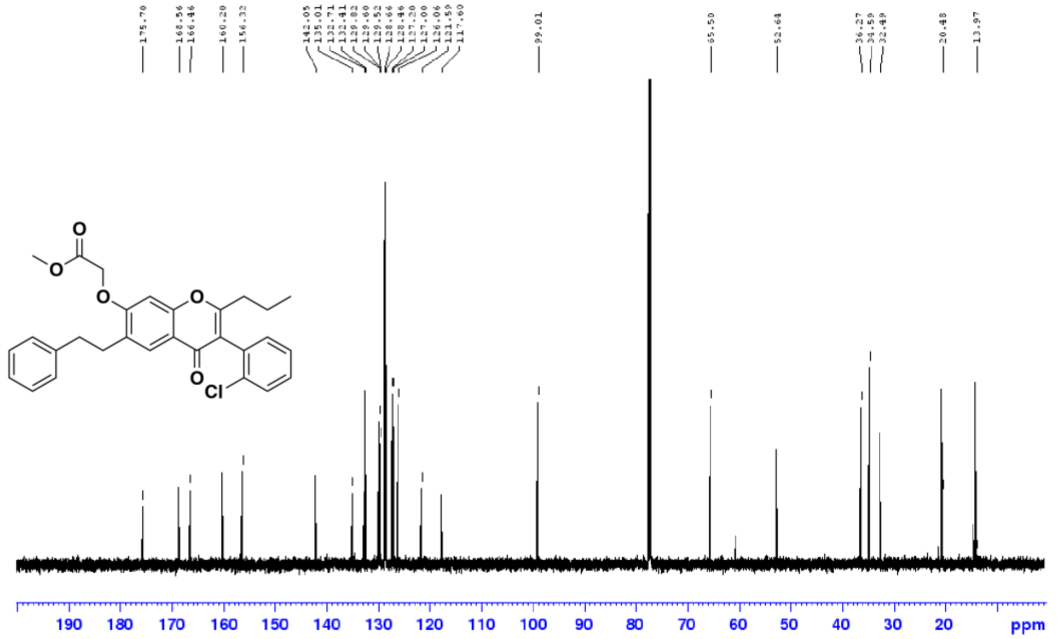

JG-3-048

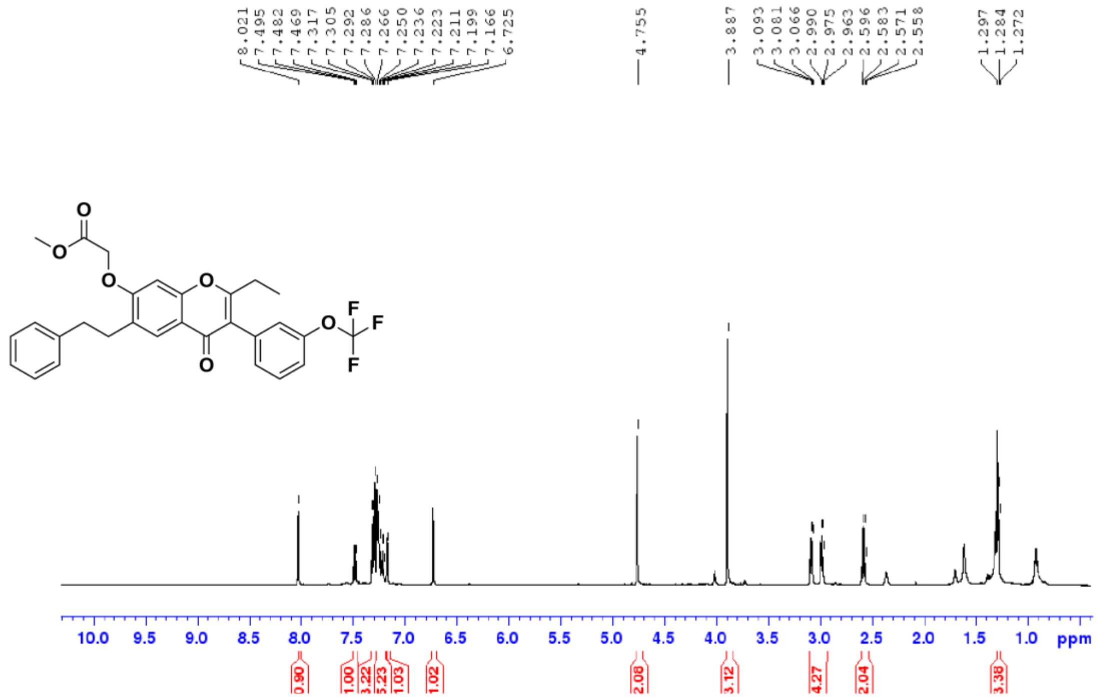
8.021
7.495
7.482
7.469
7.317
7.305
7.292
7.286
7.266
7.250
7.236
7.223
7.211
7.199
7.166
6.725
4.755
3.887
3.093
3.081
3.066
2.990
2.975
2.963
2.596
2.583
2.571
2.558
1.297
1.284
1.272
O
O
O
O
O
O
F
F
F
10.0 9.5 9.0 8.5 8.0 7.5 7.0 6.5 6.0 5.5 5.0 4.5 4.0 3.5 3.0 2.5 2.0 1.5 1.0 ppm
0.90
1.00
3.22
5.23
1.03
1.02
2.08
3.12
4.27
2.04
3.38

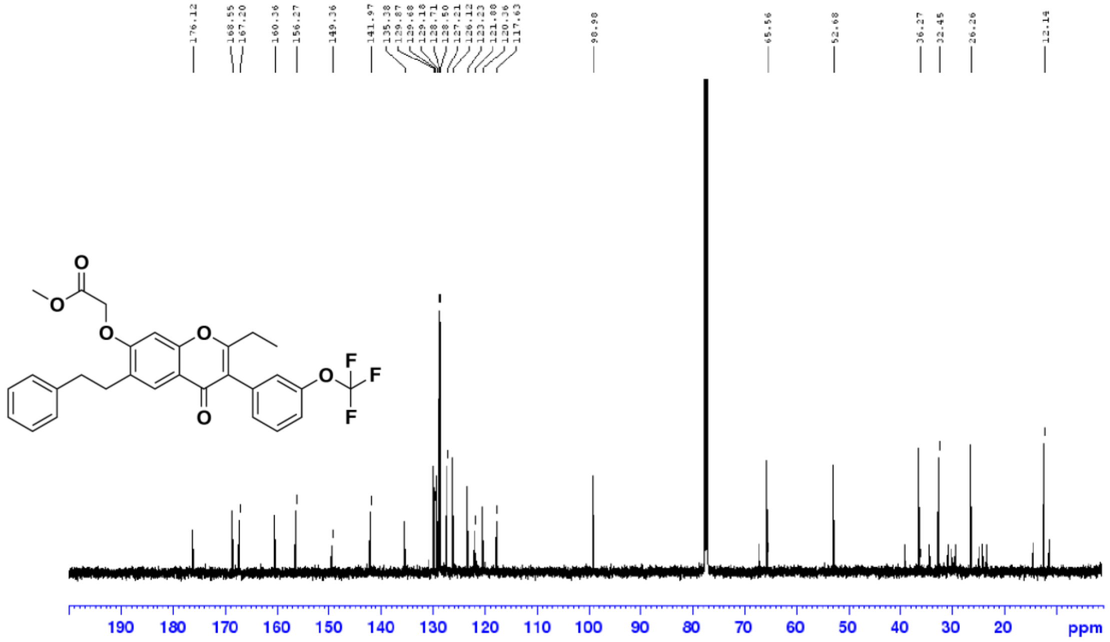
176.12
168.55
167.20
160.36
156.27
149.36
141.97
135.38
129.87
129.68
129.18
128.71
128.50
127.21
126.12
123.23
121.88
120.36
117.63
98.98
65.56
52.68
36.27
32.45
26.26
12.14
O
O
O
O
O
O
F
F
F
190 180 170 160 150 140 130 120 110 100 90 80 70 60 50 40 30 20 ppm

JG-3-049

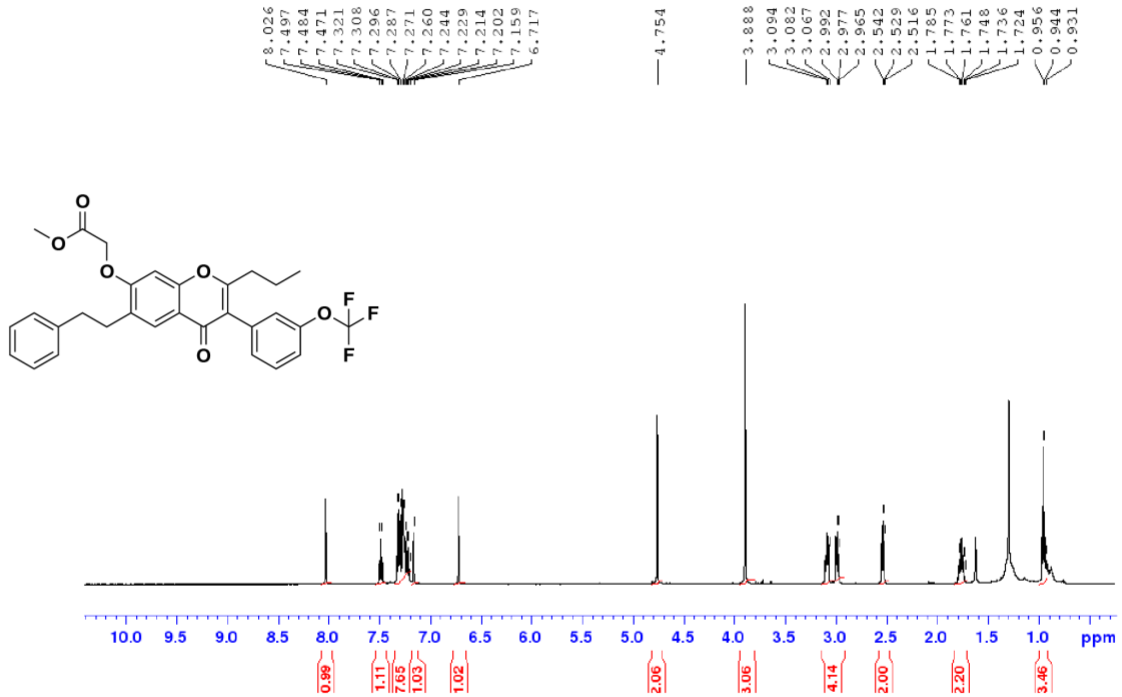

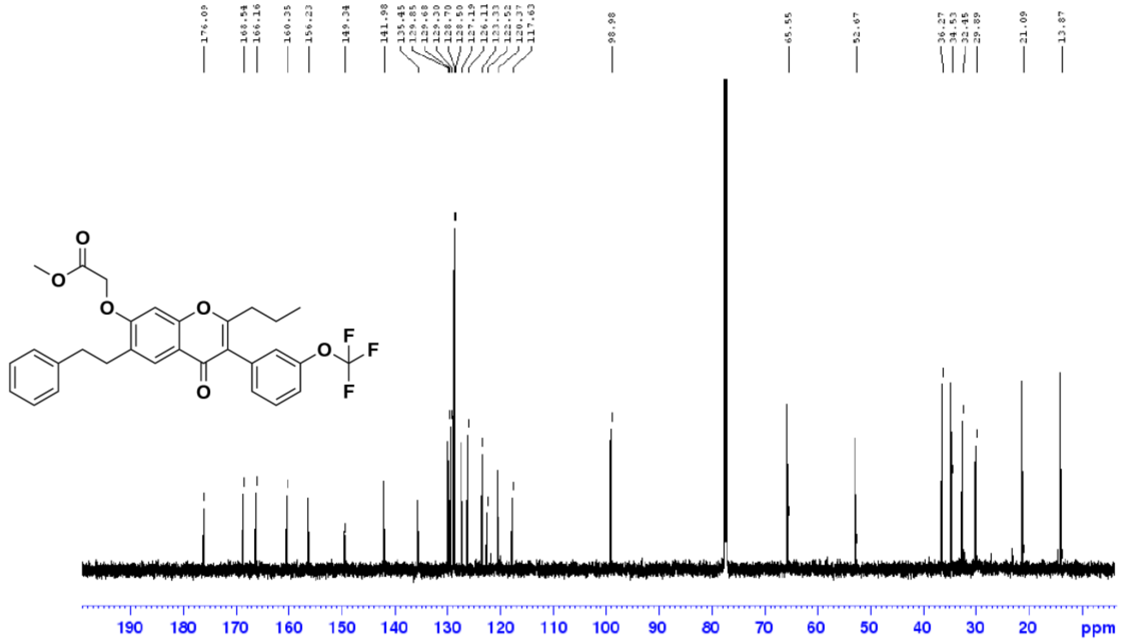

JG-3-119

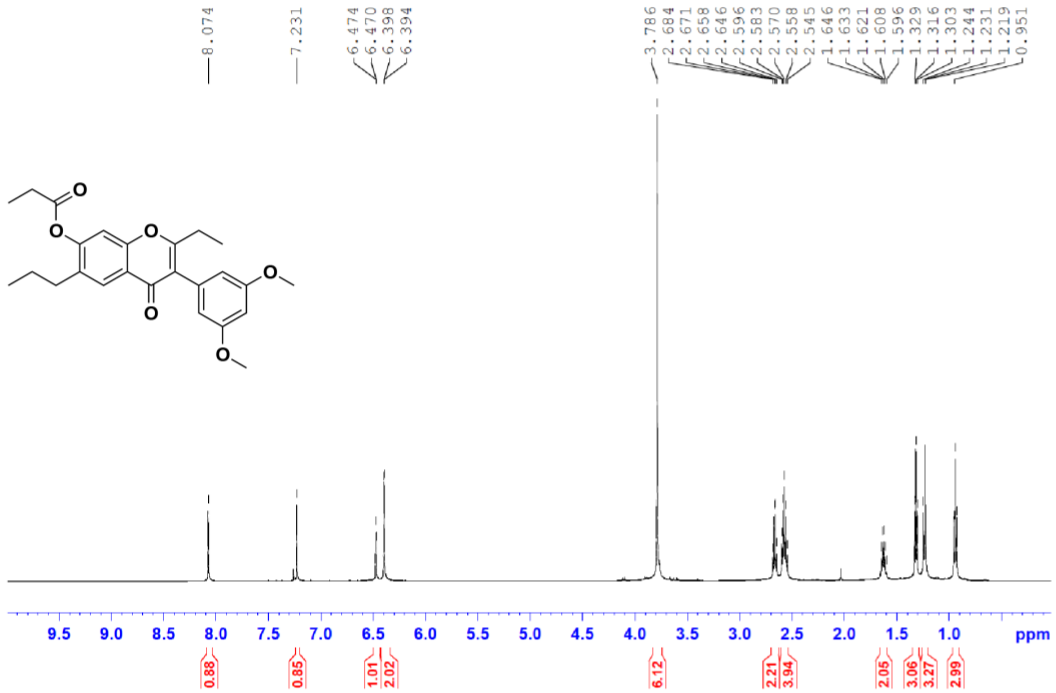
8.074
7.231
6.474
6.470
6.398
6.394
3.786
2.684
2.671
2.658
2.646
2.596
2.583
2.570
2.558
2.545
1.646
1.633
1.621
1.608
1.596
1.329
1.316
1.303
1.244
1.231
1.219
0.951
9.5 9.0 8.5 8.0 7.5 7.0 6.5 6.0 5.5 5.0 4.5 4.0 3.5 3.0 2.5 2.0 1.5 1.0 ppm
0.88
0.85
1.01
2.02
6.12
2.21
3.94
2.05
3.06
3.27
2.99

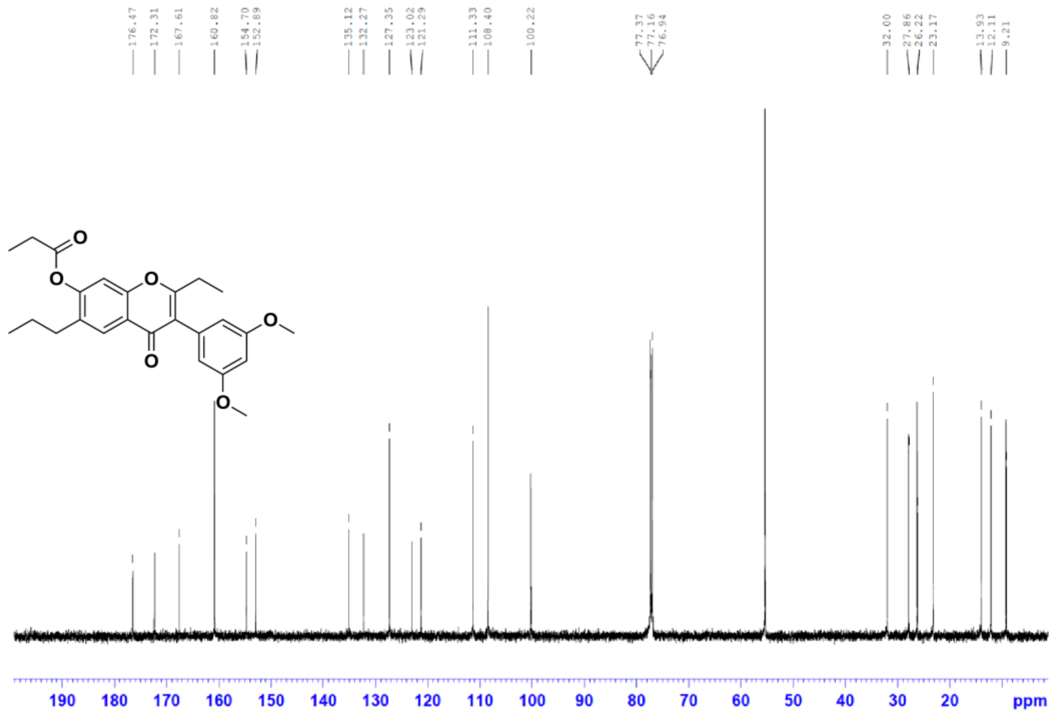
176.47
172.31
167.61
160.82
154.70
152.89
135.12
132.27
127.35
123.02
121.29
111.33
108.40
100.22
77.37
77.16
76.94
32.00
27.86
26.22
23.17
13.93
12.11
9.21
190 180 170 160 150 140 130 120 110 100 90 80 70 60 50 40 30 20 ppm

JG-3-120

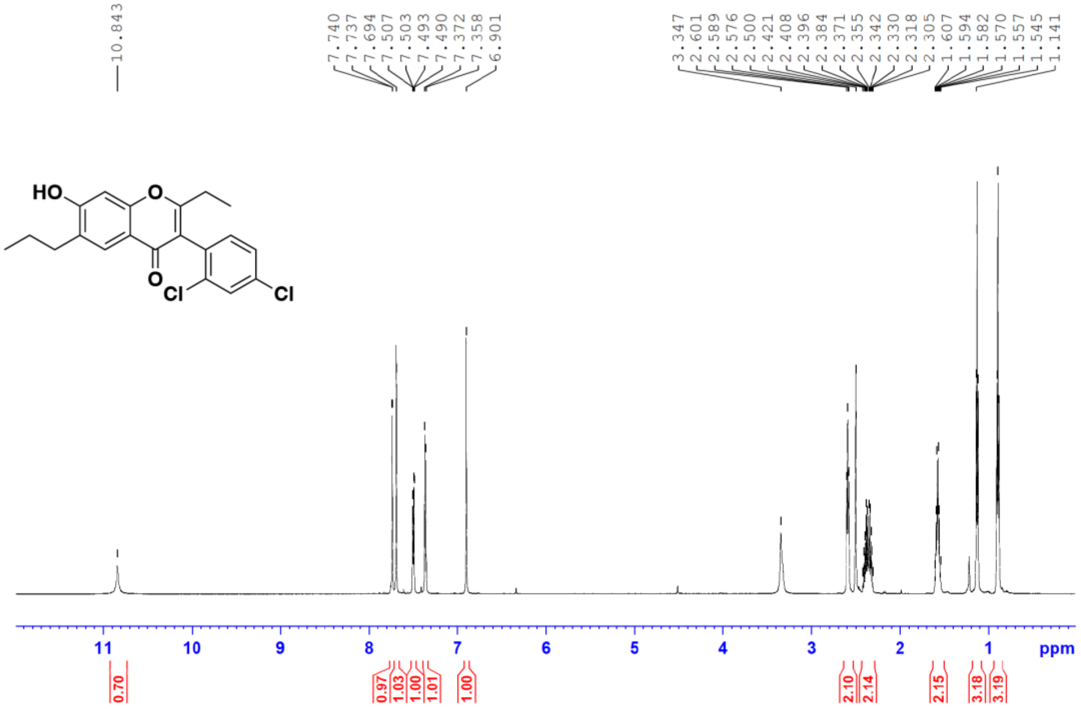
10.843
7.740
7.737
7.694
7.507
7.503
7.493
7.490
7.372
7.358
6.901
3.347
2.601
2.589
2.576
2.500
2.421
2.408
2.396
2.384
2.371
2.355
2.342
2.330
2.318
2.305
1.607
1.594
1.582
1.570
1.557
1.545
1.141
HO
O
O
Cl
Cl
ppm
0.70
0.97
1.03
1.00
1.01
1.00
2.10
2.14
2.15
3.18
3.19

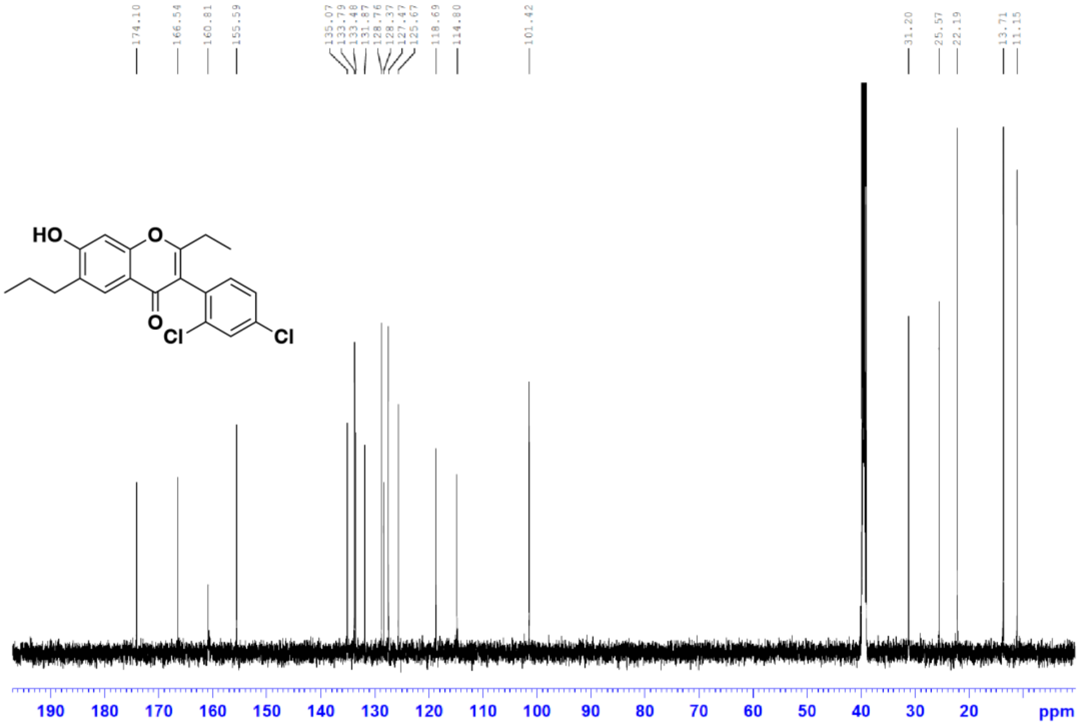
174.10
166.54
160.81
155.59
135.07
133.79
133.48
131.87
128.76
128.37
127.47
125.67
118.69
114.80
101.42
31.20
25.57
22.19
13.71
11.15
HO
O
O
Cl
Cl
ppm

JG-3-121

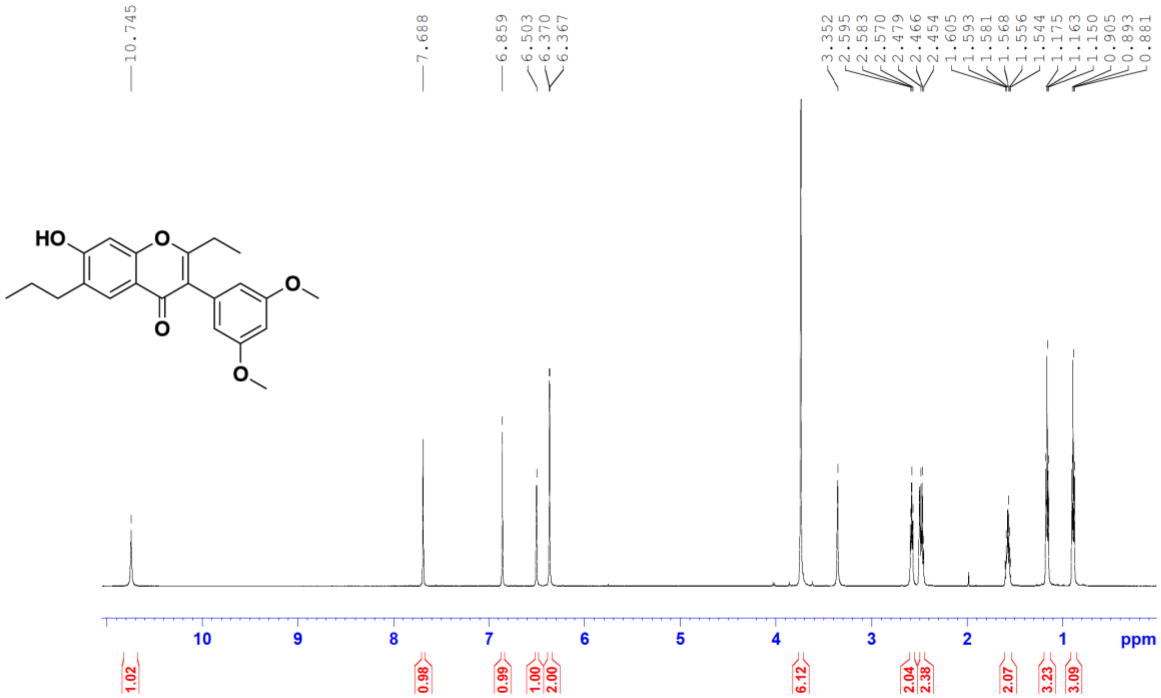

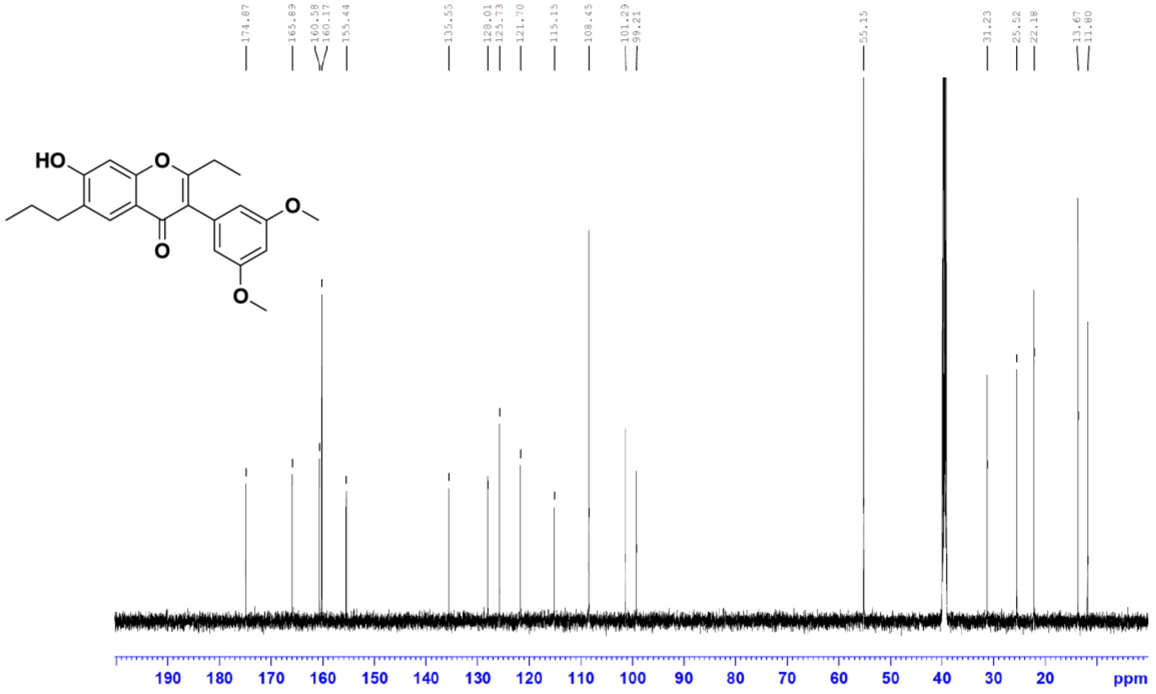

JG-3-127

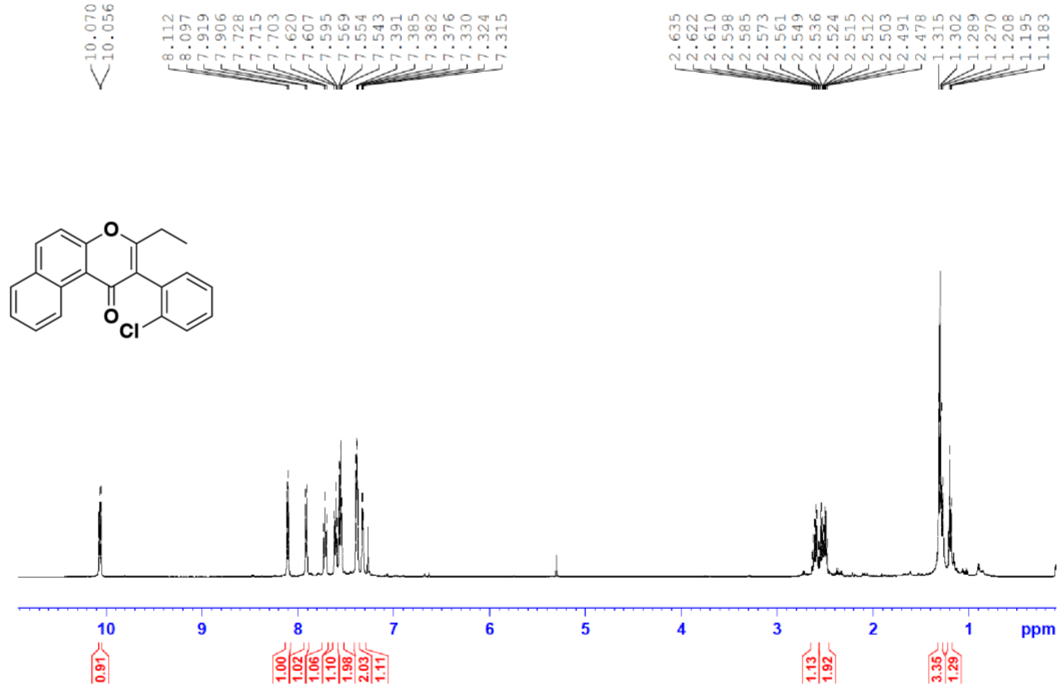
10.070
10.056
8.112
8.097
7.919
7.906
7.728
7.715
7.703
7.620
7.607
7.595
7.569
7.554
7.543
7.391
7.385
7.382
7.376
7.330
7.324
7.315
2.635
2.622
2.610
2.598
2.585
2.573
2.561
2.549
2.536
2.524
2.515
2.512
2.503
2.491
2.478
1.315
1.302
1.289
1.270
1.208
1.195
1.183
O
O
Cl
ppm
0.91
1.00
1.02
1.06
1.10
1.98
2.03
1.11
1.13
1.92
3.35
1.29

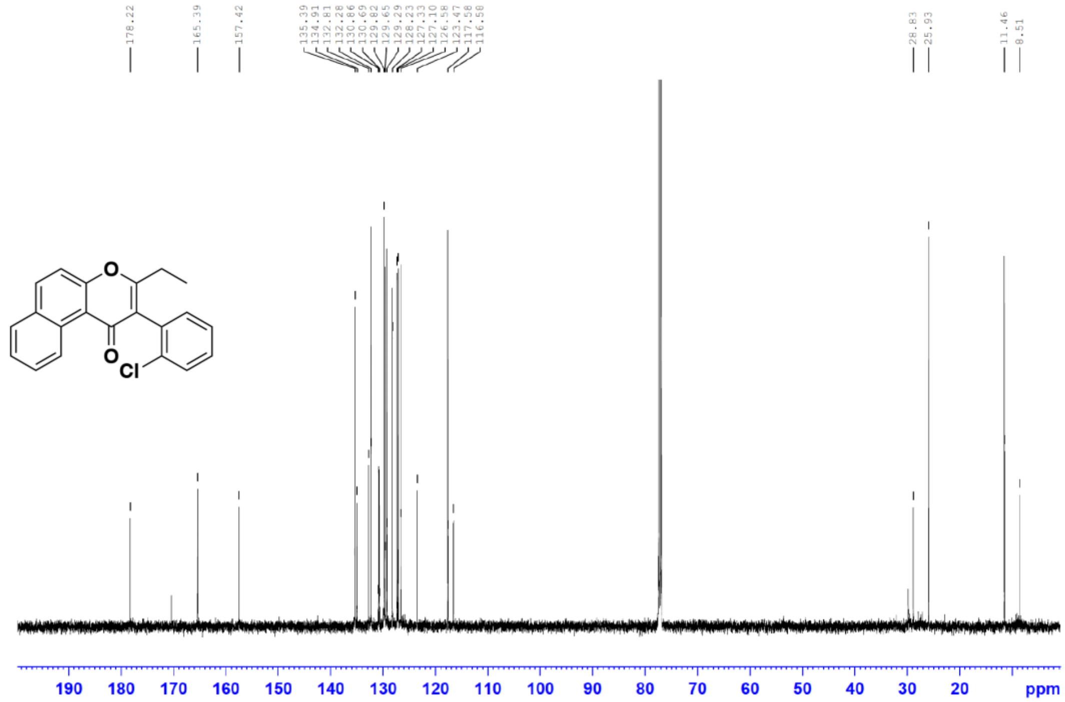
178.22
165.39
157.42
135.39
134.91
132.81
132.28
130.86
129.69
129.82
129.65
129.29
128.23
127.33
127.10
126.58
123.47
117.58
116.58
28.83
25.93
11.46
8.51
O
O
Cl
ppm

JG-3-138

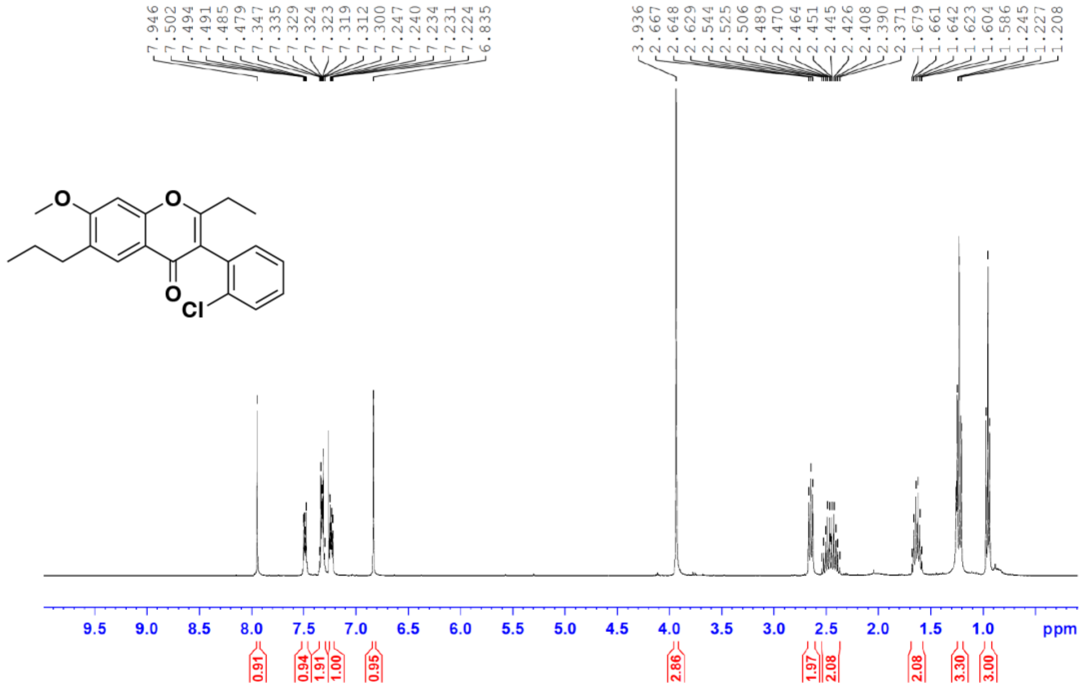
7.946
7.502
7.494
7.491
7.485
7.479
7.347
7.335
7.329
7.324
7.323
7.319
7.312
7.300
7.247
7.240
7.234
7.231
7.224
6.835
3.936
2.667
2.648
2.629
2.544
2.525
2.506
2.489
2.470
2.464
2.451
2.445
2.426
2.408
2.390
2.371
1.679
1.661
1.642
1.623
1.604
1.586
1.245
1.227
1.208
O
O
Cl
9.5 9.0 8.5 8.0 7.5 7.0 6.5 6.0 5.5 5.0 4.5 4.0 3.5 3.0 2.5 2.0 1.5 1.0 ppm
0.91
0.94
1.91
1.00
0.95
2.86
1.97
2.08
2.08
3.30
3.00

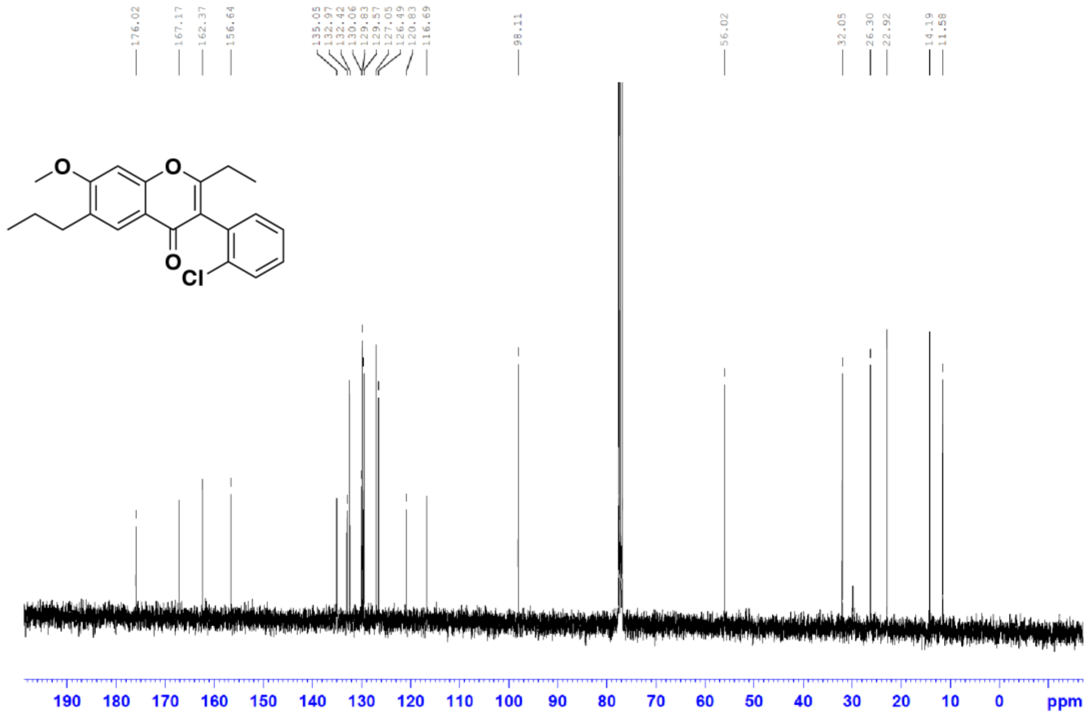
176.02
167.17
162.37
156.64
135.05
132.97
132.42
130.06
129.83
129.57
127.05
126.49
120.83
116.69
98.11
56.02
32.05
26.30
22.92
14.19
11.58
O
O
Cl
190 180 170 160 150 140 130 120 110 100 90 80 70 60 50 40 30 20 10 0 ppm

JG-3-139

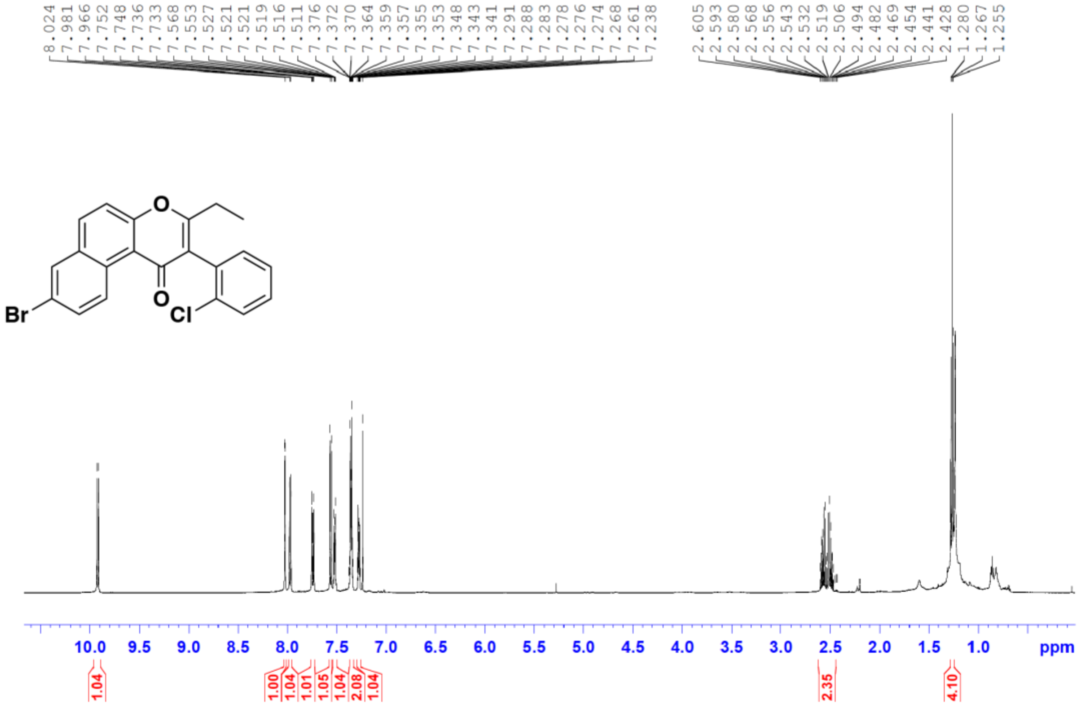

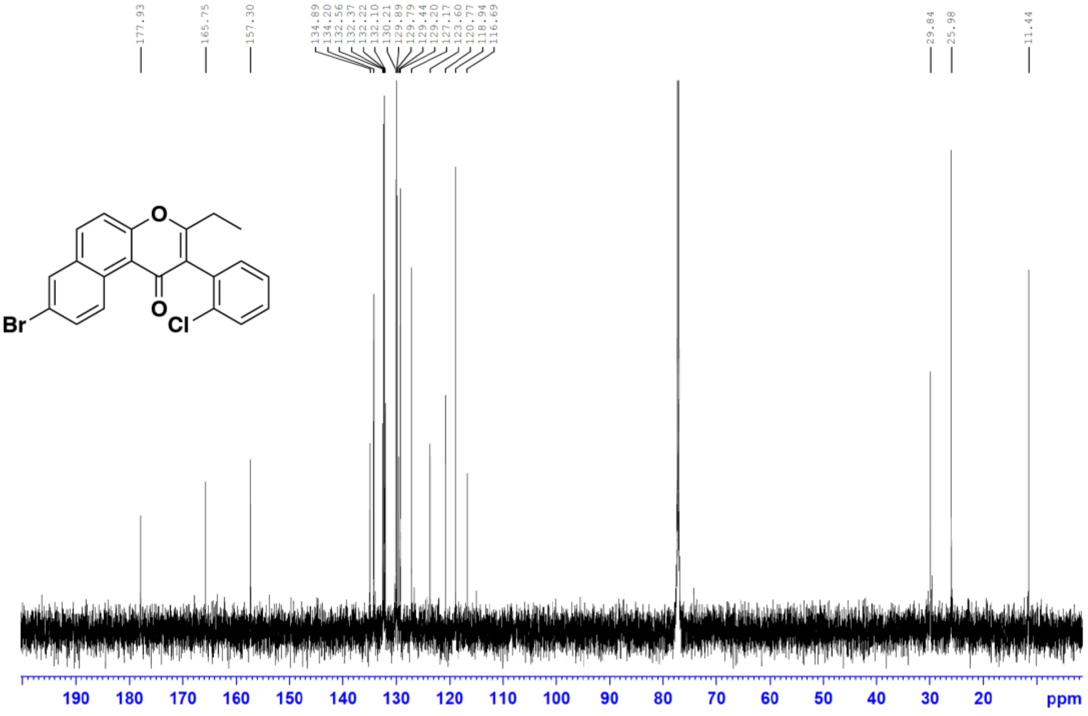

JG-3-154

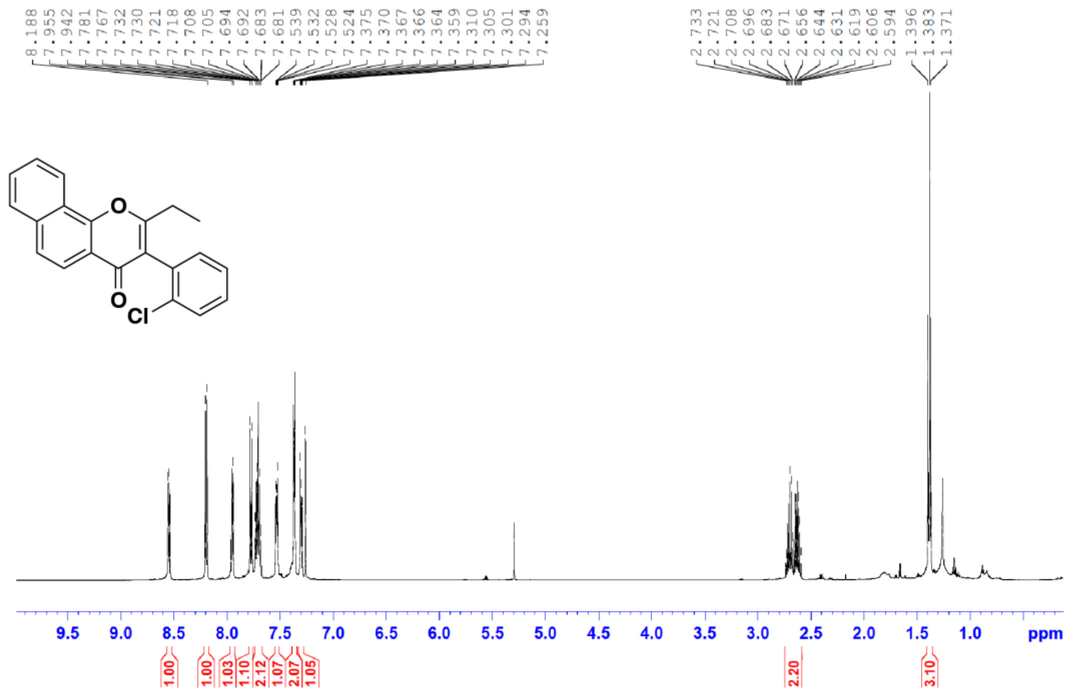
8.188
7.955
7.942
7.781
7.767
7.732
7.730
7.721
7.718
7.708
7.705
7.694
7.692
7.683
7.681
7.539
7.532
7.528
7.524
7.375
7.370
7.367
7.366
7.364
7.359
7.310
7.305
7.301
7.294
7.259
2.733
2.721
2.708
2.696
2.683
2.671
2.656
2.644
2.631
2.619
2.606
2.594
1.396
1.383
1.371
O
Cl
9.5 9.0 8.5 8.0 7.5 7.0 6.5 6.0 5.5 5.0 4.5 4.0 3.5 3.0 2.5 2.0 1.5 1.0 ppm
1.00
1.00
1.03
1.10
2.12
1.07
2.07
1.05
2.20
3.10

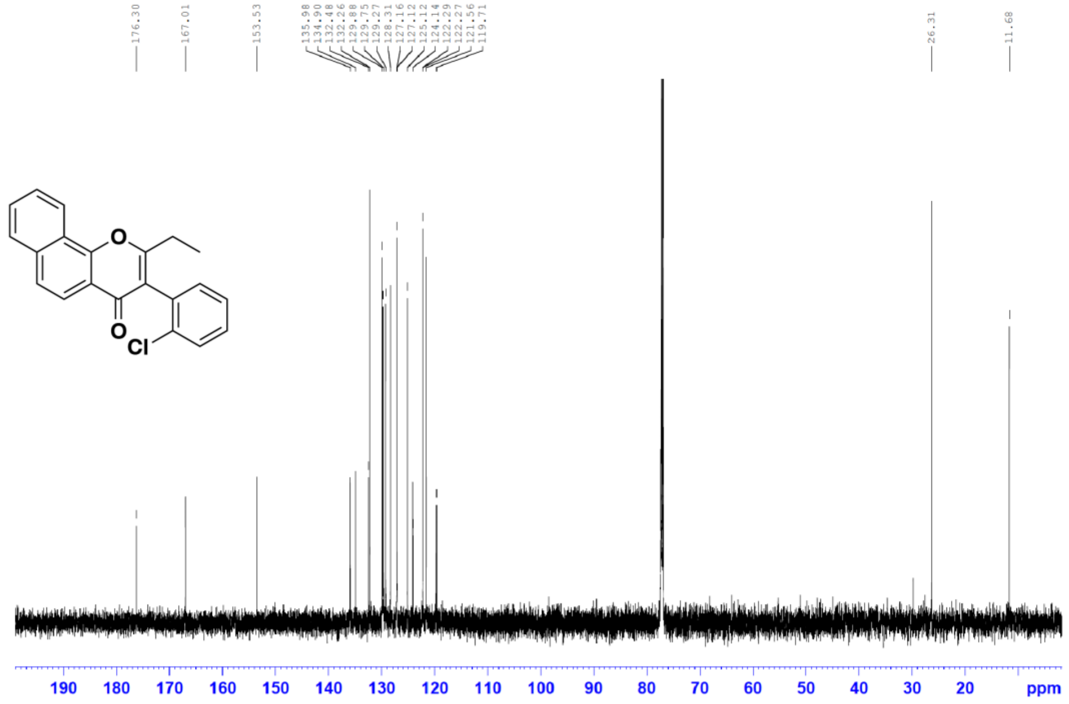
176.30
167.01
153.53
135.98
134.90
132.48
132.26
129.88
129.75
129.27
128.31
127.16
127.12
125.12
124.14
122.29
122.27
121.56
119.71
26.31
11.68
O
Cl
190 180 170 160 150 140 130 120 110 100 90 80 70 60 50 40 30 20 ppm

JG-3-155

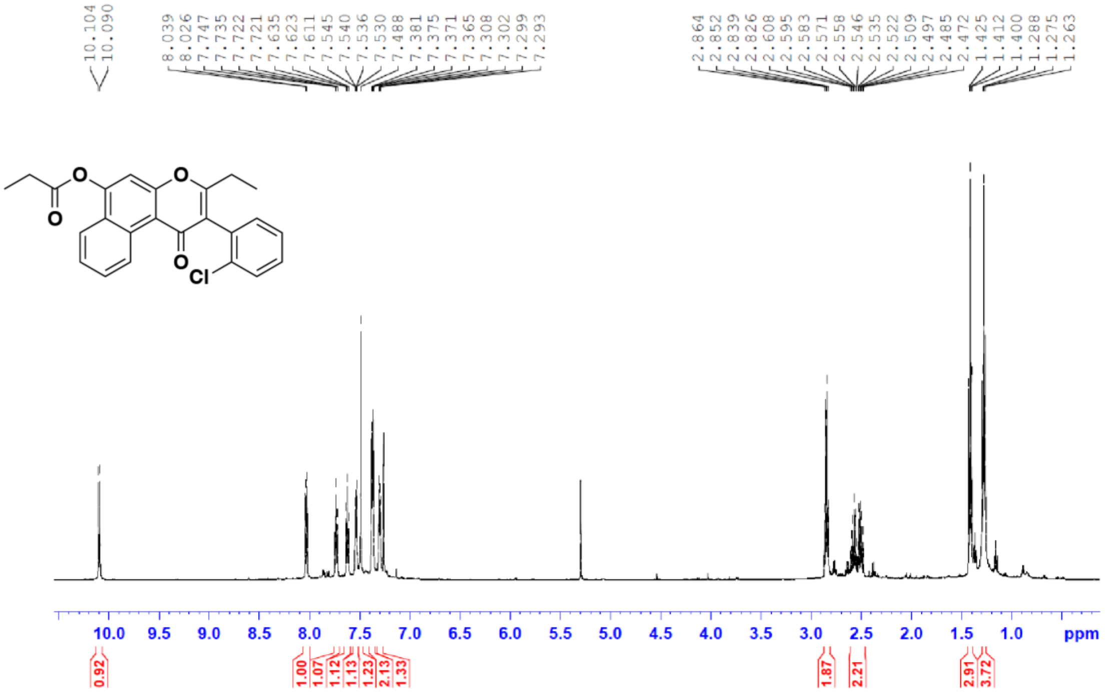
10.104
10.090
8.039
8.026
7.747
7.735
7.722
7.721
7.635
7.623
7.611
7.545
7.540
7.536
7.530
7.488
7.381
7.375
7.371
7.365
7.308
7.302
7.299
7.293
2.864
2.852
2.839
2.826
2.608
2.595
2.583
2.571
2.558
2.546
2.535
2.522
2.509
2.497
2.485
2.472
1.425
1.412
1.400
1.288
1.275
1.263
O
O
O
O
Cl
ppm
0.92
1.00
1.07
1.12
1.13
1.13
1.23
2.13
1.33
1.87
2.21
2.91
3.72

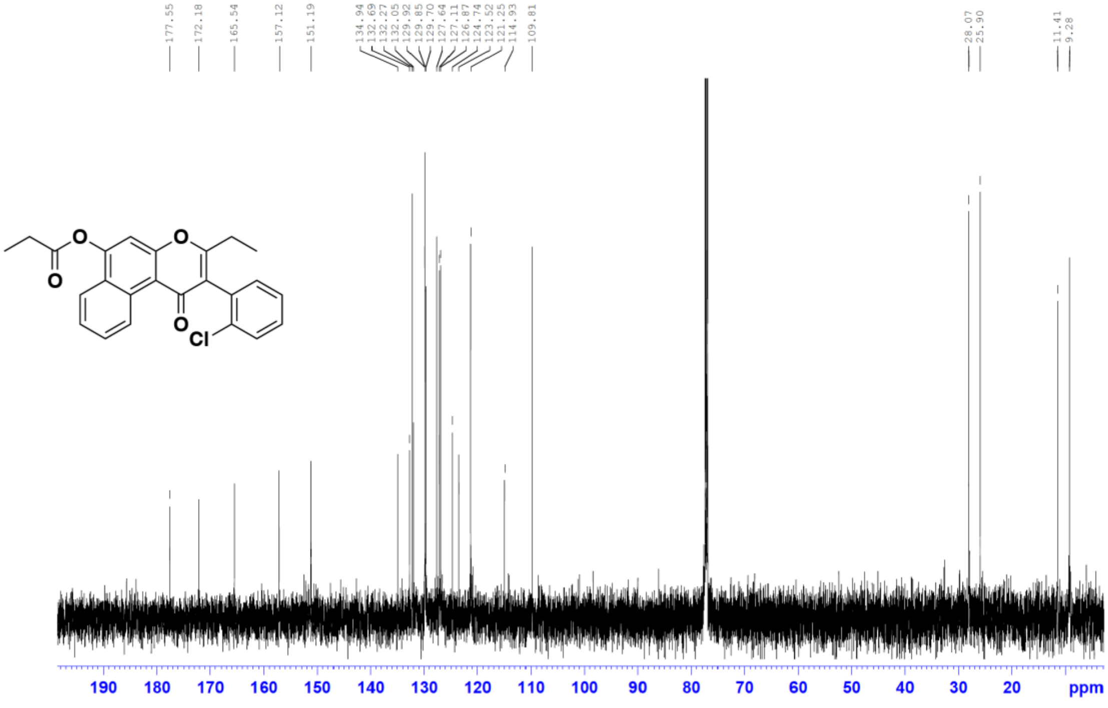
177.55
172.18
165.54
157.12
151.19
134.94
132.69
132.27
132.05
129.92
129.85
129.70
127.64
127.11
126.87
124.74
123.52
121.25
114.93
109.81
28.07
25.90
11.41
9.28
O
O
O
O
Cl
ppm

JG-3-156

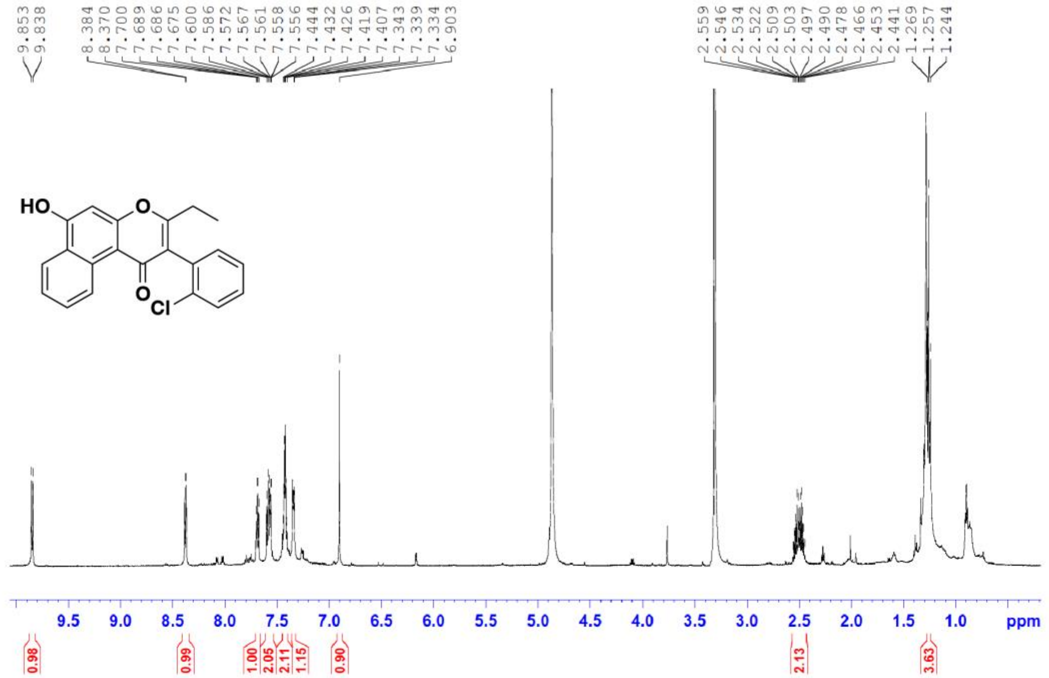
9.853
9.838
8.384
8.370
7.700
7.689
7.686
7.675
7.600
7.586
7.572
7.567
7.561
7.558
7.556
7.444
7.432
7.426
7.419
7.407
7.343
7.339
7.334
6.903
2.559
2.546
2.534
2.522
2.509
2.503
2.497
2.490
2.478
2.466
2.453
2.441
1.269
1.257
1.244
HO
O
O
Cl
ppm
0.98
0.99
1.00
2.05
2.11
1.15
0.90
2.13
3.63

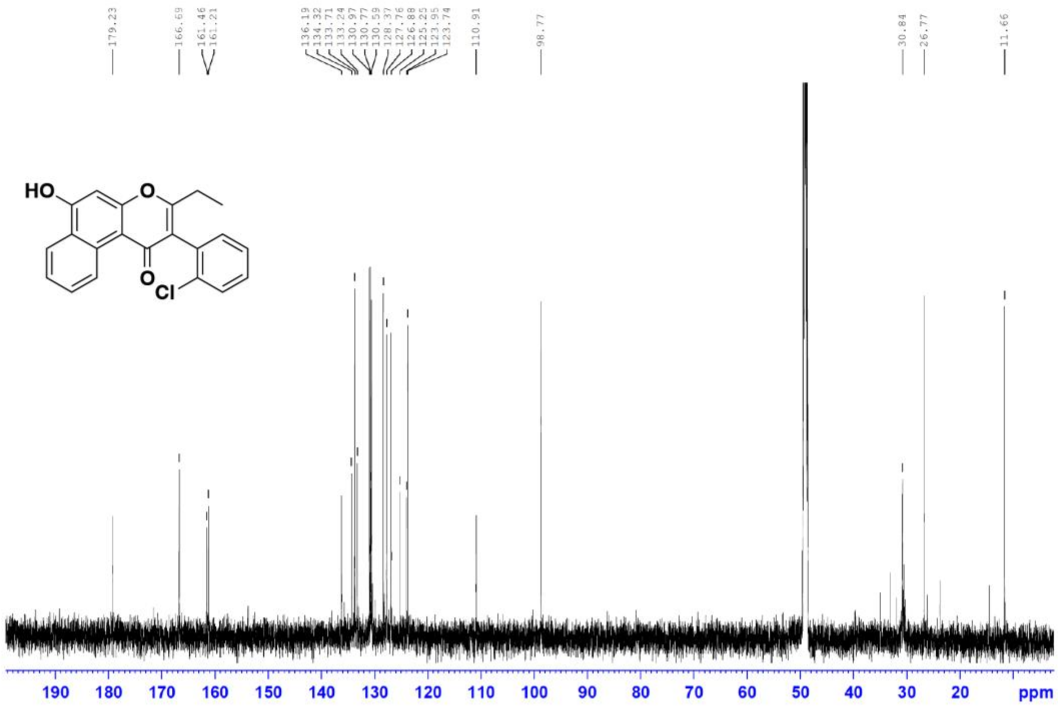
179.23
166.69
161.46
161.21
136.19
134.32
133.71
133.24
130.97
130.77
130.59
128.37
127.76
126.88
125.25
123.95
123.74
110.91
98.77
30.84
26.77
11.66
HO
O
O
Cl
ppm

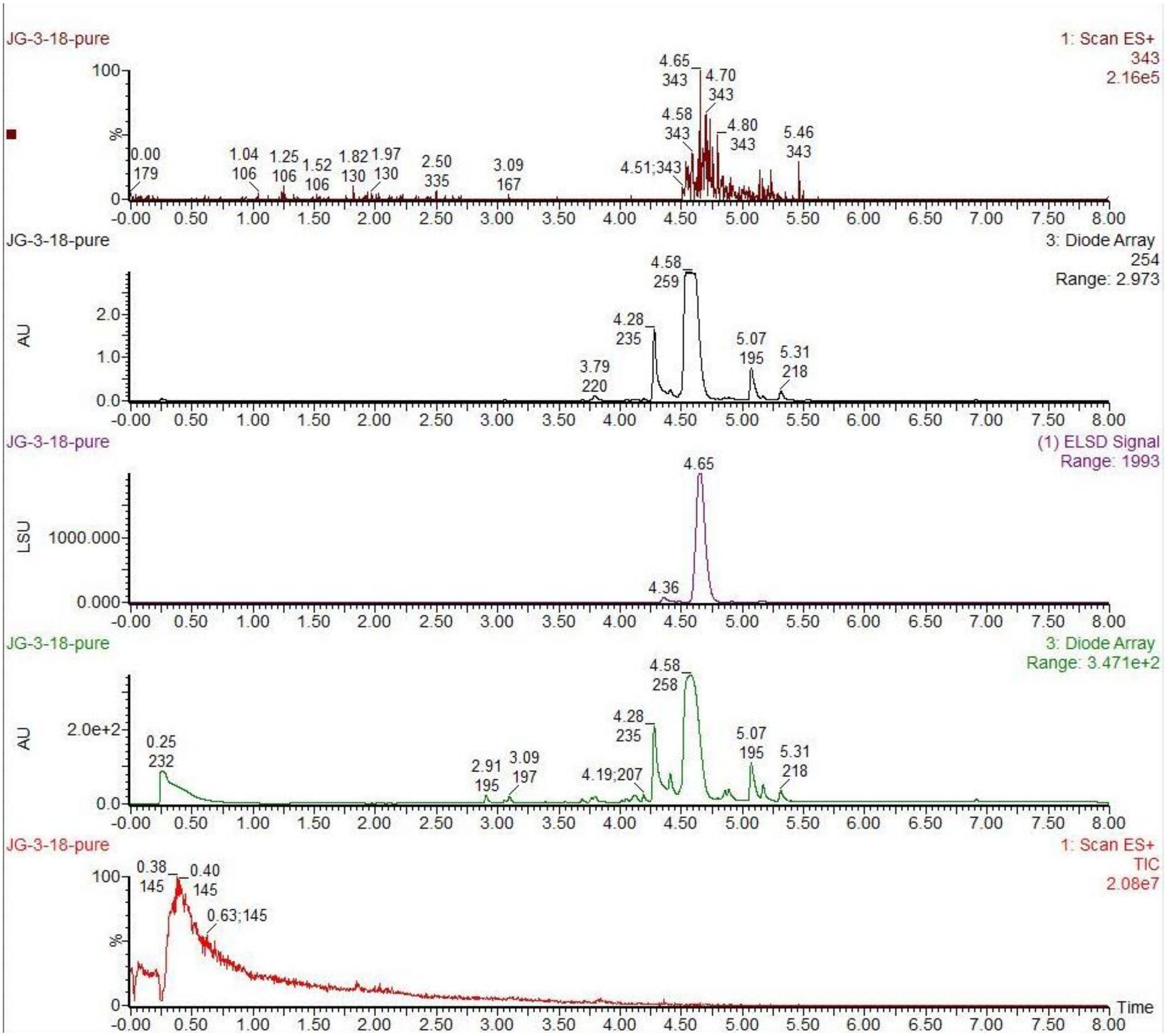
JG-3-18-pure
1: Scan ES+
343
2.16e5
4.65
343
4.70
343
4.58
343
4.80
343
5.46
343
0.00
179
1.04
106
1.25
106
1.52
106
1.82
130
1.97
130
2.50
335
3.09
167
4.51;343
3: Diode Array
254
Range: 2.973
4.58
259
4.28
235
5.07
195
5.31
218
3.79
220
AU
(1) ELSD Signal
Range: 1993
4.65
4.36
LSU
3: Diode Array
Range: 3.471e+2
4.58
258
4.28
235
0.25
232
2.91
195
3.09
197
4.19;207
5.07
195
5.31
218
1: Scan ES+
TIC
2.08e7
0.38
145
0.40
145
0.63;145
Time

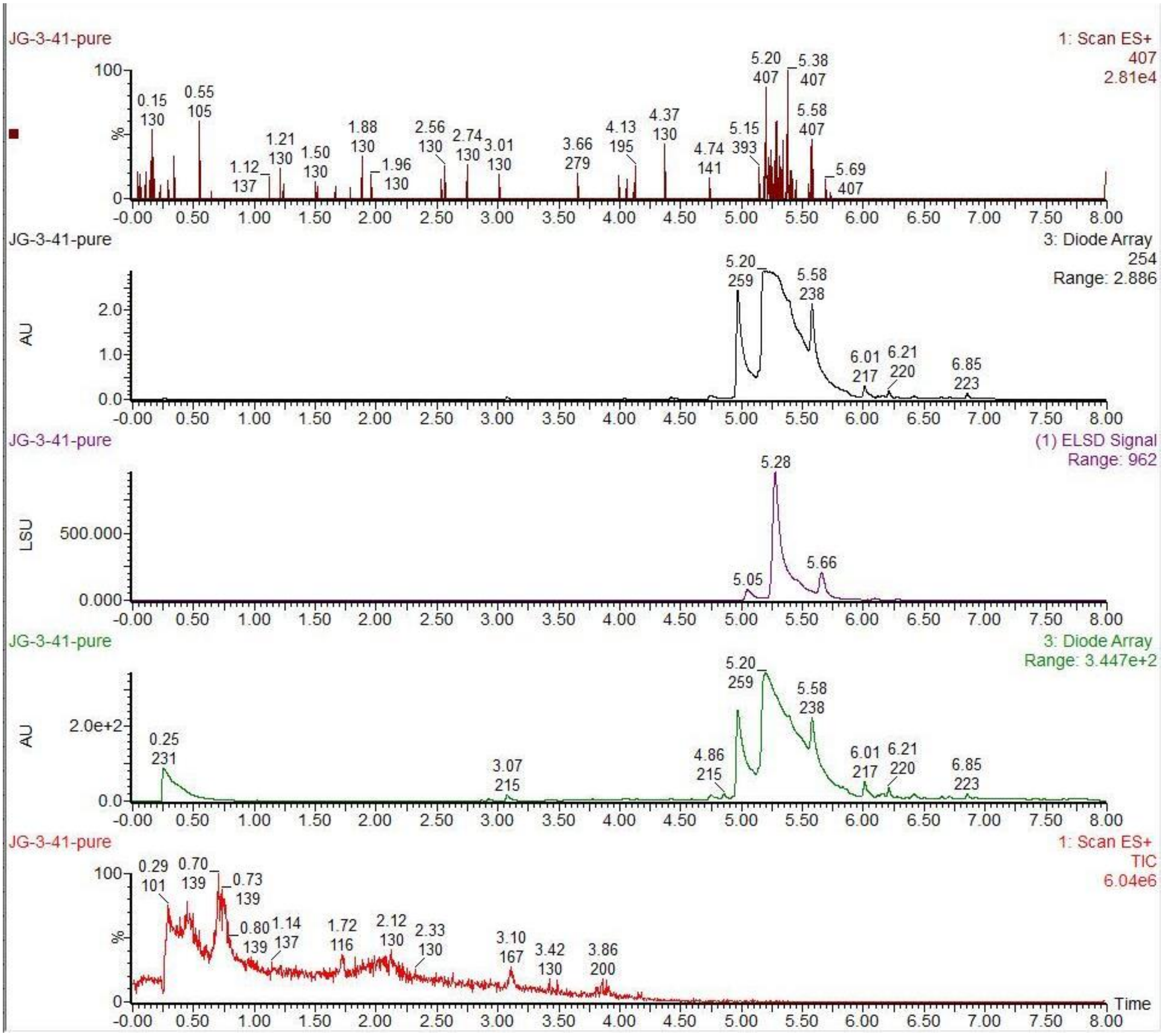
JG-3-41-pure
1: Scan ES+
407
2.81e4
%
JG-3-41-pure
3: Diode Array
254
Range: 2.886
AU
JG-3-41-pure
(1) ELSD Signal
Range: 962
LSU
JG-3-41-pure
3: Diode Array
Range: 3.447e+2
AU
JG-3-41-pure
1: Scan ES+
TIC
6.04e6
%
Time

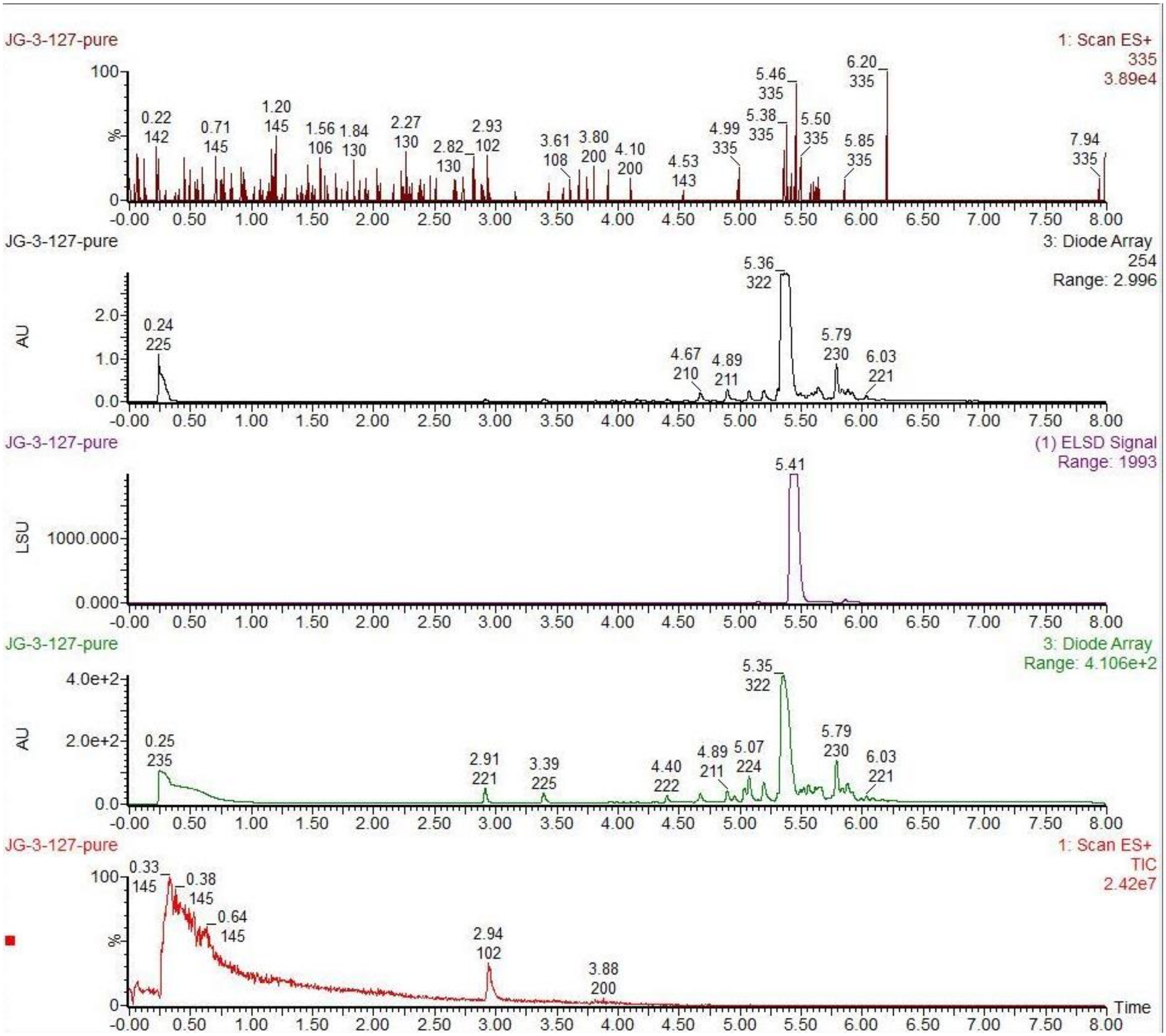
JG-3-127-pure
1: Scan ES+
335
3.89e4
0.22 142
0.71 145
1.20 145
1.56 106
1.84 130
2.27 130
2.82 130
2.93 102
3.61 108
3.80 200
4.10 200
4.53 143
4.99 335
5.38 335
5.46 335
5.50 335
5.85 335
6.20 335
7.94 335
JG-3-127-pure
3: Diode Array
254
Range: 2.996
AU
0.24 225
4.67 210
4.89 211
5.36 322
5.79 230
6.03 221
JG-3-127-pure
(1) ELSD Signal
Range: 1993
LSU
5.41
JG-3-127-pure
3: Diode Array
Range: 4.106e+2
AU
0.25 235
2.91 221
3.39 225
4.40 222
4.89 211
5.07 224
5.35 322
5.79 230
6.03 221
JG-3-127-pure
1: Scan ES+
TIC
2.42e7
0.33 145
0.38 145
0.64 145
2.94 102
3.88 200
Time

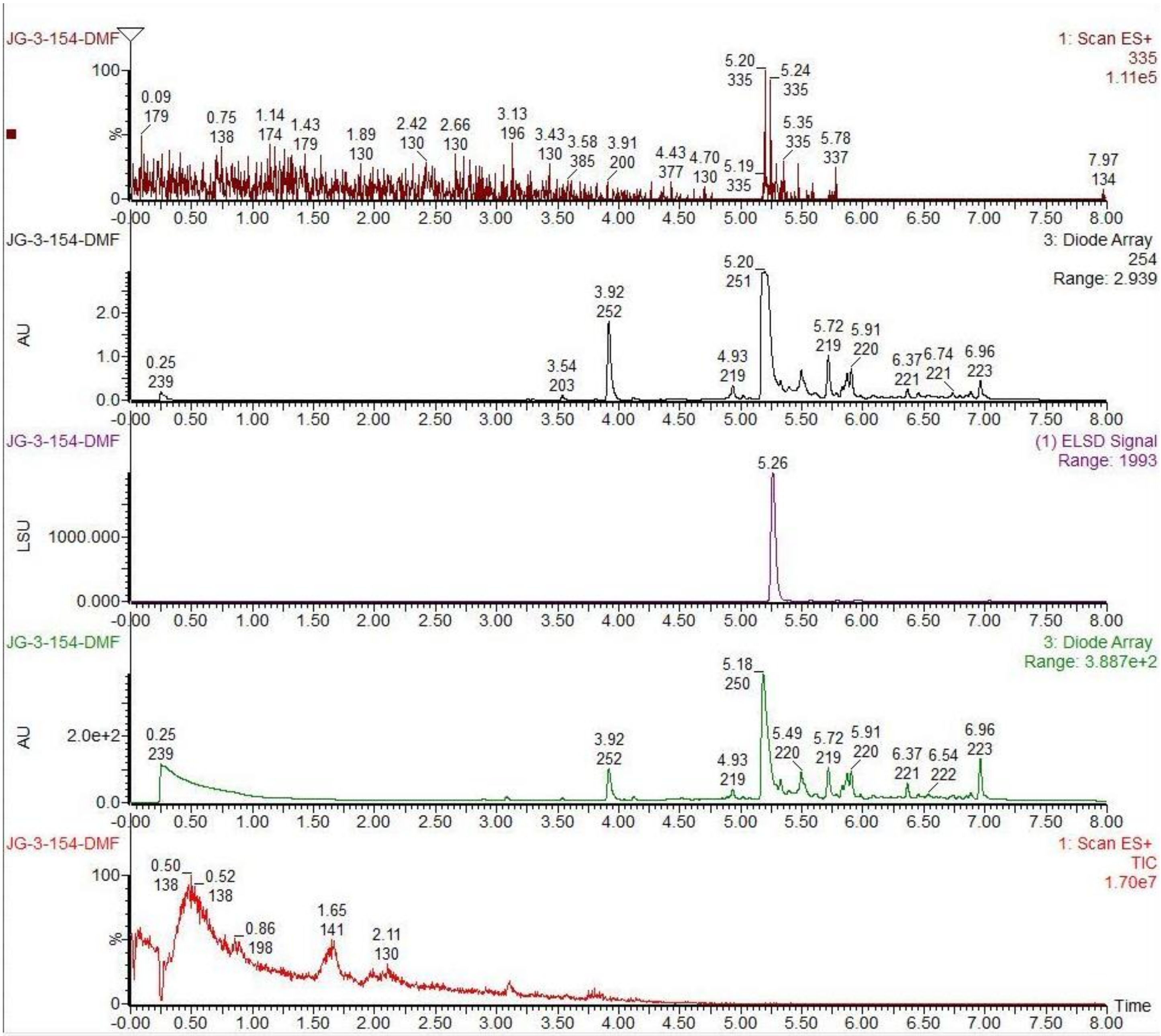
JG-3-154-DMF
1: Scan ES+
335
1.11e5
3: Diode Array
254
Range: 2.939
(1) ELSD Signal
Range: 1993
3: Diode Array
Range: 3.887e+2
1: Scan ES+
TIC
1.70e7
AU
LSU
%
Time
